# Supplementary material for: Systematic Guidelines for Effective Utilization of COVID-19 Databases in Genomic, Epidemiologic, and Clinical Research
Source: Viruses. 2023 Mar 6;15(3):692. doi: 10.3390/v15030692 (PMC10059256; doi:10.3390/v15030692)
Supplement: Supplementary file 1 [file viruses-15-00692-s001.zip › Supplementary Figures.pdf]

SUPPLEMENTARY FIGURES

Systematic Guidelines for Effective Utilization of COVID-19 Databases in Genomic, Epidemiologic, and Clinical Research

Do Young Seong [1,§], Jongkeun Park [1,§], Kijong Yi[2], and Dongwan Hong [1,3,4]*

[1] Department of Medical Informatics, College of Medicine, Catholic University of Korea, 222 Banpo-daero, Seocho-gu, Seoul 06591, Republic of Korea

[2] Graduate School of Medical Science and Engineering, Korea Advanced Institute and Technology (KAIST), Daejeon 34141, Republic of Korea

[3] Precision Medicine Research Center, College of Medicine, Catholic University of Korea, 222 Banpo-daero, Seocho-gu, Seoul 06591, Republic of Korea

[4] Cancer Evolution Research Center, College of Medicine, Catholic University of Korea, 222 Banpo-daero, Seocho-gu, Seoul 06591, Republic of Korea

§ These authors contributed equally to this study and should be considered co-first authors

***** Correspondence: dwhong@catholic.ac.kr; Tel.: +82-2-3147-8424

Table of Contents

Supplementary Figures

Figure S1. COVID-19 data amount and publications collected from January 2020 to October 2022.

Figure S2. Internally developed tools and sublinks of COVID-19 databases.

Figure S3. Comprehensive utilization of COVID-19 databases 5.

Figure S4. Comprehensive utilization of COVID-19 databases 6.

Supplementary Figures

a

b

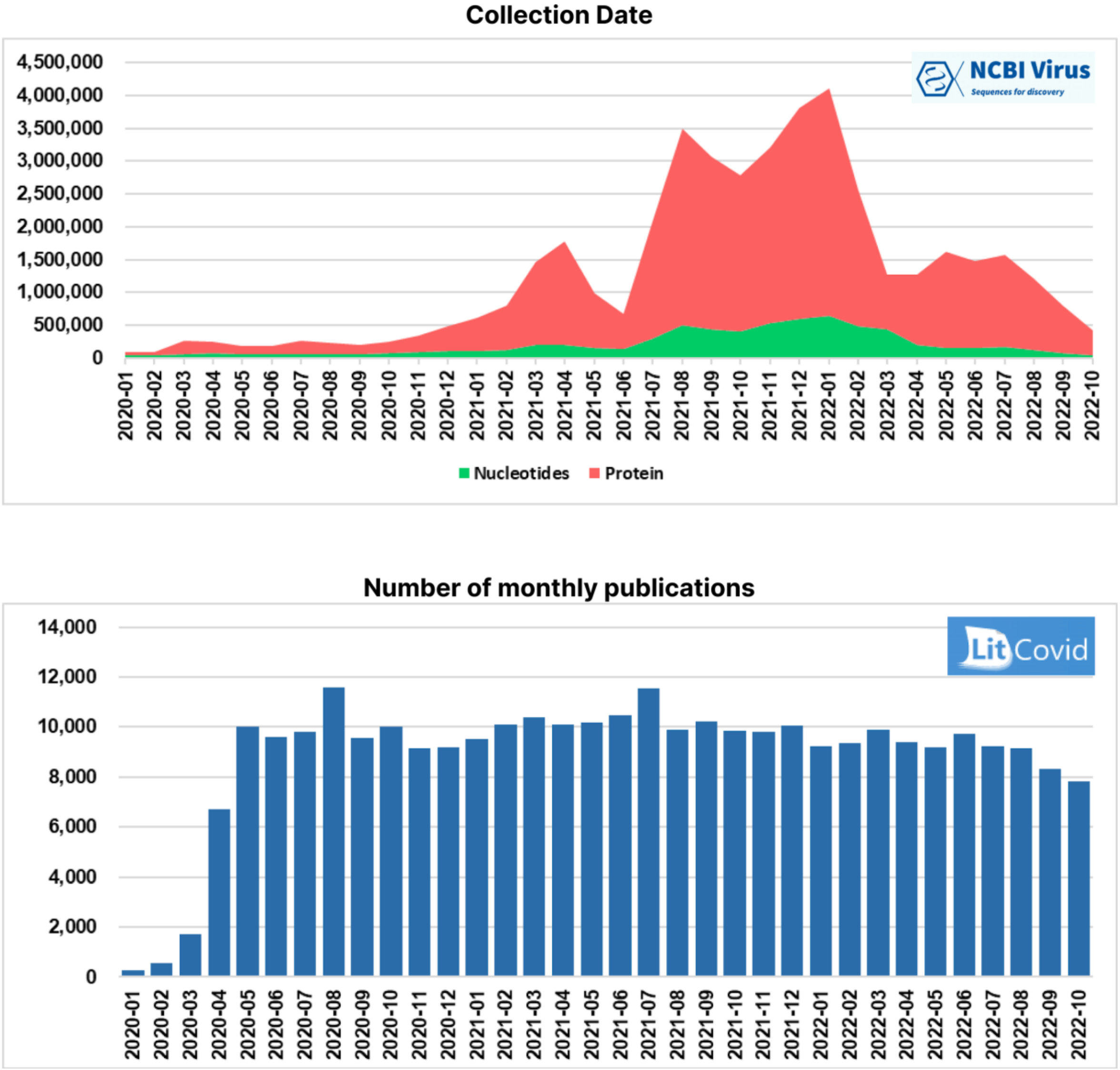

Figure S1. COVID-19 data amount and publications collected from January 2020 to October 2022.

a) Monthly number of nucleotides and protein collection, from NCBI Virus. b) Monthly number of publications submitted related to COVID-19, organized by LitCovid

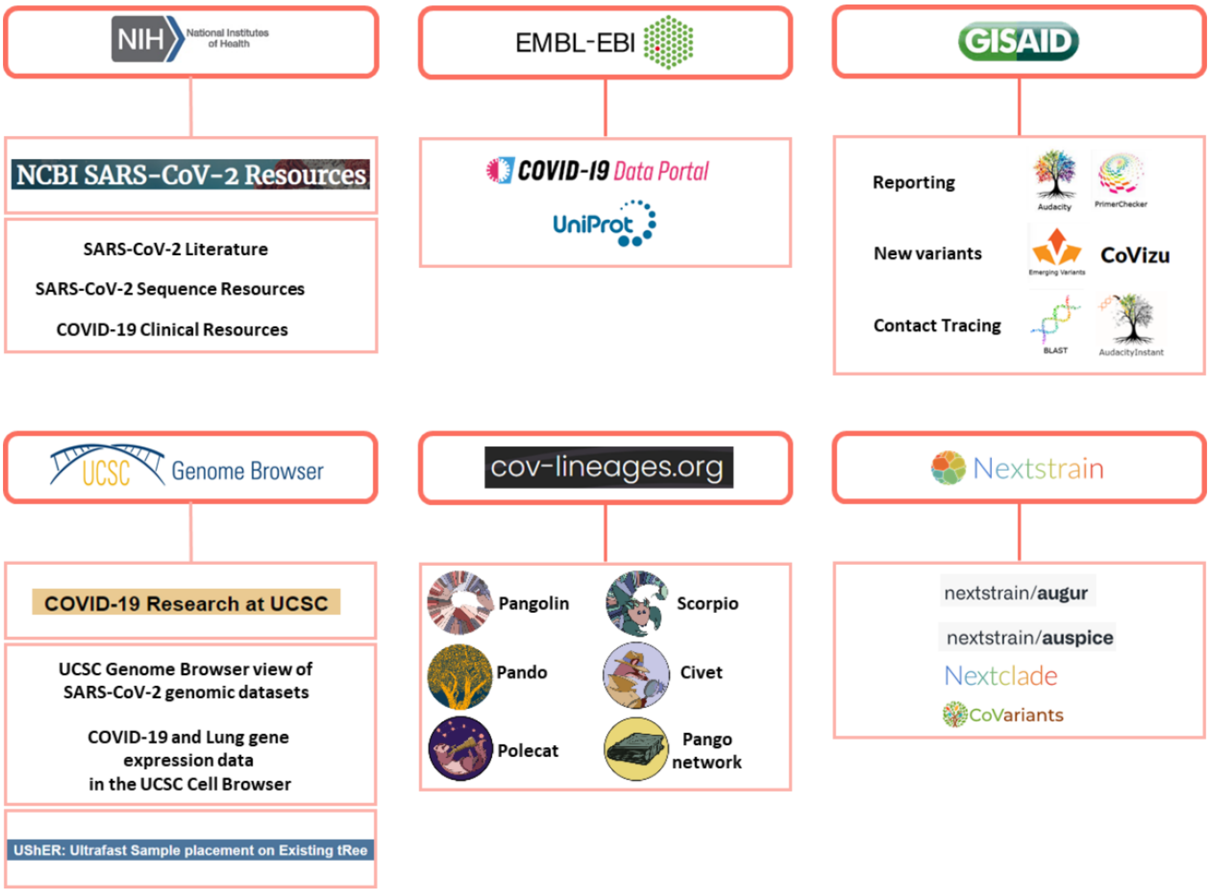

Figure S2. Internally developed tools and sublinks of COVID-19 databases.

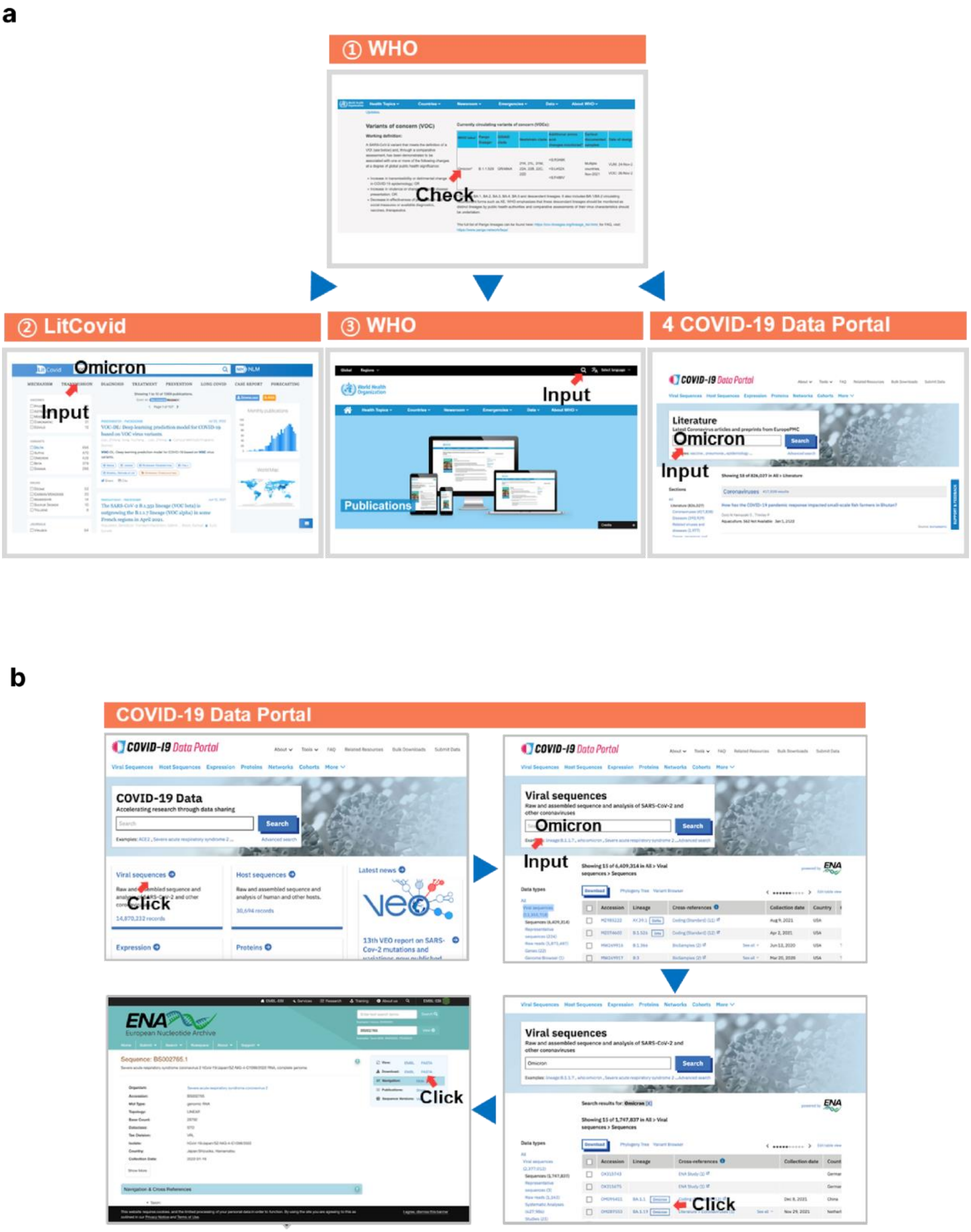

Figure S3. Comprehensive utilization of COVID-19 databases 5

a. Currently circulating Variants of Concern and investigation of associated literature

①We can search for the currently circulating VOC Omicron through WHO's Tracking SARS-CoV-2 variants. ② NIH's LitCovid website categorizes its publications into 8 categories: mechanism, transmission, diagnosis, treatment, prevention, long COVID, case report, forecasting. When searching for Omicron, it returns 3,421 results. ③ WHO's publications are separated into 3 categories: book, Journals and series, and WHO guidelines. When searching for Omicron, 100 results are returned. However, since WHO's literature is comprised of journals published by WHO and guidelines for

COVID-19 rather than publication searches. ④ In the case of COVID-19 data portal provided by EMBL-EBI, it categorizes research topics into 6 sections: Coronaviruses, Diseases, Related viruses and diseases, Genes, receptors and antibodies, Supplementary material, and Compound document. When searching for Omicron, Coronaviruses returns 5,587 results, Diseases returns 4,537, Related viruses and diseases returns 20, and Genes, receptors and antibodies returns 354.

b. Investigation of nucleotide levels mutation data through utilization of virus sequencing mutation data

① Click on virus sequence (14,918,306 records) in the COVID-19 Data Portal. Among those, 6,409,314 sequence files are registered, with options such as accession, lineage, cross-reference, collection date, country, and center name. After noting the data users require, downloading the data or clicking accession navigates to European Nucleotide Archive (ENA). ② Search for VOC Omicron in the search bar. By clicking accession, lineage, navigate to ENA page, then click on the upper right to download the virus genome sequence file (FASTA).

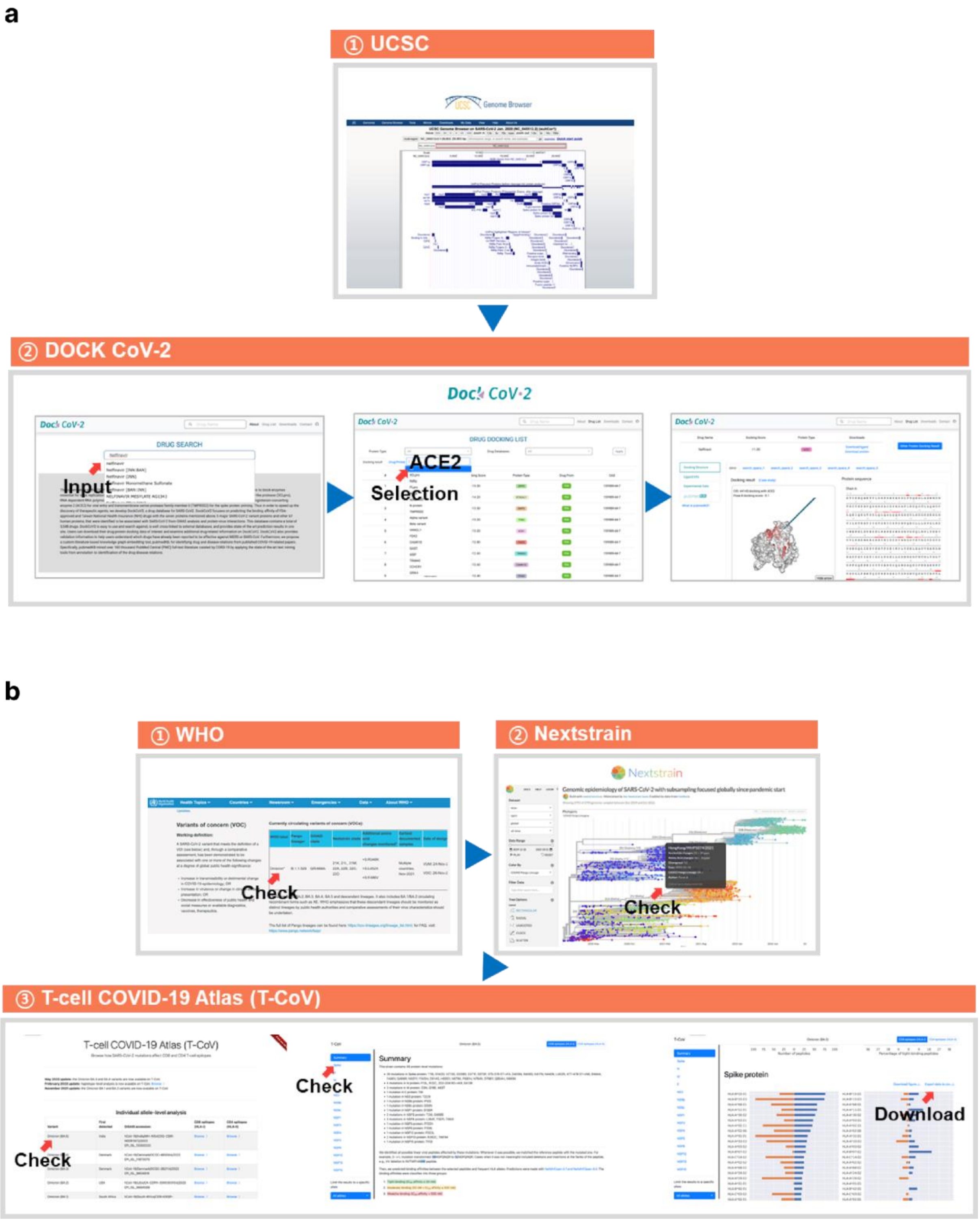

Figure S4. Comprehensive utilization of COVID-19 databases 6

a. Investigation of clinical resources and target for SARS-CoV-2 lineages and structures

① To determine SARS-CoV-2 protein structure and domain, UCSC SARS-CoV-2 genome browser from COVID-19 Research at UCSC was utilized. ② Entering Omicron in the search bar of the Dock CoV-2 website navigates to the Drug Docking List page. In the upper part, by searching for RdRP (RNA-dependent RNA polymerase) through Protein Type, we can check Drug Name, Docking Score, and Drug approval. For RdRP search results, related drugs were Nelfinavir and Nelfinavir Mesylate. Clicking on the particular drug displays the docking results, specifically the docking score and visualization of the docking region. The page also displays drug specific custom - literature-based

knowledge graph through Ligand info, experimental data, and pubmedKB. ③ We investigated clinical trials associated with currently circulating VOC Omicron. ④ clinicalTrials.gov find a study features allows conditional searches through Status, Condition or disease, Other terms, Country, and we can search for Omicron through Condition or disease. 42 Omicron related studies are found, and filtering by status (recruitment, Expanded Access), eligibility Criteria, Study Type, Study Results, Study Phase, and Study Documents according to the purpose of the user is possible.

b. Structural change due to SARS-CoV-2 mutation and connection to candidate T-cell epitopes

① We check current VOCs through WHO's Tracking SARS-CoV-2 variants section for Omicron (Figure 11A). ② Through Nextstrain, we can examine variant data on Omicron from Pango lineage and Nextstrain clade through Latest global analysis. ③ We can use T-cell CD4(HLA-I), CD8(HLA-II) epitopes data from the T-CoV database to predict peptides selected based on WHO label and binding affinity of HLA alleles. Among the VOC Omicron, the most recently global trending variant as of November 2021 BA.5 have summary reports variant data and predictions. For each SARS-CoV-2, Prediction values for T-cell CD4(HLA-I), CD8(HLA-II) epitopes are organized. We can download CD4(HLA-I) and CD8(HLA-II) data from T-cell.
