# Supplementary material for: Systematic Guidelines for Effective Utilization of COVID-19 Databases in Genomic, Epidemiologic, and Clinical Research
Source: Viruses. 2023 Mar 6;15(3):692. doi: 10.3390/v15030692 (PMC10059256; doi:10.3390/v15030692)
Supplement: Supplementary file 1 [file viruses-15-00692-s001.zip › Supplementary Notes.pdf]

SUPPLEMENTARY Notes

Systematic Guidelines for Effective Utilization of COVID-19 Databases in Genomic, Epidemiologic, and Clinical Research

A detailed guide on navigating COVID-19 databases

Do Young Seong [1,§], Jongkeun Park [1,§], Kijong Yi, and Dongwan Hong [1,2,3,*]
[1] Department of Medical Informatics, College of Medicine, Catholic University of Korea, 222 Banpo-daero, Seocho-gu, Seoul 06591, Republic of Korea
[2] Precision Medicine Research Center, College of Medicine, Catholic University of Korea, 222 Banpo-daero, Seocho-gu, Seoul 06591, Republic of Korea
[3] Cancer Evolution Research Center, College of Medicine, Catholic University of Korea, 222 Banpo-daero, Seocho-gu, Seoul 06591, Republic of Korea

§ These authors contributed equally to this study and should be considered co-first authors
* Correspondence: dwhong@catholic.ac.kr; Tel.: +82-2-3147-8424

Contents

Supplementary Figures

Figure S1. GISAID · 3
Figure S2. Cov-lineages.org · 13
Figure S3. COVID CG · 19
Figure S4. COVID-19 Data Portal · 23
Figure S5. Nextstrain · 29
Figure S6. NIH (NCBI SARS-CoV-2 resources) · 34
Figure S7. PDB · 42
Figure S8. World Health Organization (WHO) · 46
Figure S9. University of California, Santa Cruz (UCSC) Genome Browser · · · · · · · 51
Figure S10. Dock CoV-2 · 58
Figure S11. Our World in Data · 61
Figure S12. Johns Hopkins University coronavirus resource center · · · · · · · · · · · · · 64
Figure S13. Immune Escape variants in SARS-CoV-2 (ESC) · · · · · · · · · · · · · · · · · · · 66
Figure S14. T-cell COVID-19 Atlas (T-CoV) · 71
Figure S15. CovInter · 74

Main page

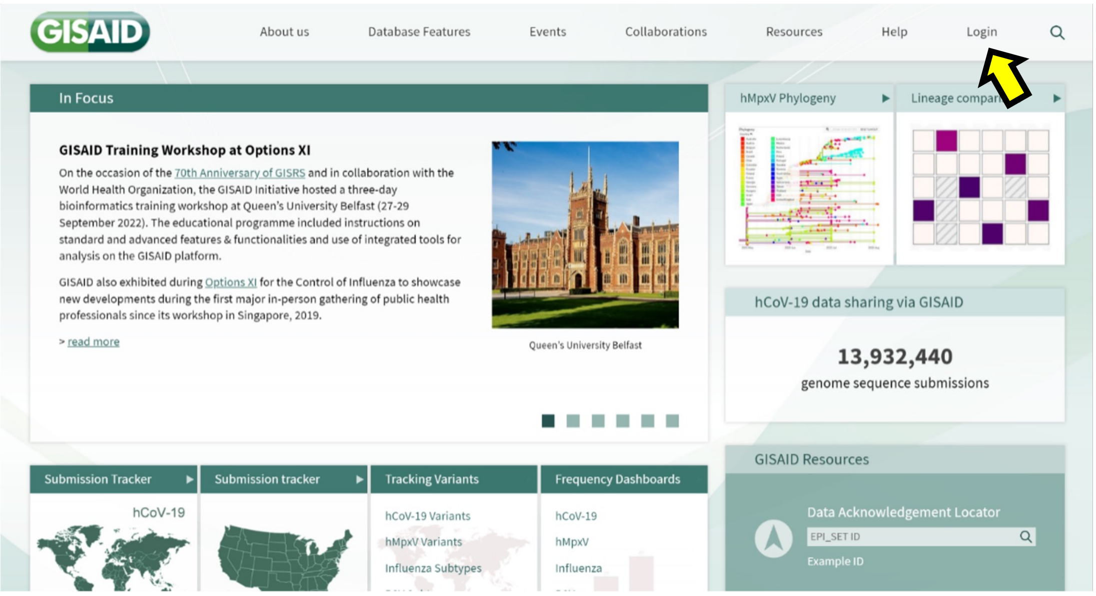

https://gisaid.org/
Global Initiative on Sharing Avian Influenza Data (GISAID) is a database that collects influenza virus sequence data, clinical data, and epidemiological data. Provides data on globally circulating COVID-19.

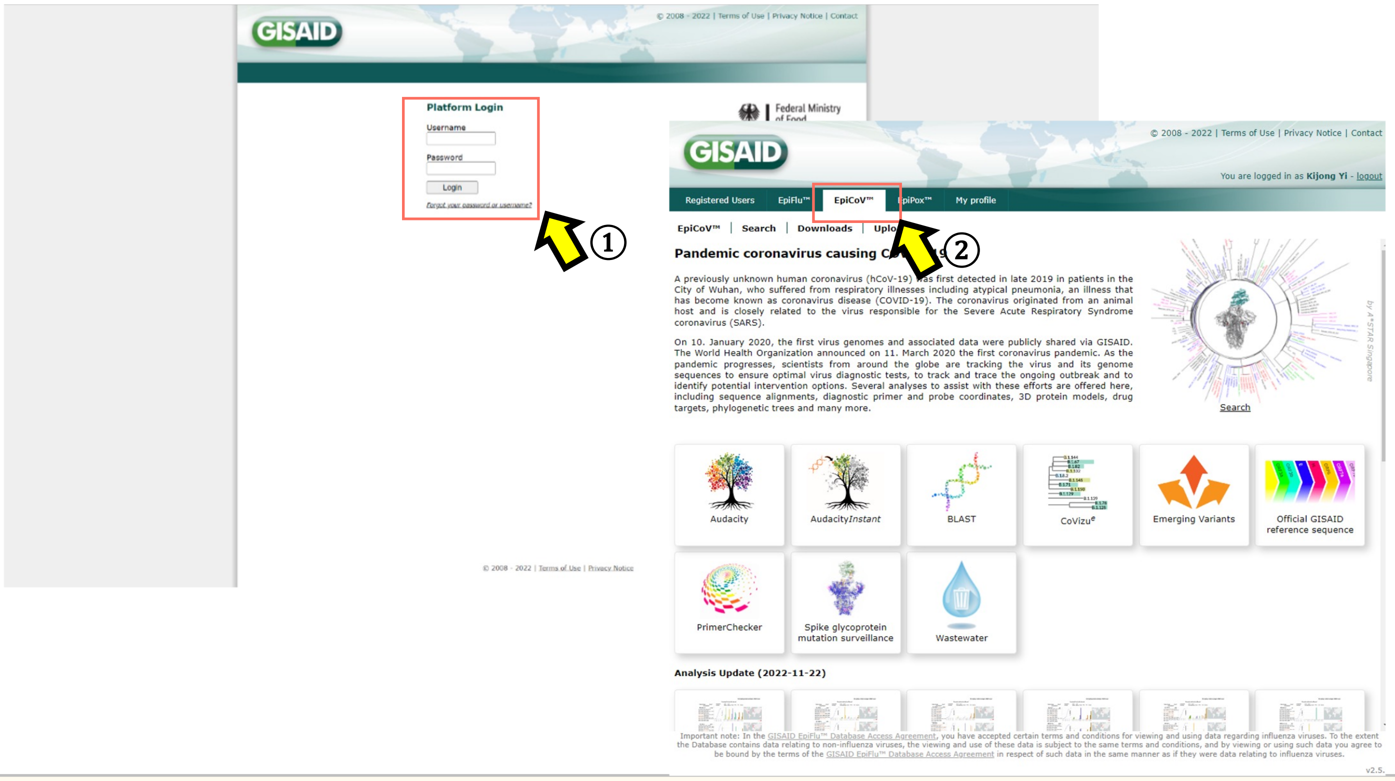

① To perform more data collection and analysis, login is required
② In the upper tab, click on EpiCoV ™

Main page

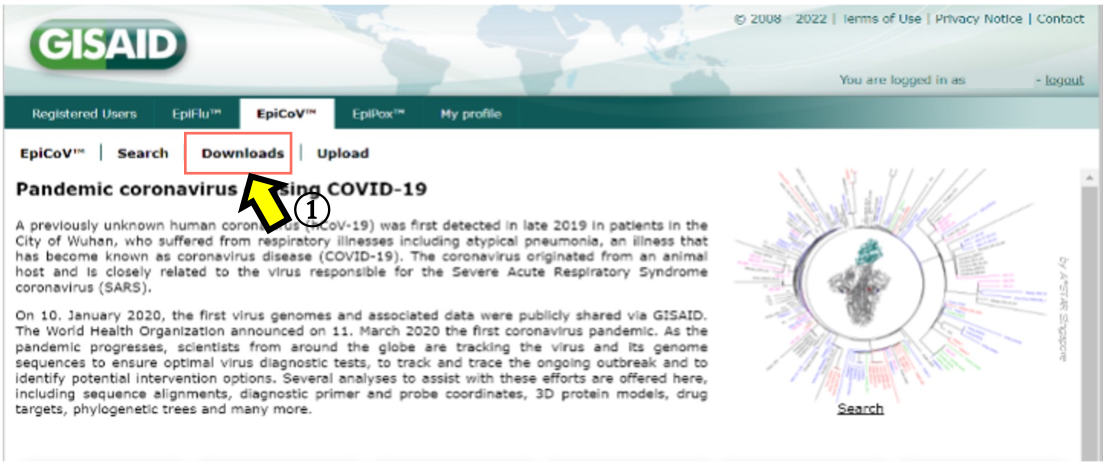

① **To download COVID-19 virus sequence, protein, metadata, etc, click on download from upper tab EpiCoV ™**

Main functions: Clade/variant/lineage

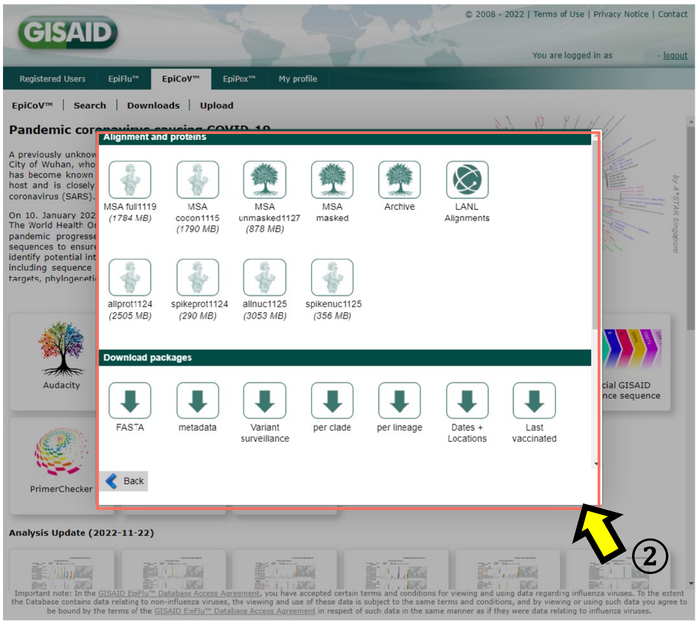

② **From two categories, required data download possible: Alignment and proteins and Download packages**

Main page

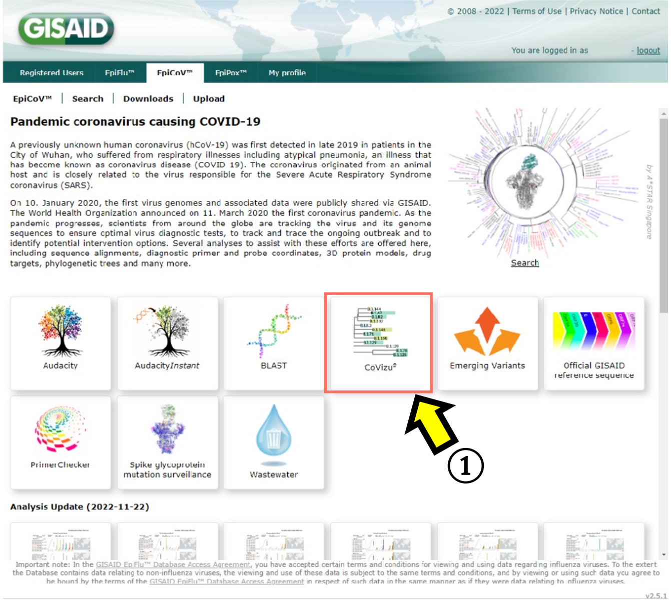

① CoVizuᵉ : using visualization tools, SARS-CoV genomic variation genomes data is provided.

Main functions: Genome browser (sequence)

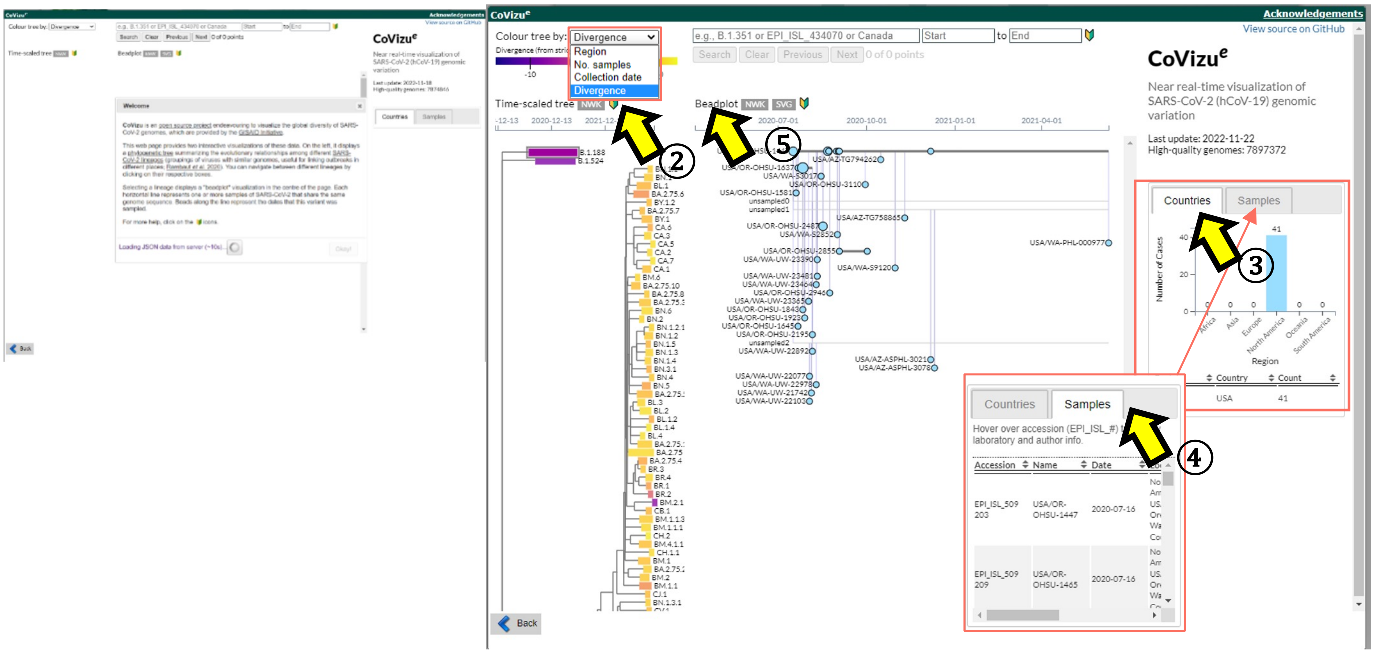

② Phylogenetic tree can be changed dependent on Region, No. samples, Collection date, Divergence
③ Can be selected per Countries
④ sample data
⑤ Through beadplot, sample data can be visualized per collection date

Main page

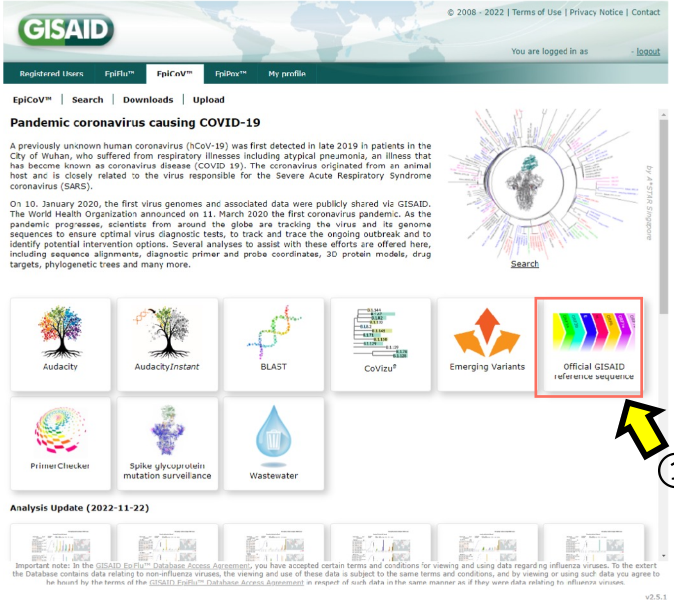

① **Official GISAID reference sequence: SARS-CoV genomic variation genomes data is provided using visualization tools**

Main functions: Genome browser (sequence)

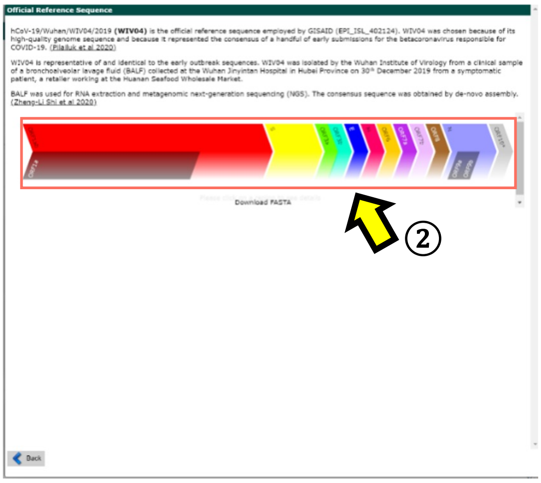

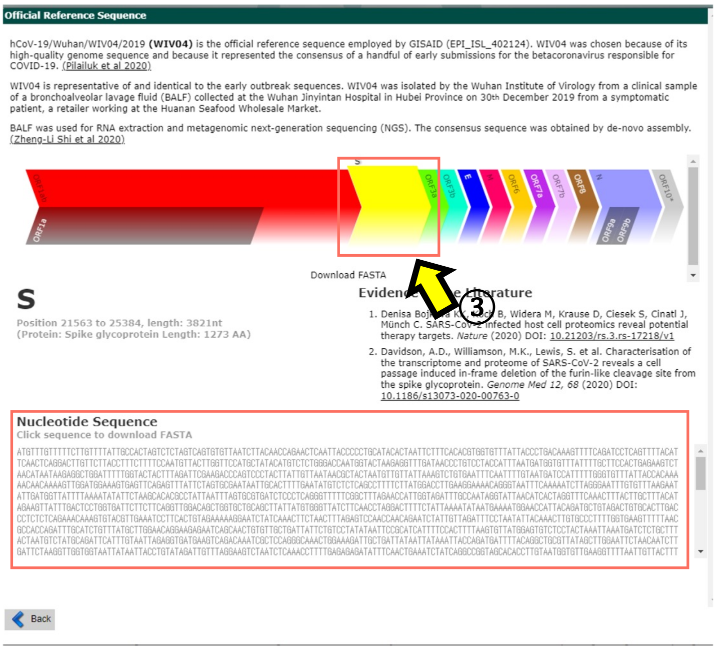

② **Shows SARS-CoV-2 structure**
③ **For example, clicking on S protein shows S protein nucleotide sequence and Download is possible**

Main page

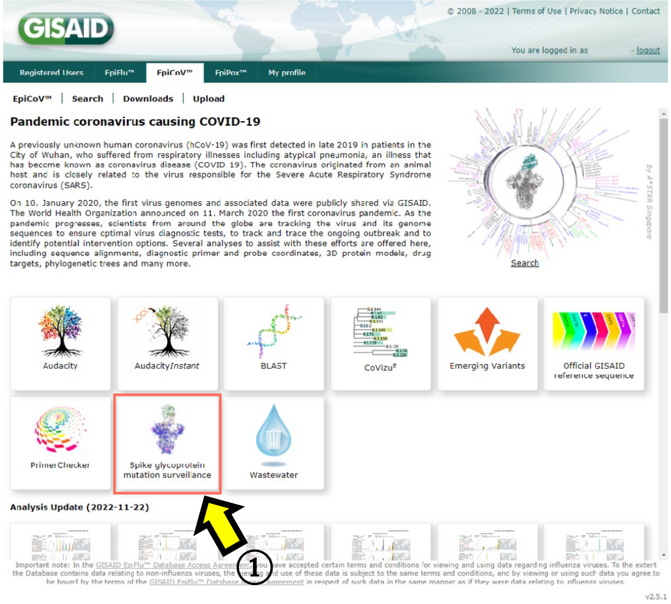

① Spike glycoprotein mutation surveillance shows location data for protein 3D structure in Spike glycoprotein mutation data

Main functions: Protein structure

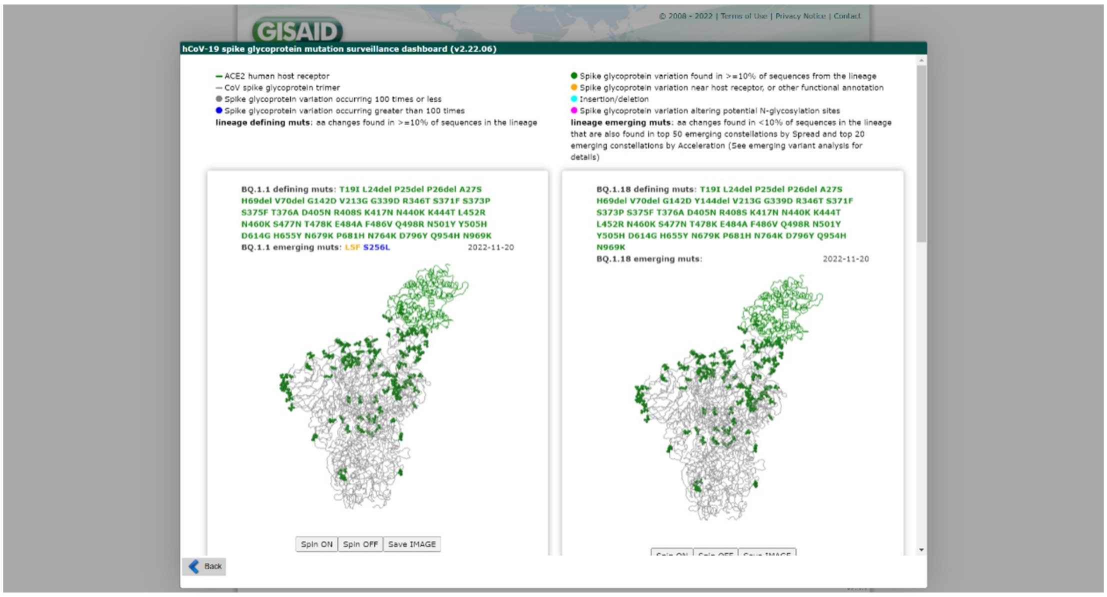

From Spike glycoprotein 3D structure, for mutation function, frequency, type, etc, location can be viewed with colors in the 3D structure

Main page

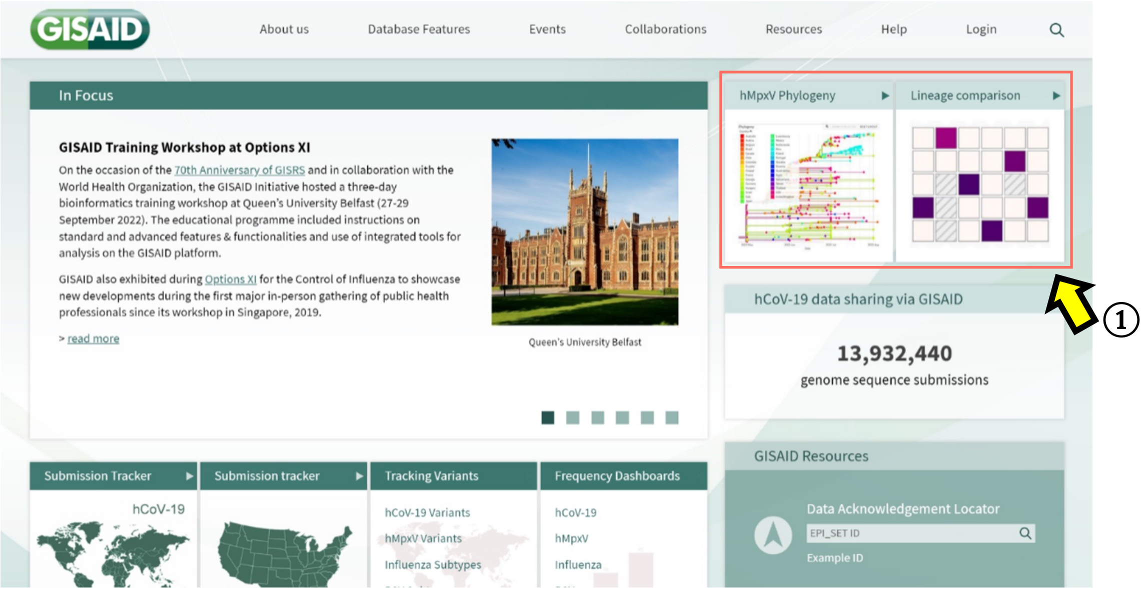

① **GISAID reports current SARS-CoV-2 data through visualization tools without login in**

Main functions: Visualization

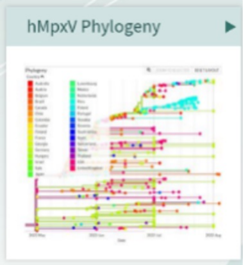

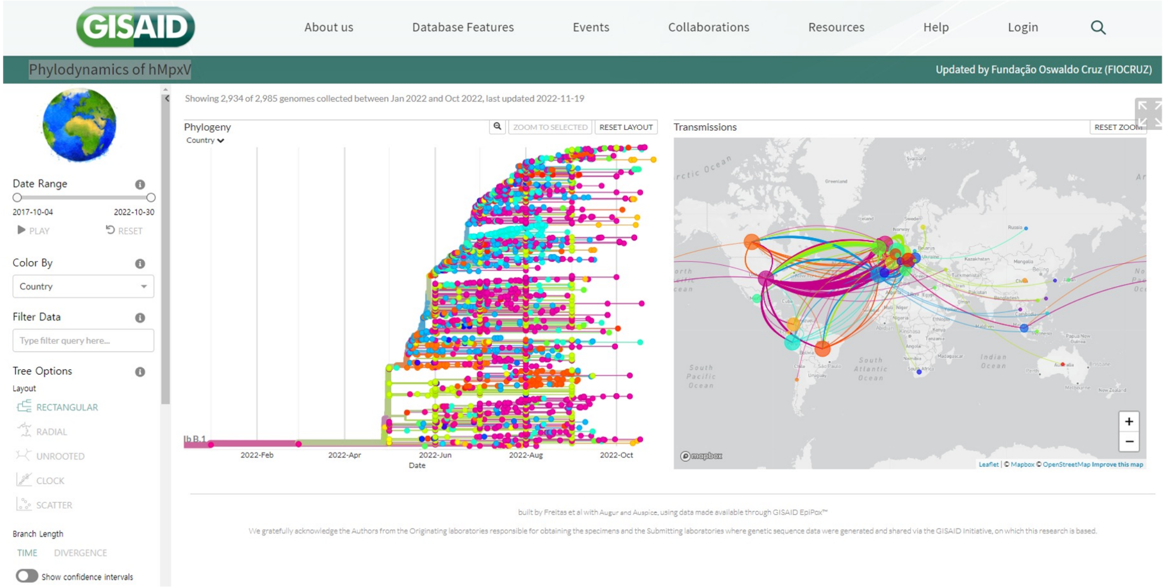

② **Phylodynamics of hMpxV provided**

Main functions: Visualization

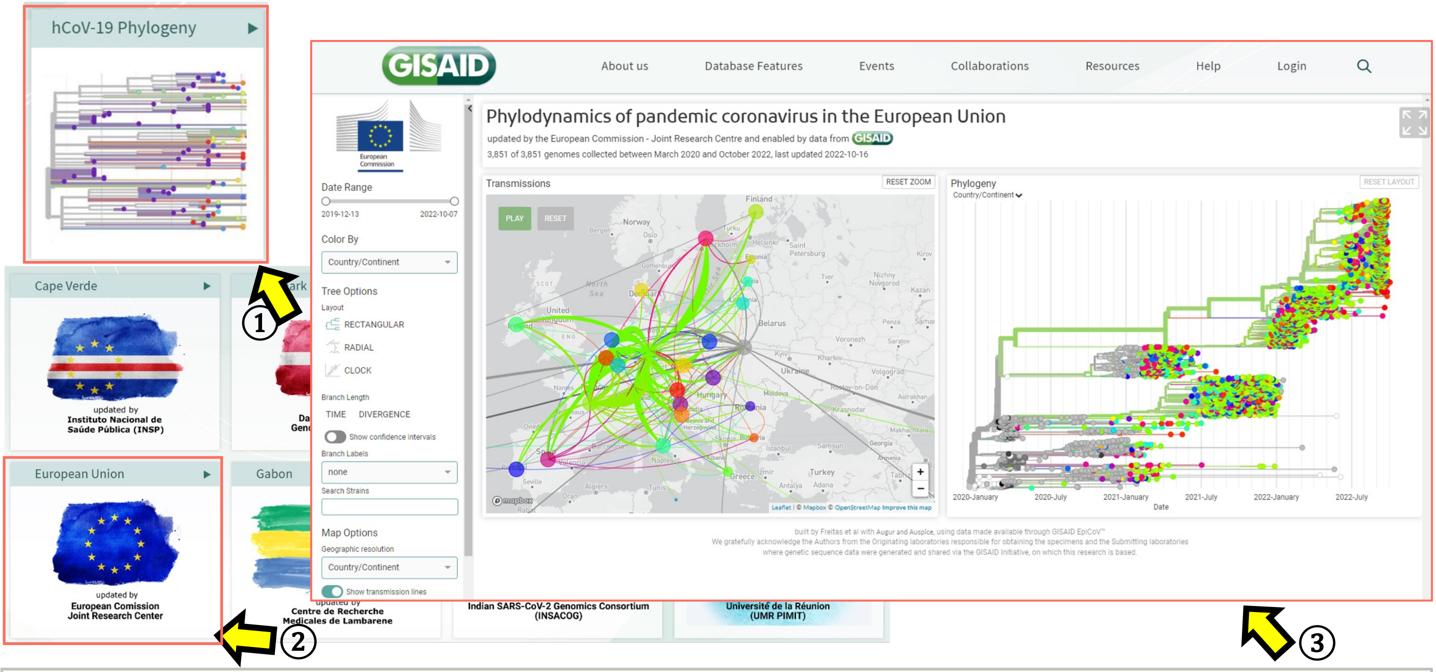

① Clicking on hCoV phylogeny provides Phylodynamics of pandemic coronavirus per country
② Click on country, for example European Union (EU)
③ For EU, data provided for transmission or lineage/clade data per date

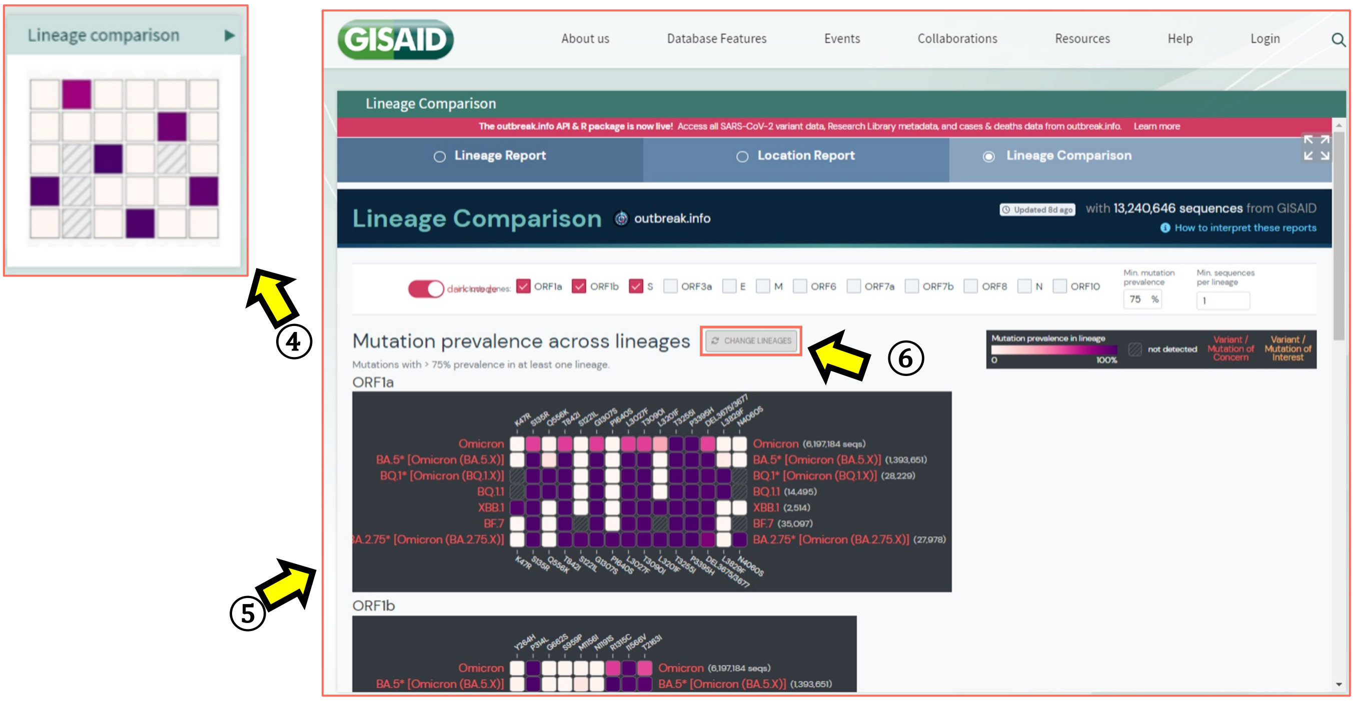

④ Through Lineage comparison, compare mutations between lineages
⑤ SARS-CoV-2 protein structure mutation comparison (Protein structure can be selected)
⑥ Lineage being compared can be selected

Main page

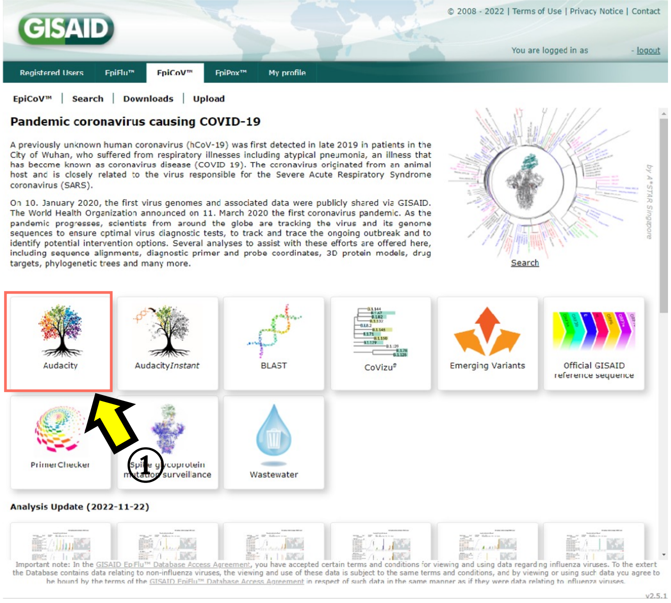

① Audacity provides hCoV-19 global phylogeny through visualization and also provides Newick tree file (Download)

Main functions: Data analysis tool

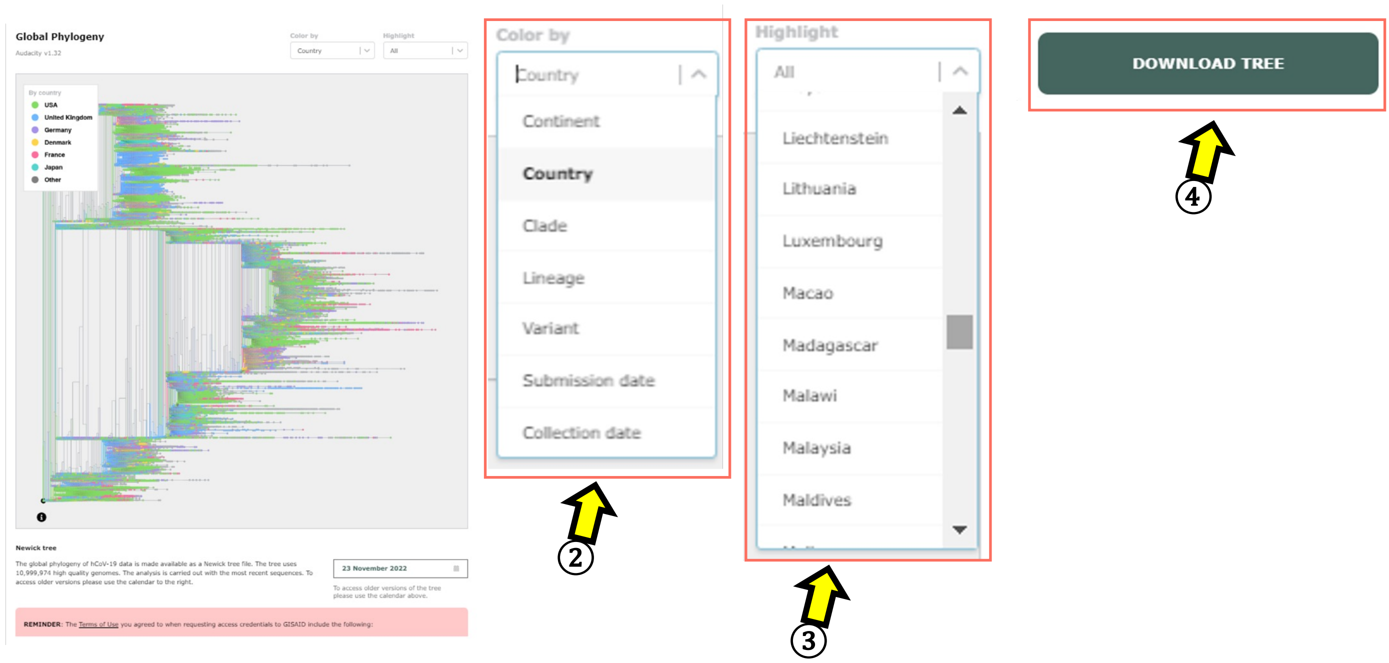

② In Global phylogeny color by options, content, country, clade, lineage, variant, submission date, collection date can be selected
③ In Global phylogeny highlight options, country can be selected
④ Newick tree file uses 10,999,974 high quality genomes, version can be selected

Main page

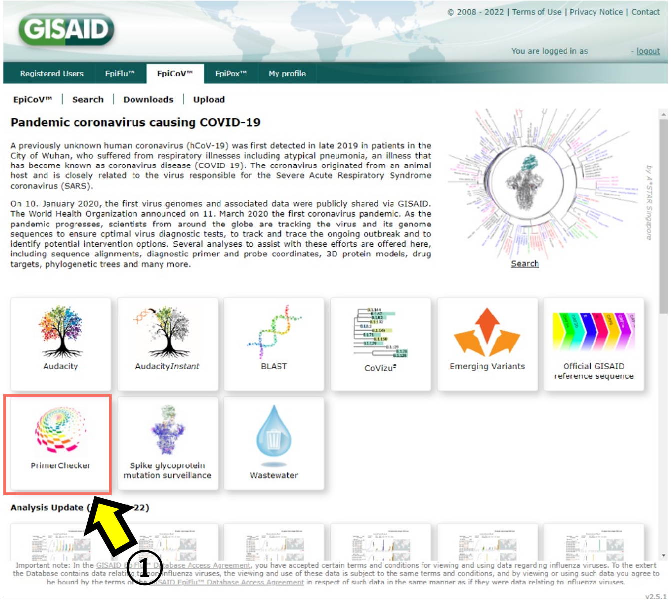

① PrimerChecker is a program that uses "blastn" algorithm to match short sequences against sequences in the GISAID database

Main functions: Data analysis tool

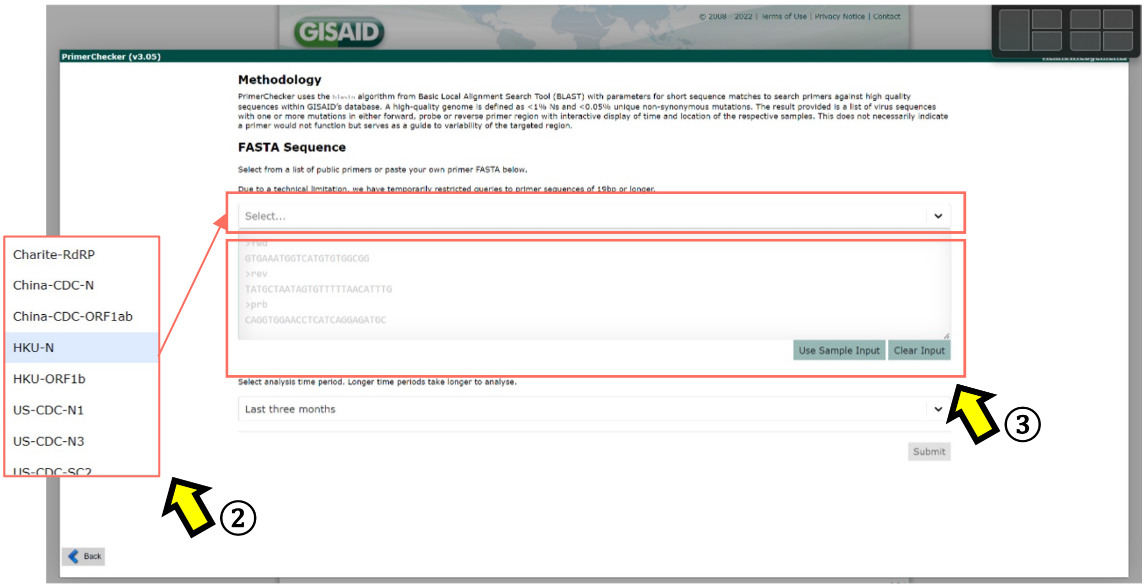

② Possible to select Public primer
③ User can insert their own primers (minimum 19bp)

Main functions: Data analysis tool

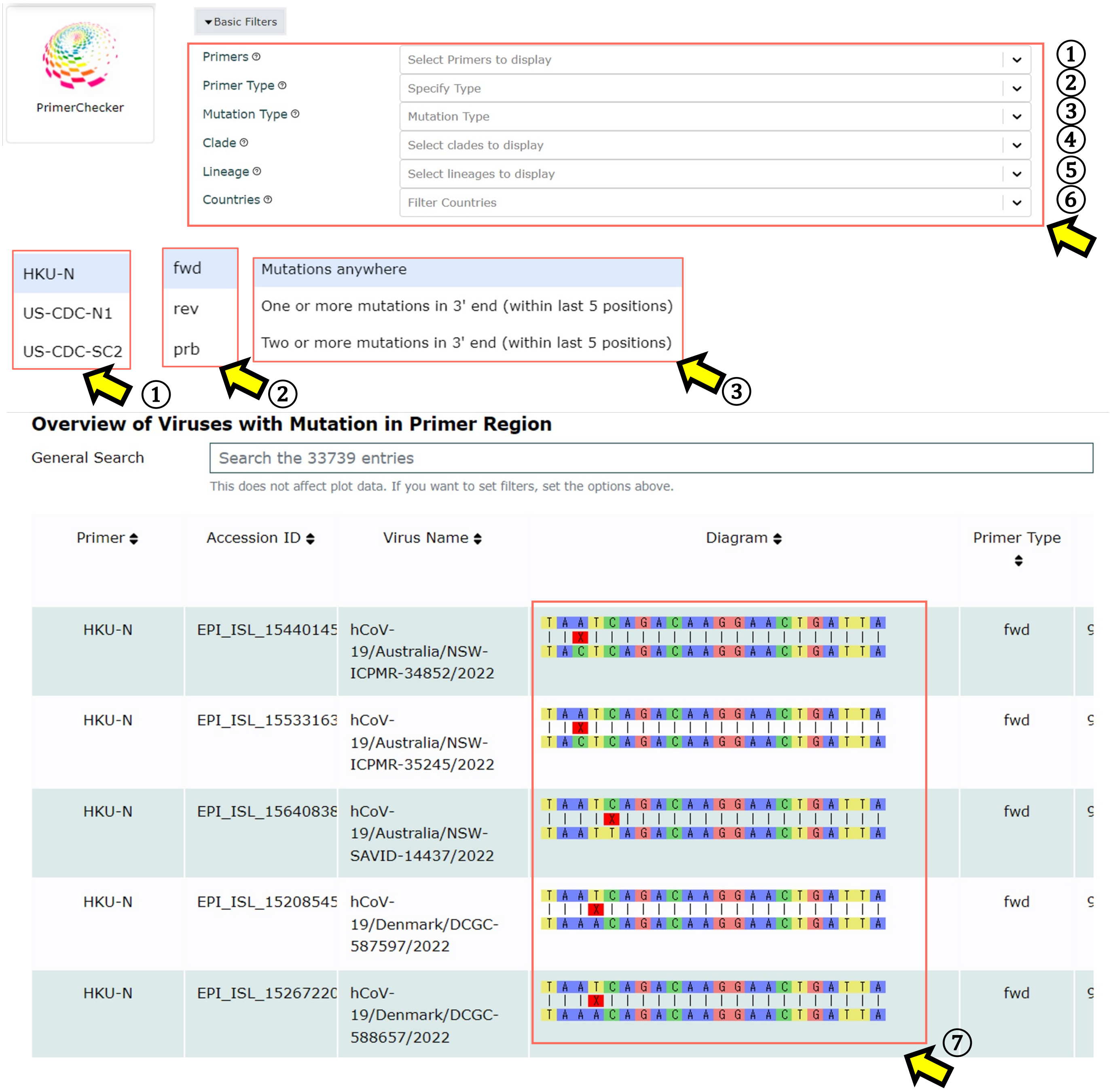

Primer filter possible
① **Select Primer type**
② **Select Primer location**
③ **Select Mutation type**
④ **Select Clade**
⑤ **Select Lineage**
⑥ **Select Countries**
⑦ **Display Mutation region on Primer**

cov-lineages.org

Main page

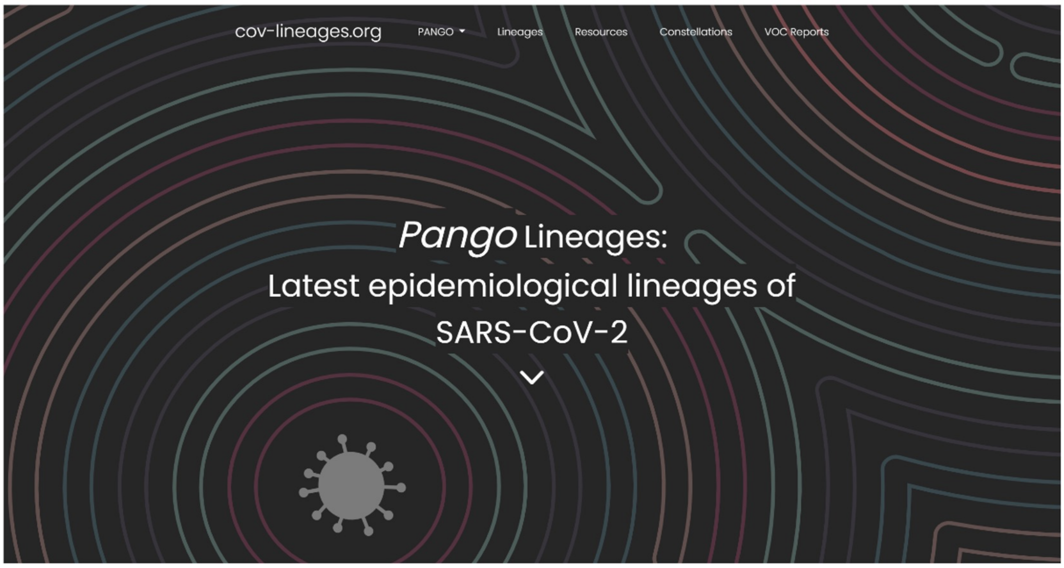

https://cov-lineages.org/

Developed from the Phylogenetic Assignment of Named Global Outbreak Lineages (PANGOLIN), SARS-CoV-2 nomenclature, global transmissions and spread are tracked and visualized. Internally developed tools and software are provided.

Main functions: Clade/variant/lineage

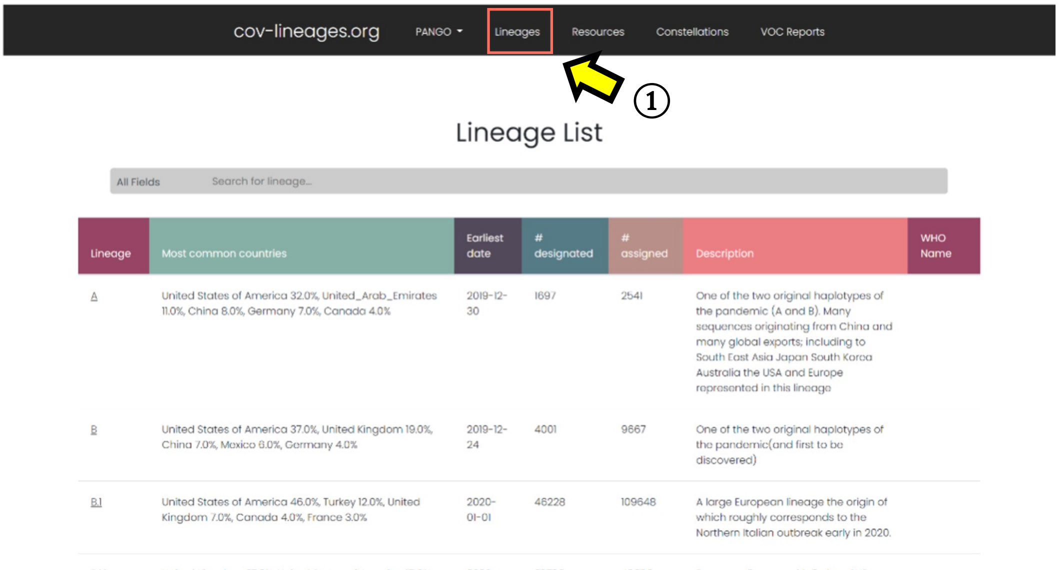

① Clicking on lineage in upper tab shows the lineage list

cov-lineages.org

Main functions: Clade/variant/lineage

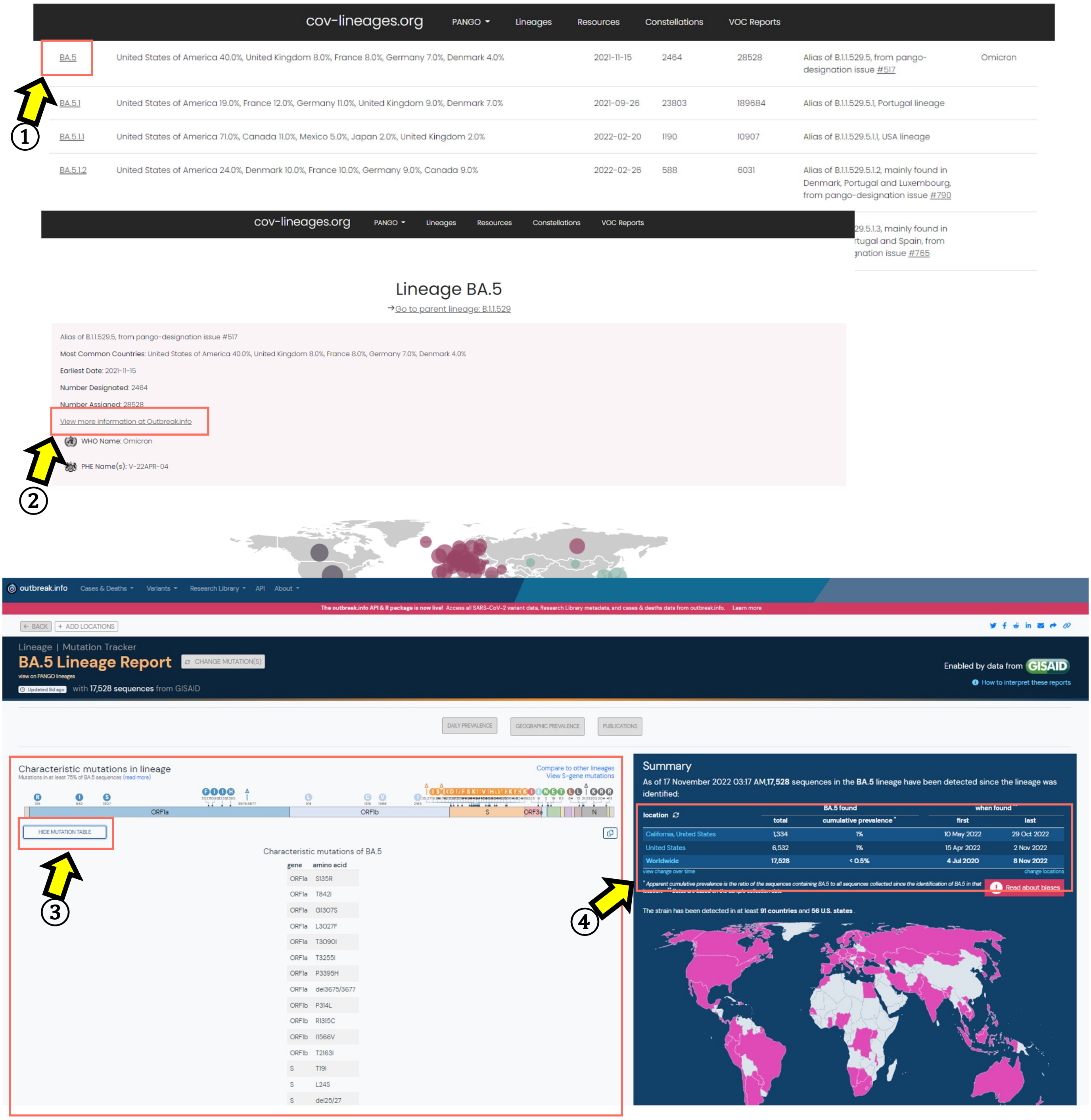

① Clicking on Lineage shows mutation of lineage (To search, input: Window OS: Ctrl+F, MAC OS: command+F)
② Opens information page on Lineage, click on <u>View more information at Outbreak.info</u>
③ SARS-CoV-2 structure and mutation data are visualized. Clicking on View mutation table shows mutation list
④ Visualization of mutation data per country, clicking on location allows changing the selected country

cov-lineages.org

Main functions: Visualization

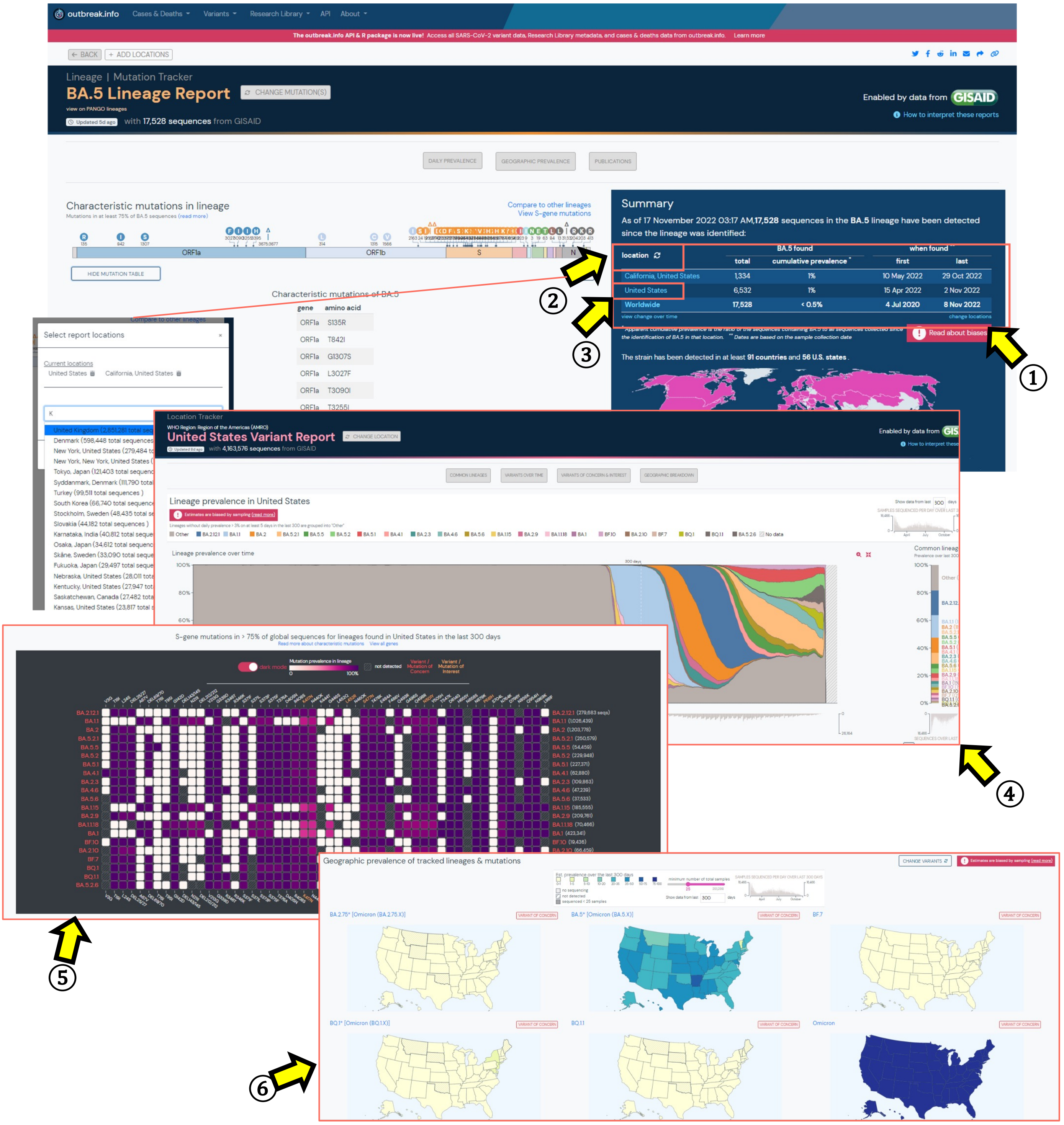

① **Visualization of mutation data per country**
② **Clicking on location allows users to change country**
③ **Select country**
④ **Lineage prevalence over time**
⑤ **S-gene mutations in > 75% of global sequences for lineages**
⑥ **Geographic prevalence of tracked lineages & mutations**

cov-lineages.org

Main functions: Genome browser (sequence)

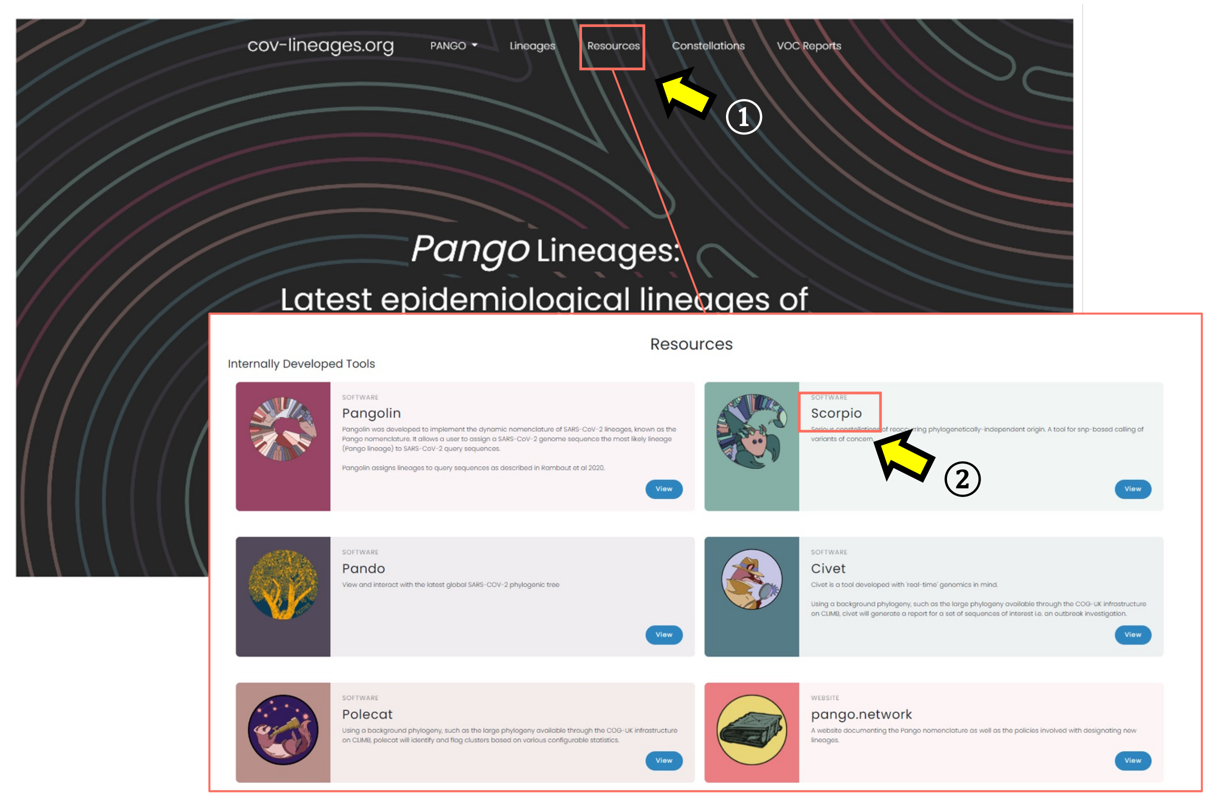

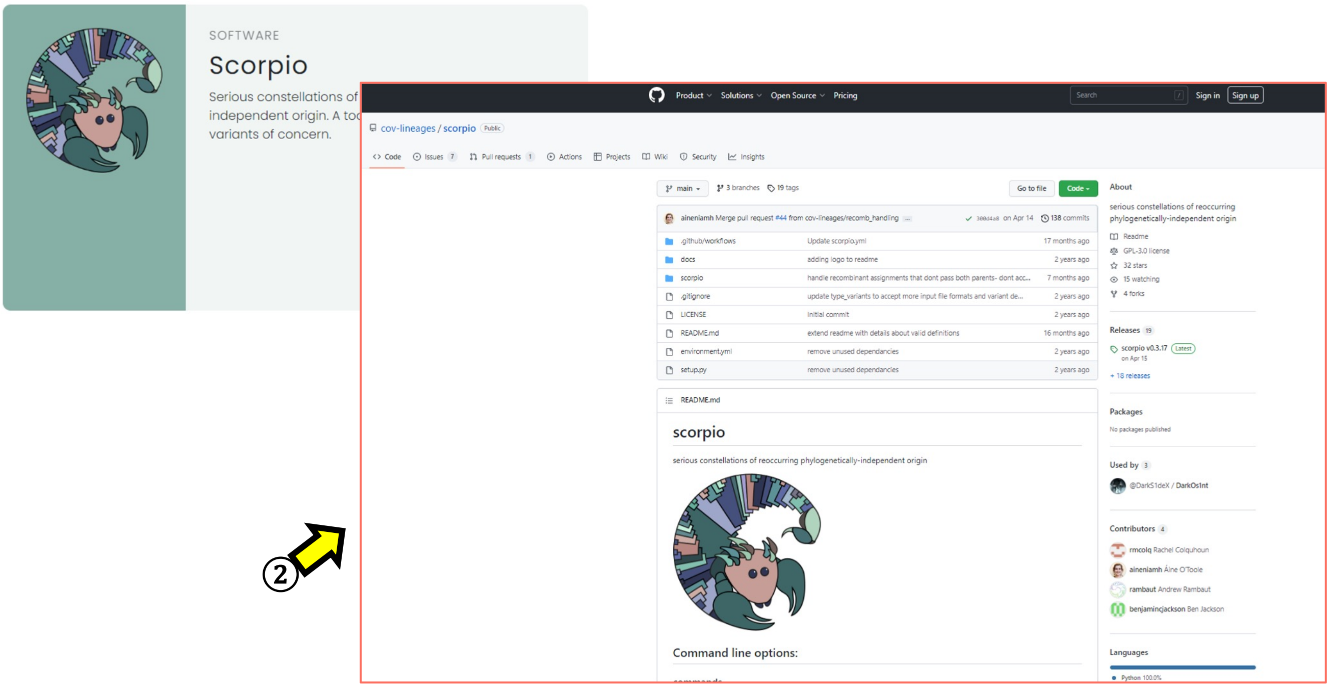

① **Resources provides 6 internally developed softwares**
② **Scorpio: VOC SNP-based calling tools, main software requires installation. Installation and usage guides detailed in github website (https://github.com/cov-lineages/scorpio)**

cov-lineages.org

Main functions: Data analysis tool

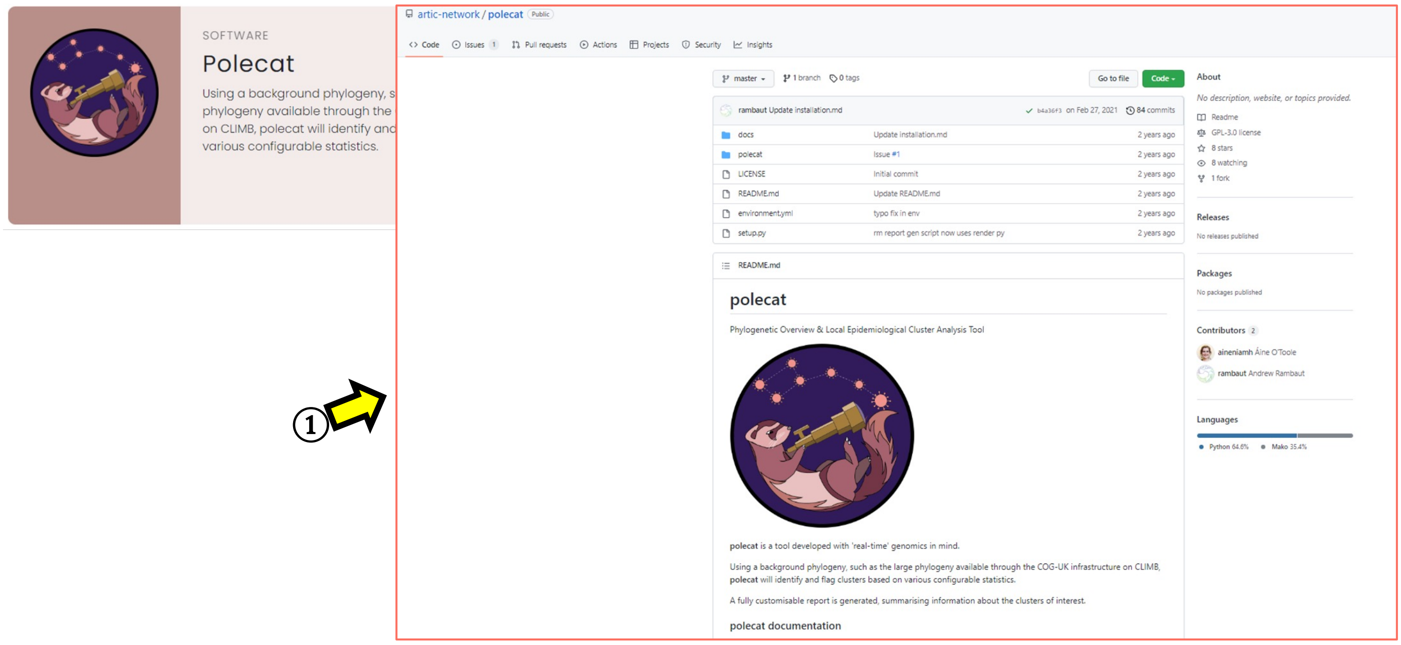

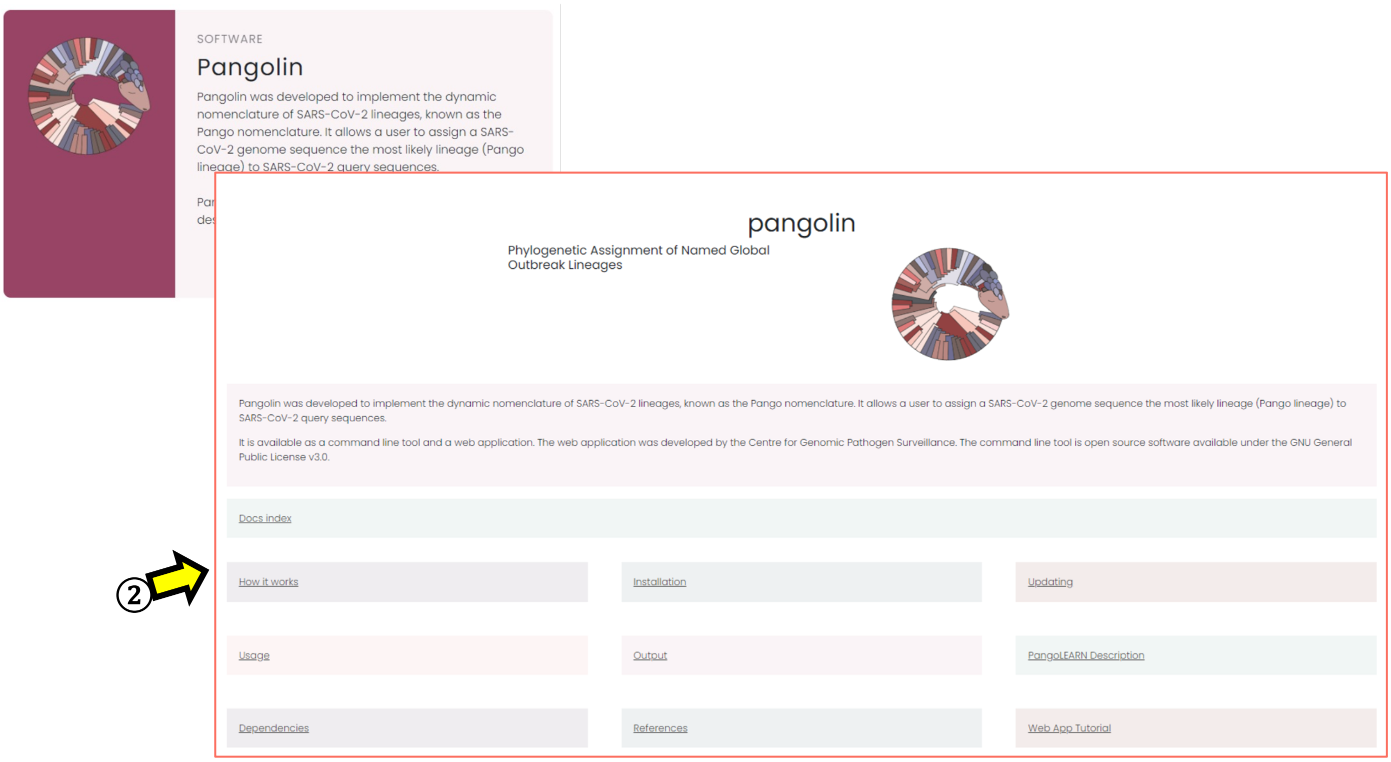

① Polecat: software that through CLIMB COG-UK infrastructure, performs clustering based on mass lineage and lineage occurrence from the same background. Installation and usage detailed in github website (https://github.com/artic-network/polecat)

② Pangolin: software that shows SARS-CoV-2 query sequence lineage that is the most similar to SARS-CoV-2 genome sequence (https://cov-lineages.org/resources/pangolin.html). Installation is through: https://cov-lineages.org/resources/pangolin/installation.html, usage is detailed in this link: https://cov-lineages.org/resources/pangolin/usage.html

cov-lineages.org

Main functions: Data analysis tool

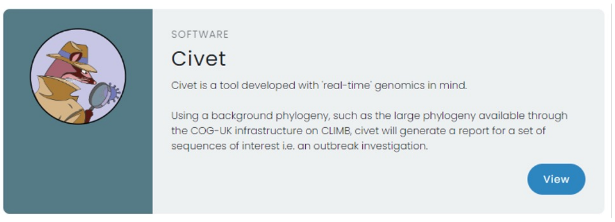

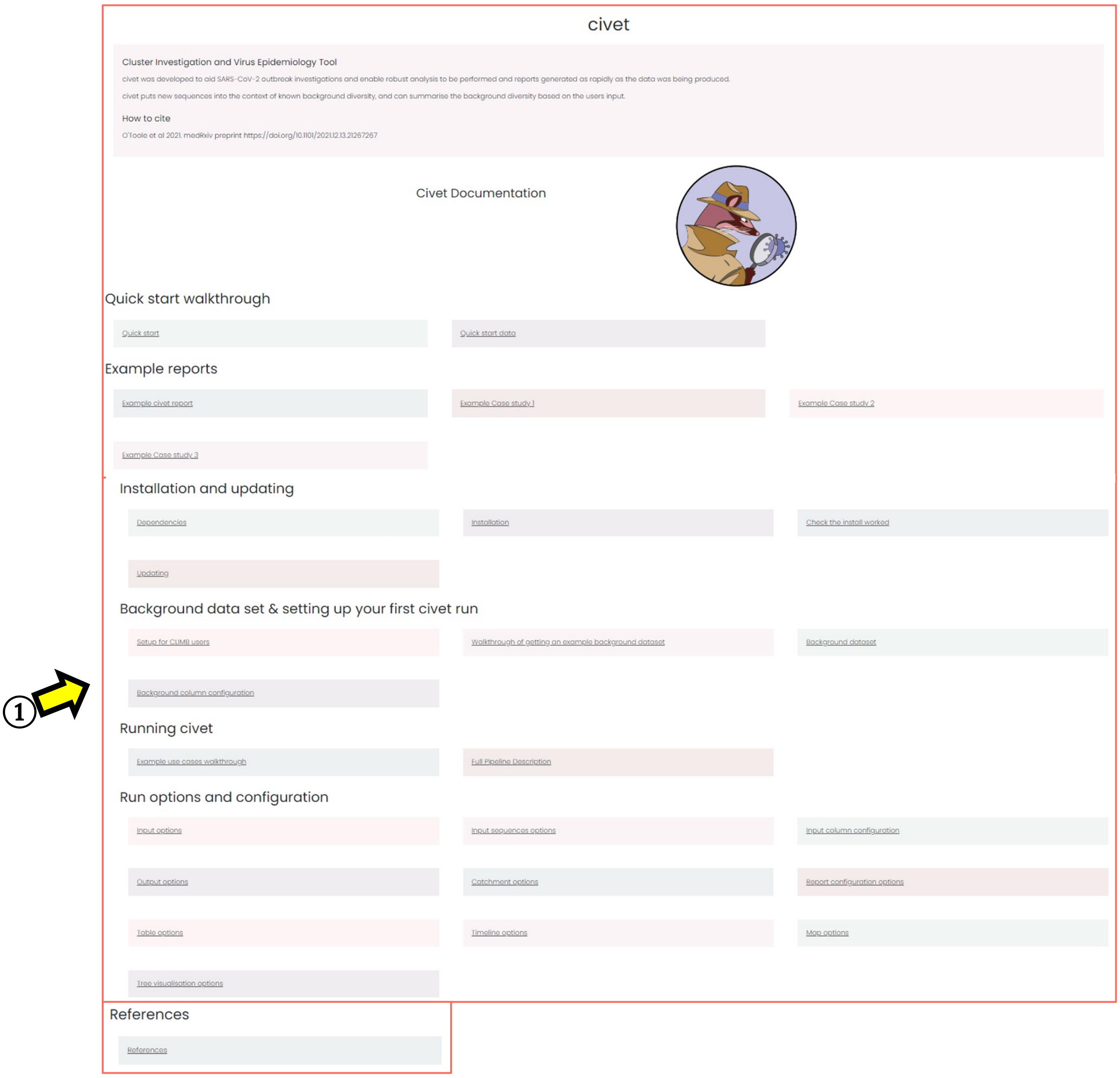

① **Civet: through CLIMB COG-UK infrastructure, phylogeny can be expressed. New sequences are entered in known background diversity content to report based on users. (https://cov-lineages.org/resources/civet.html). Installation is through: https://cov-lineages.org/resources/civet/walkthrough.html usage is detailed in this link: https://cov-lineages.org/resources/pangolin/usage.html**

Main page

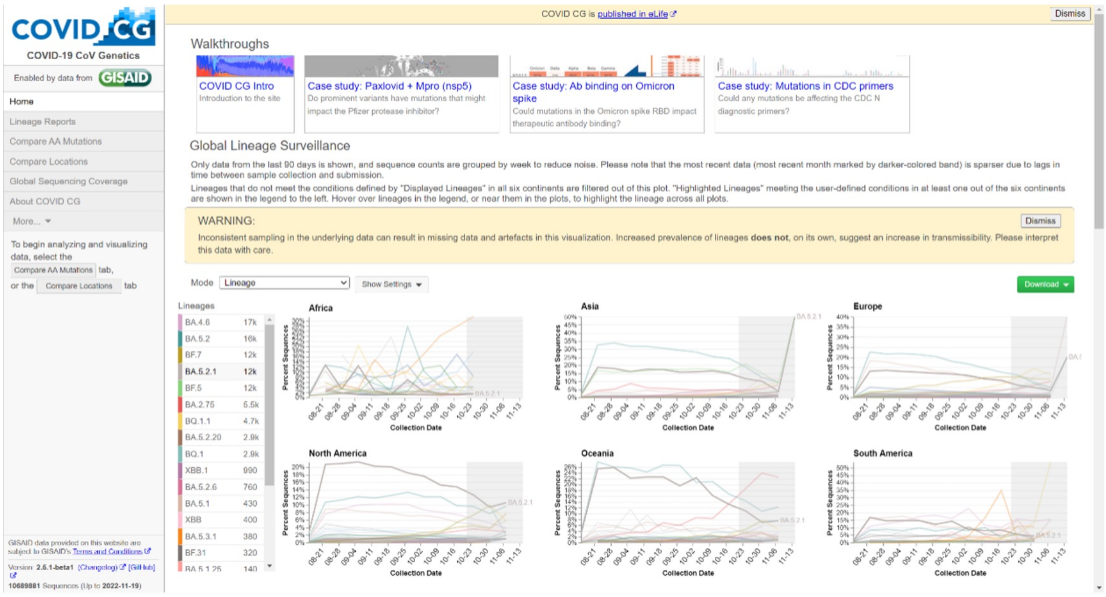

https://covidcg.org/

COVID CG is a variant tracking browser of SARS-CoV-2, virus genomes and metadata analysis, provides data on Informs R&D on diagnostics, vaccines, therapeutics, Tracks global sequencing coverage, etc.

Main functions: Clade/variant/lineage

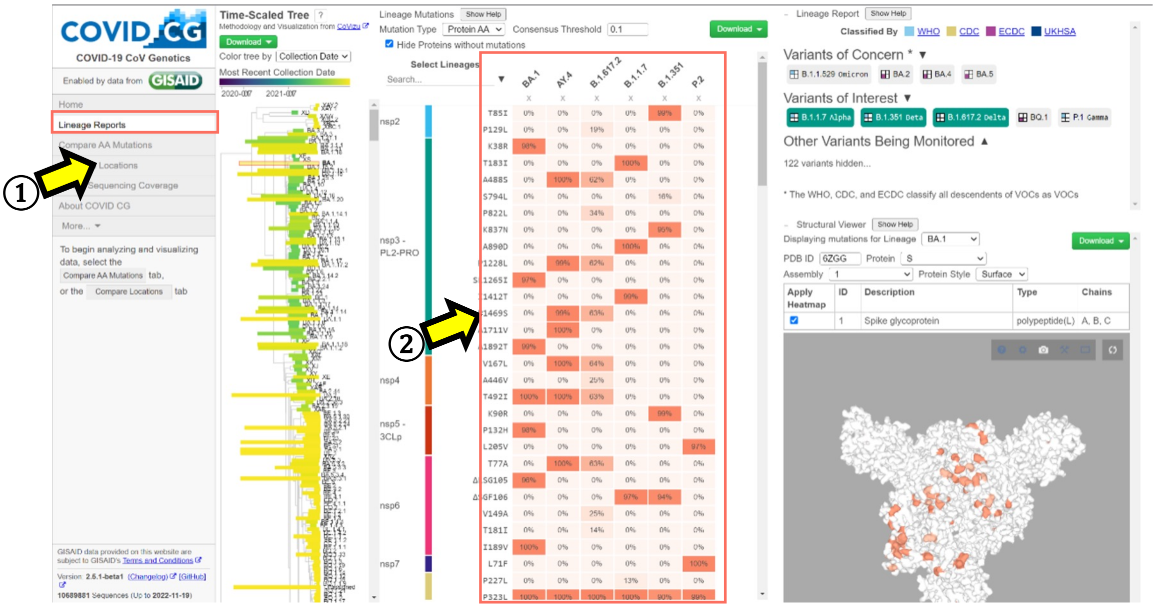

① Click on Lineage Reports
② Data provided for mutation rate of lineages

Main page

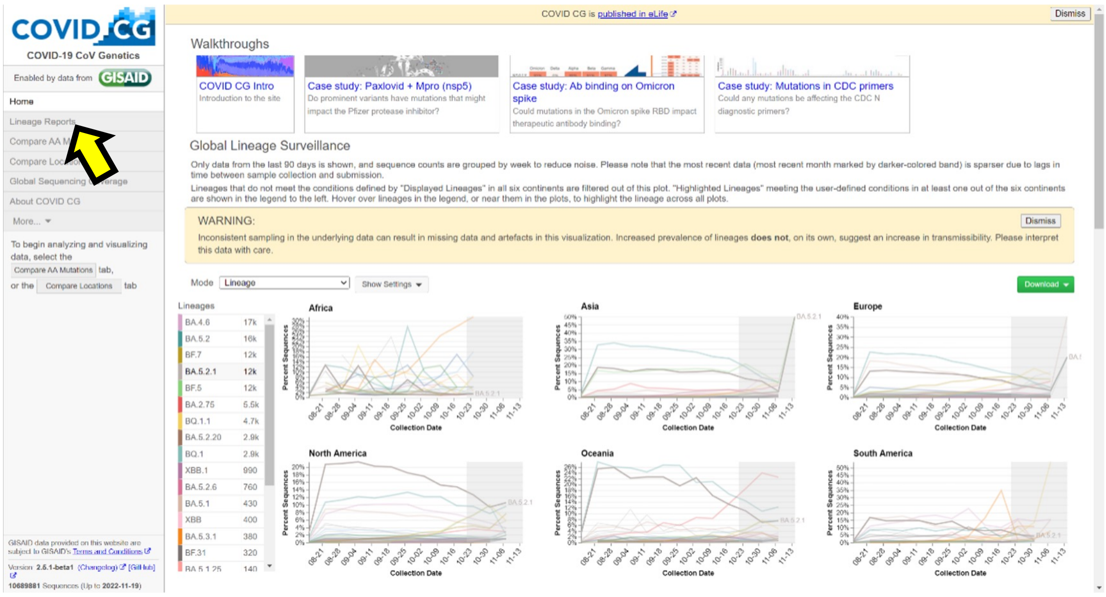

① **Click on Lineage report**

Main functions: Genome browser (sequence)

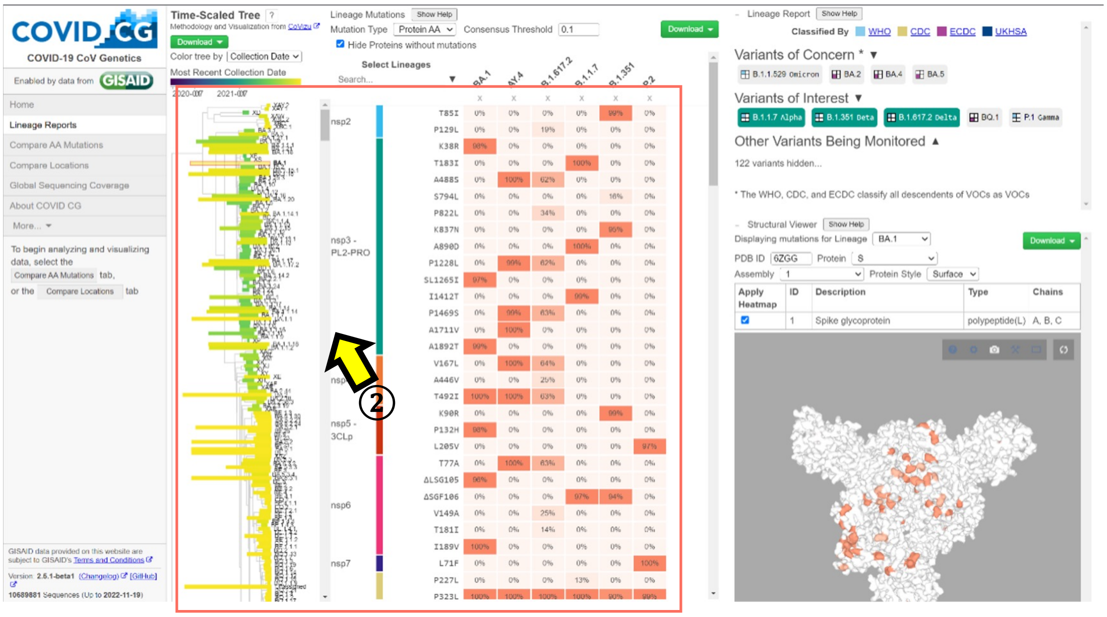

② **SARS-CoV-2 protein structure mutation and lineage data provided**

Main functions: Visualization

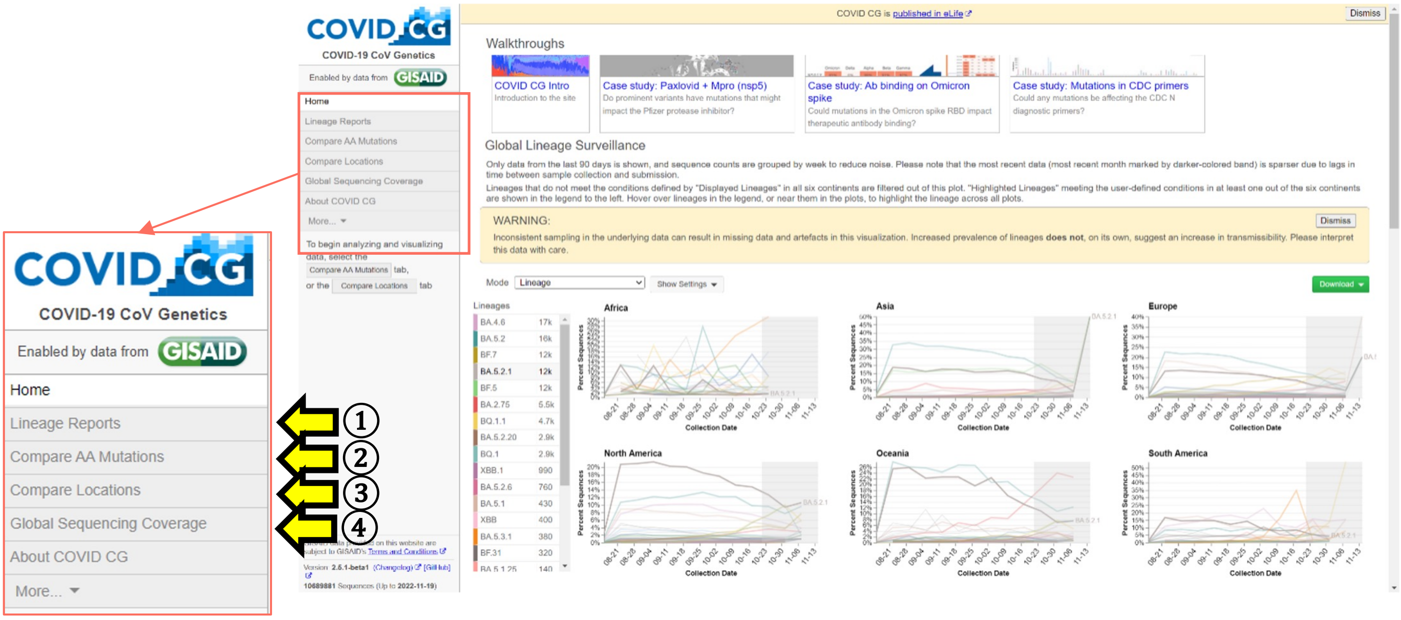

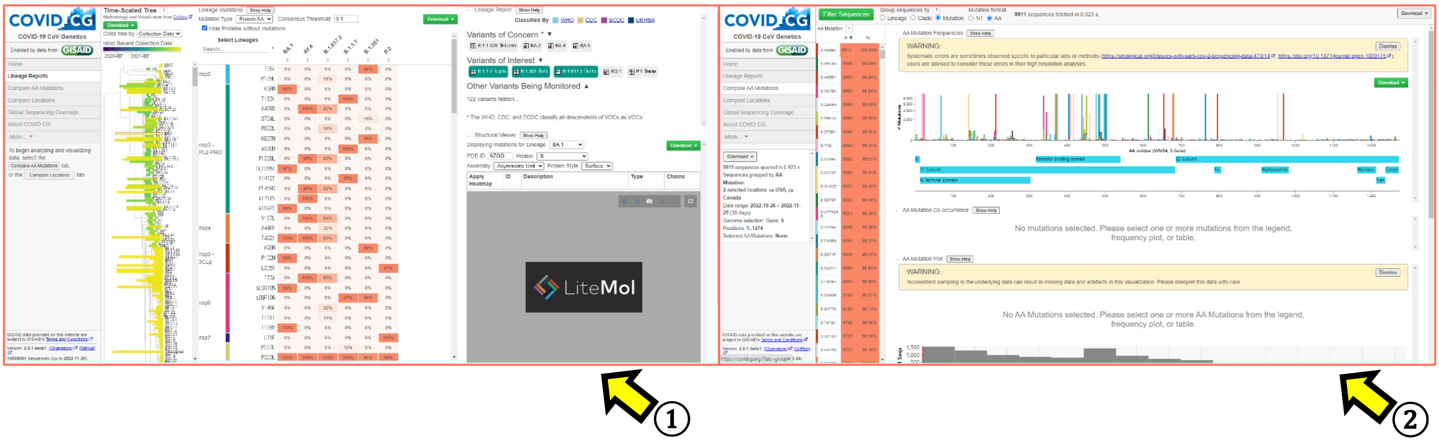

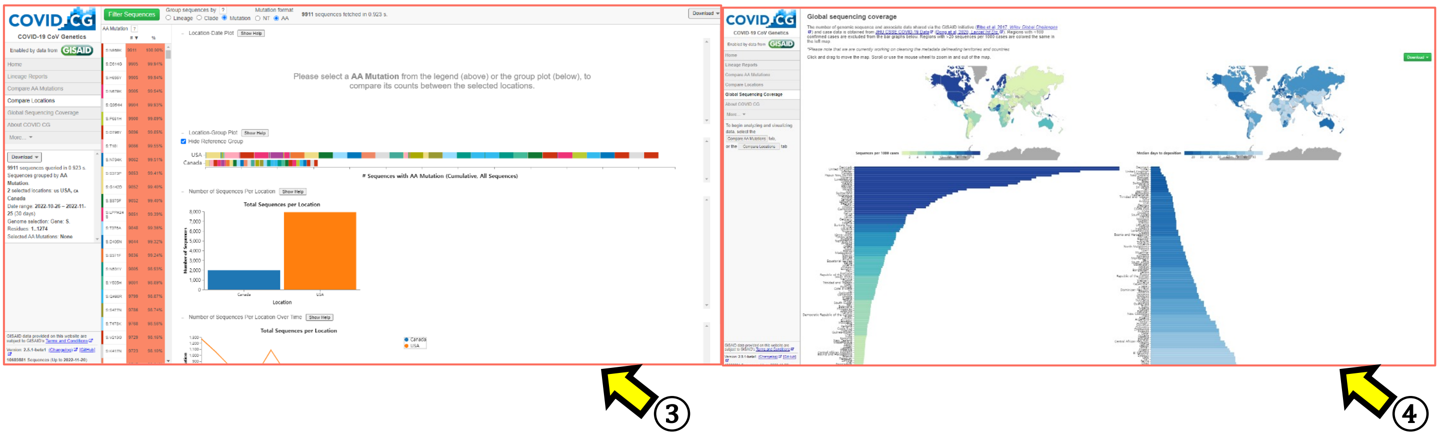

Comprised of Chart, graph, table, etc.
Visualization data provided by clicking on left side menu
① Lineage Report
② Compare AA mutations
③ Compare Locations
④ Global sequencing Coverage

COVID CG
COVID-19 CoV Genetics

Main page

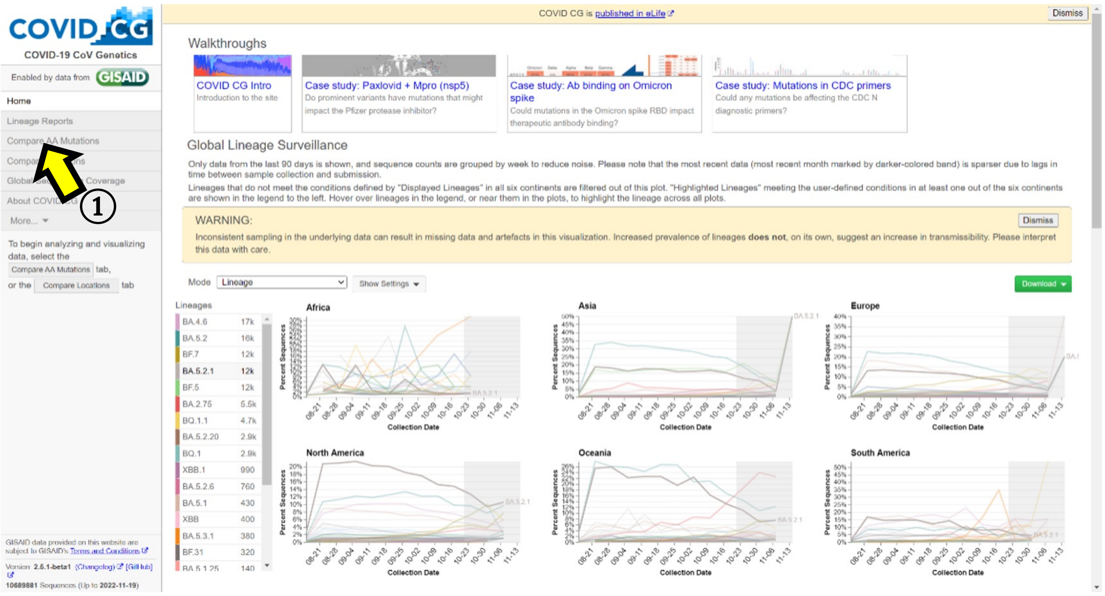

① **Click on Compare AA mutations**

Main functions: Data analysis tool

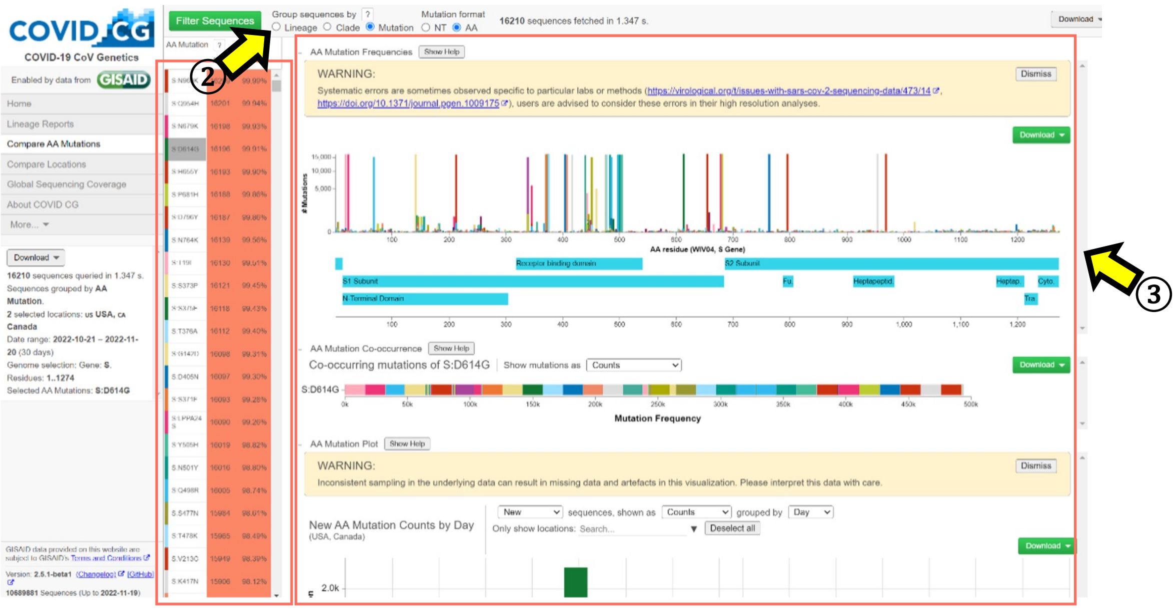

② **Select Lineage, Clade, mutation**
③ **Chart, graph**

COVID-19 Data Portal

Main page

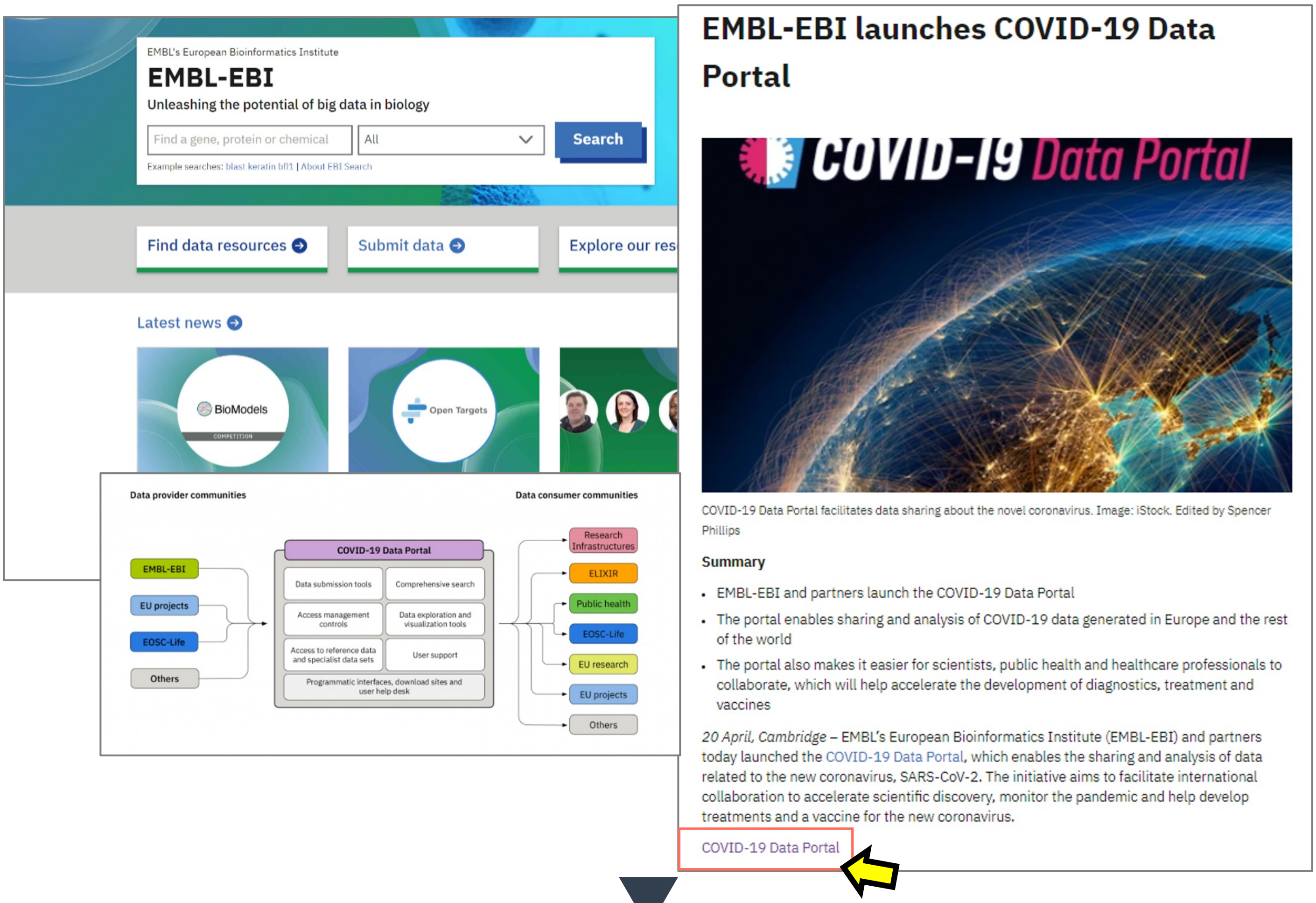

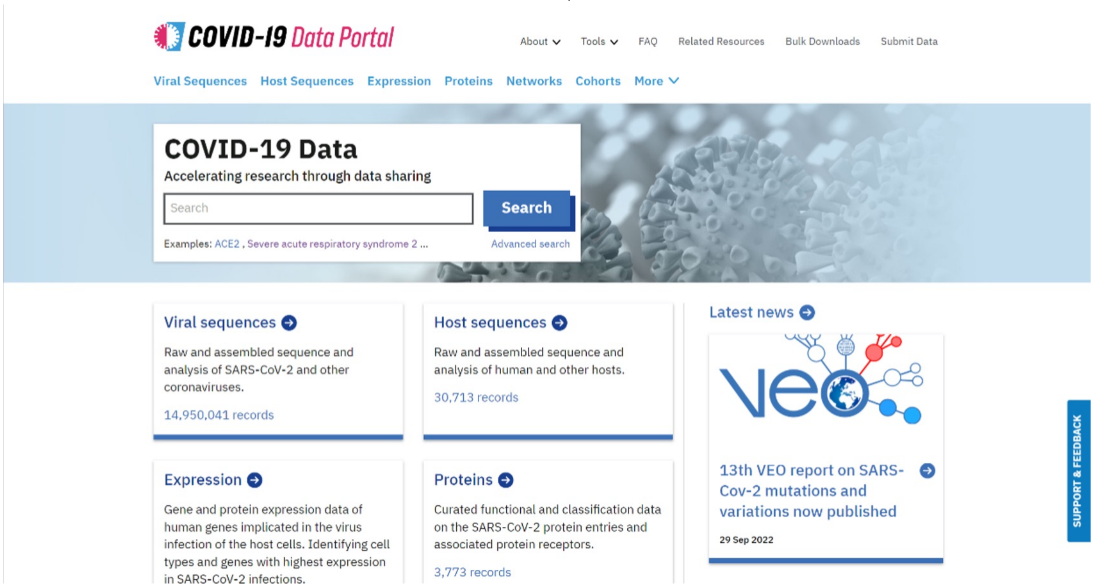

https://www.covid19dataportal.org/

The COVID-19 Data Portal, provided by EMBL-EBI, allows upload, access, and analysis of COVID-19 related reference data and specialist datasets

Main page

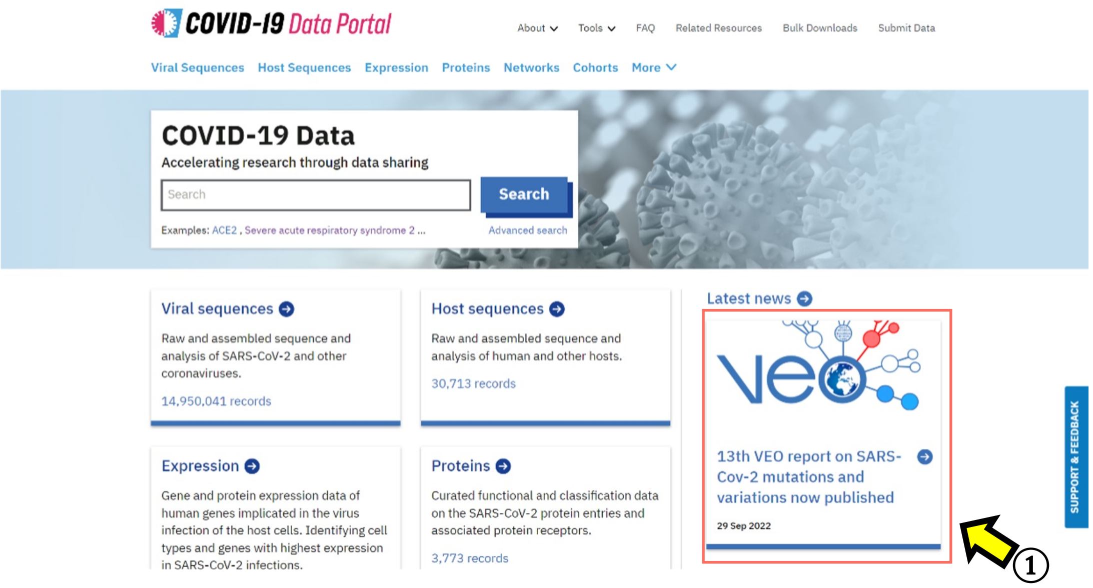

① Click on 13th VEO report on SARS-Cov-2 mutations and variations now published

Main functions: Clade/variant/lineage

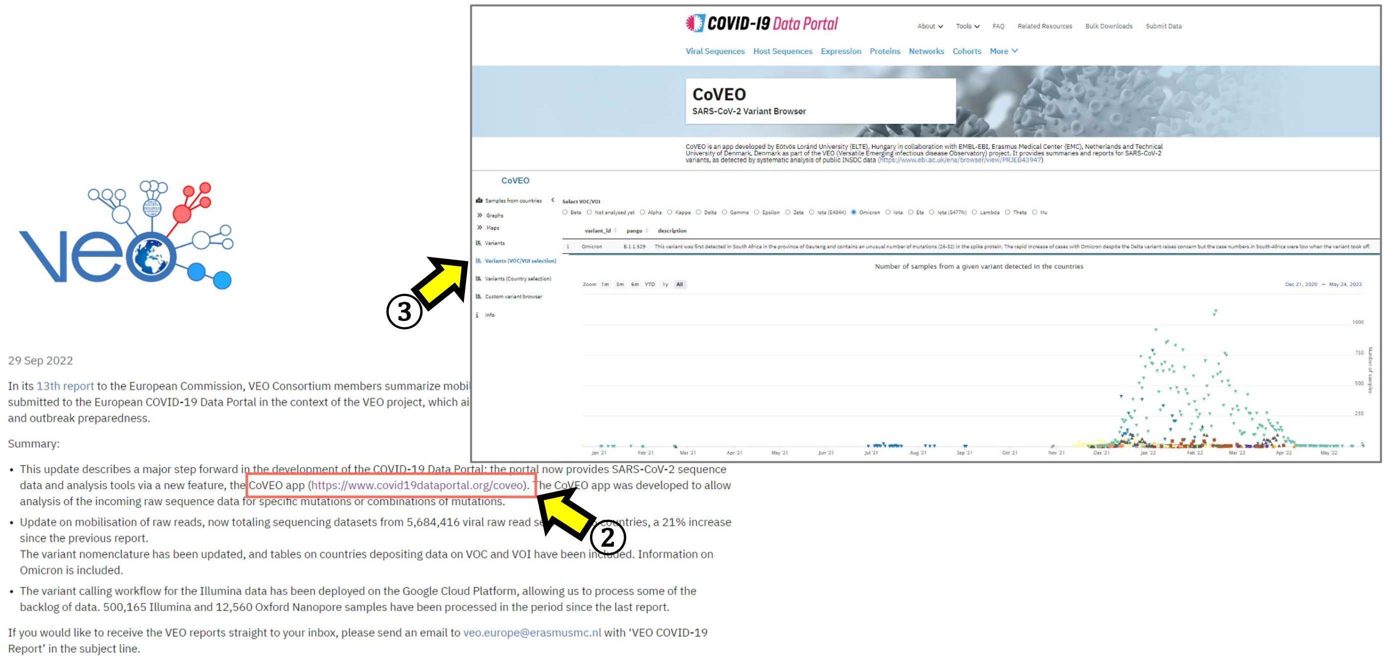

② CoVEO app: an app that can perform analysis for specific mutations or combinations of mutations raw sequence data
③ Variants analysis

COVID-19 Data Portal

Main functions: Genome browser (sequence)

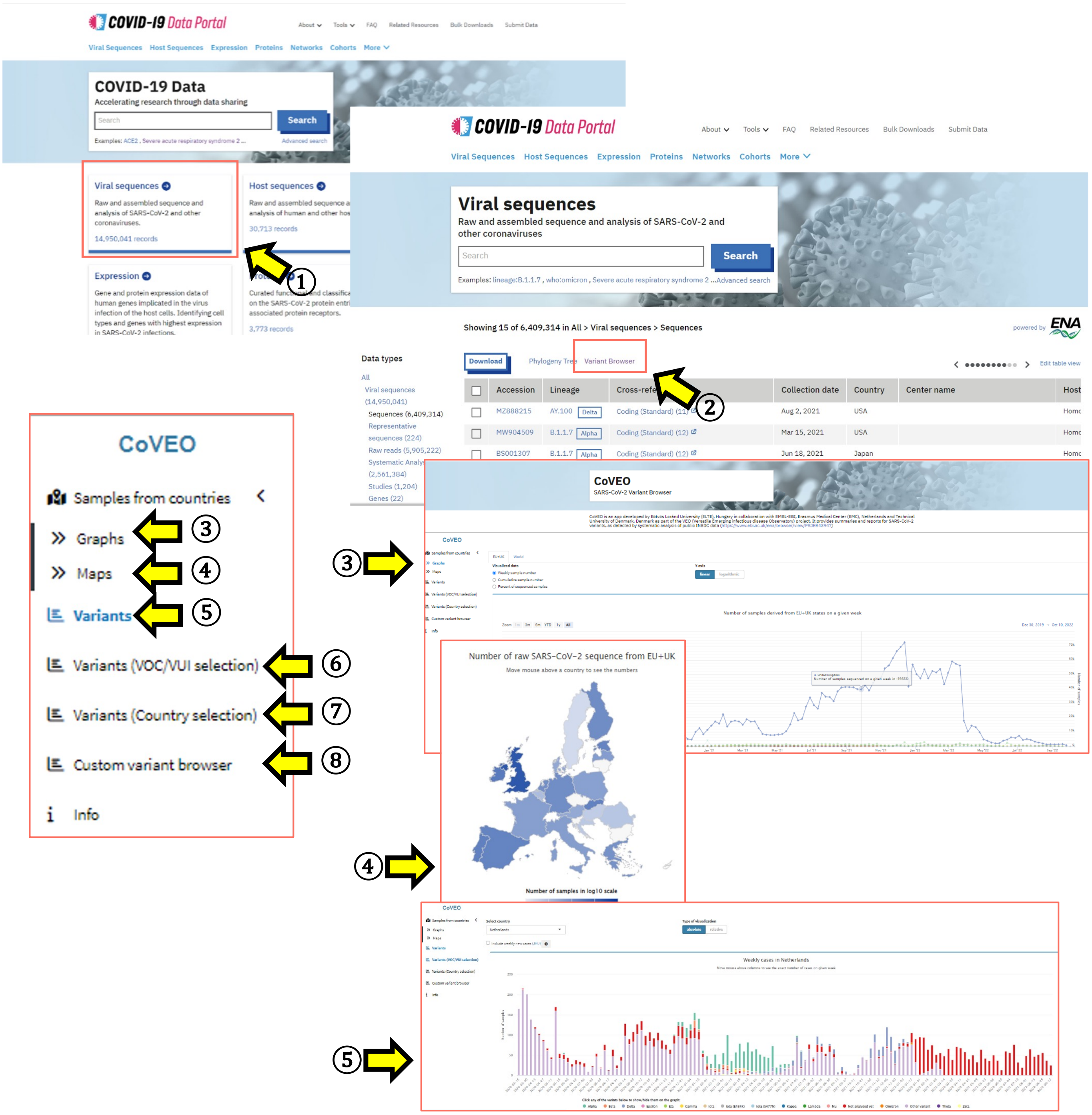

① Click on Viral sequences - Raw and assembled sequence and analysis
② Click on Viral sequences – variant Browser
③ Samples from countries -Graphs
④ Samples from countries -Maps
⑤ Samples from countries -Variants
⑥ Variants (VOC/VUI selection)
⑦ Variants (Country selection)
⑧ Custom variant browser

COVID-19 Data Portal

Main functions: Protein structure

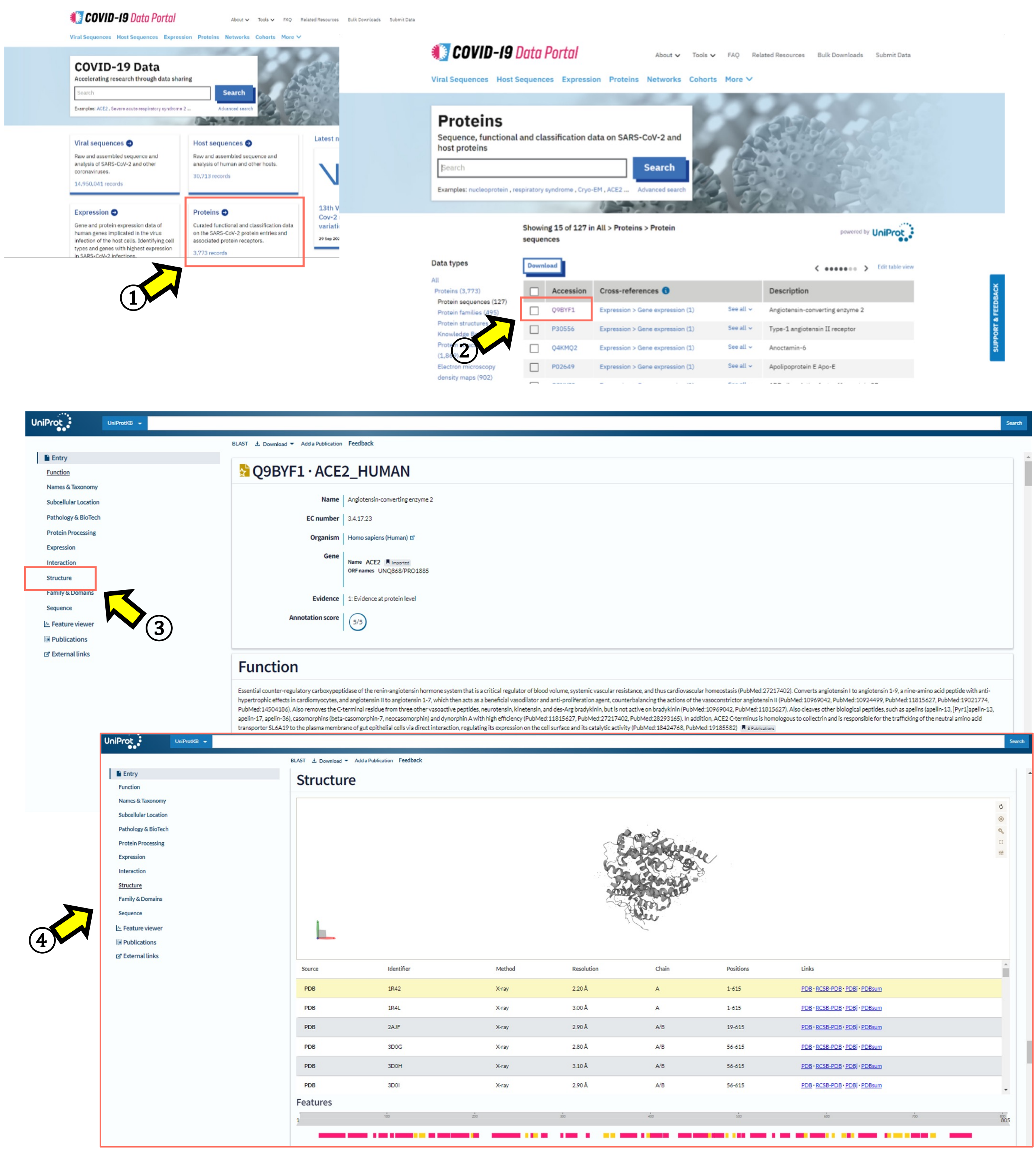

① Click on Protein
② Select Accession – protein ID
③ UniProt associated protein info. provided
④ Menu - Structure click
⑤ PDB information and 3D structure

Main functions: Visualization

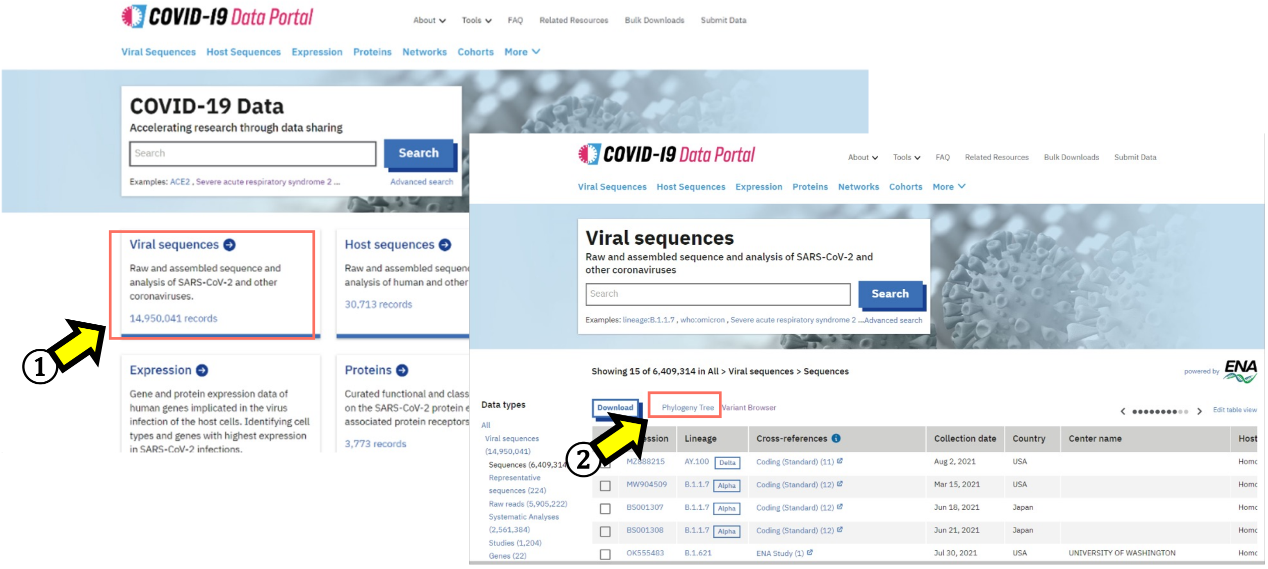

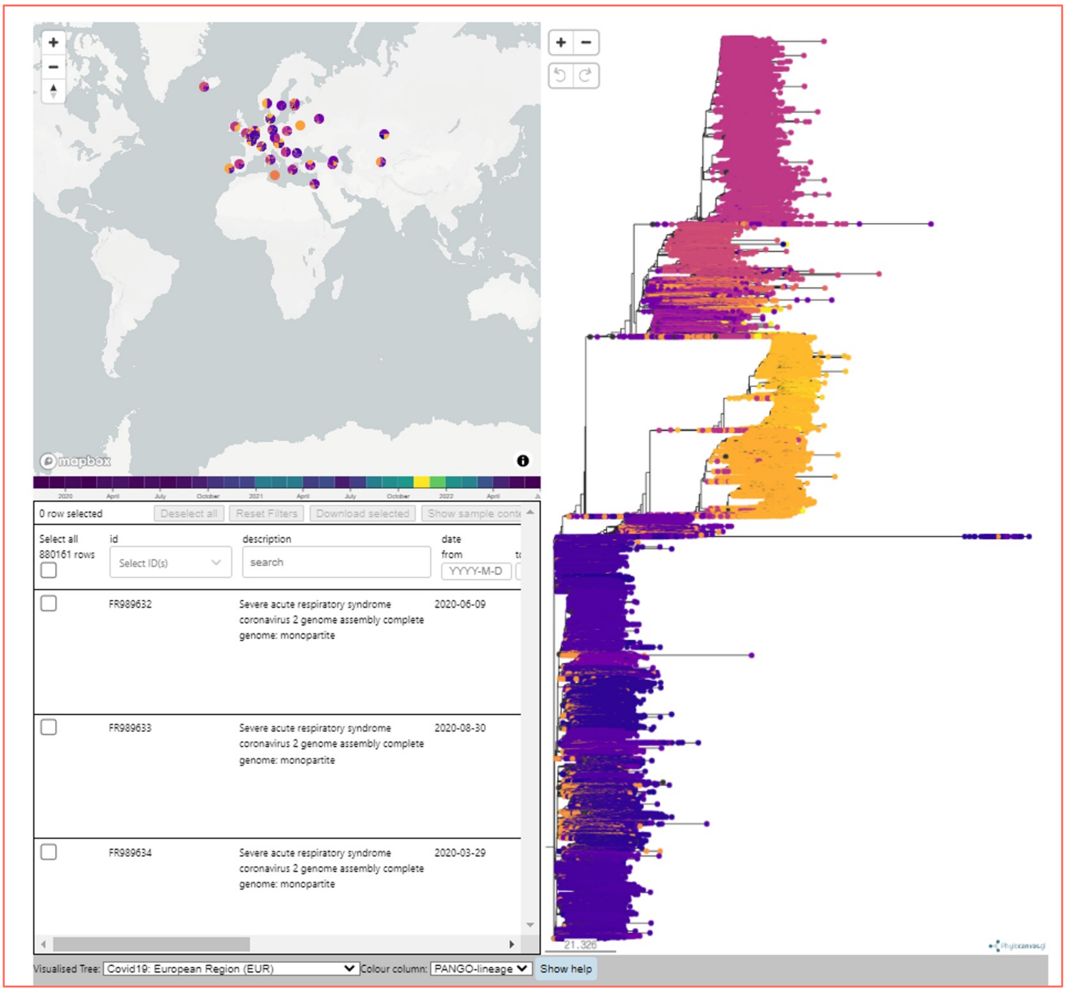

① Click on Viral sequence
② Click on Phylogeny Tree
③ Visualization: Phylogeny Tree, map

COVID-19 Data Portal

Main functions: Literature

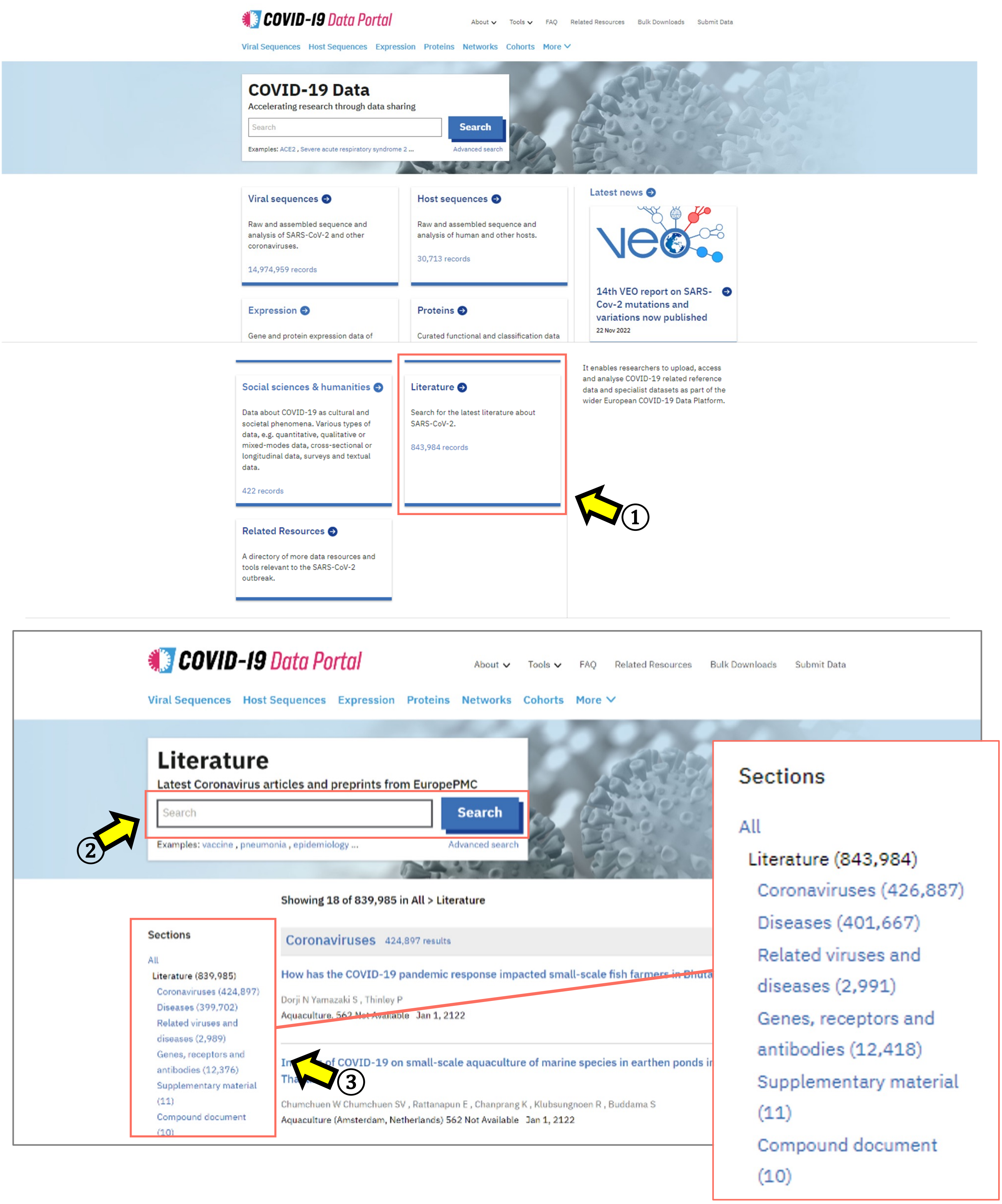

① **Click on Literature**
② **Enter in Search bar**
③ **Categorization into 6 Sections**

Nextstrain

Main functions: Clade/variant/lineage, Genome browser (sequence), Visualization

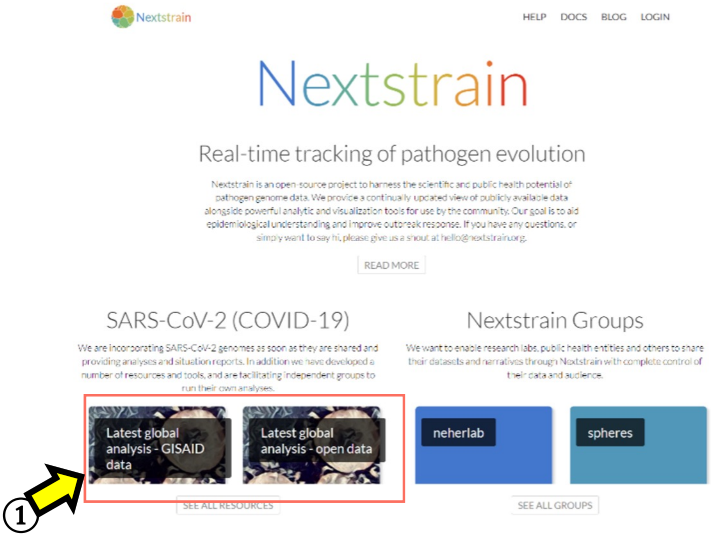

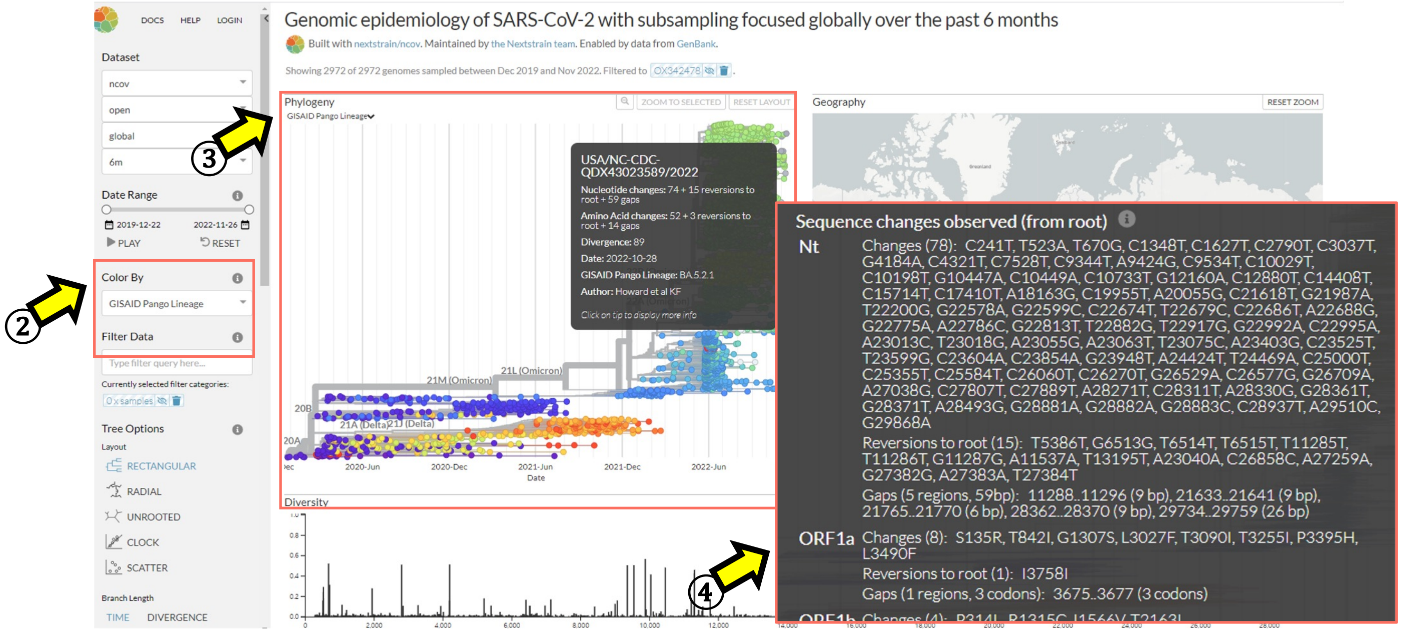

https://nextstrain.org/

Nextstrain visualizes genomic characteristics behind COVID-19 spread and provides open-source tools

① Through Latest global analysis, data on clade, lineage, variant can be obtained
② Through Color by options, select clade , lineage, variant
③ In Phylogeny, click on point (lineage) to obtain lineage data
④ lineage nucleotide levels mutations data and amino acid levels mutations provided.

Nextstrain

Main functions: Clade/variant/lineage, Genome browser (sequence), Visualization

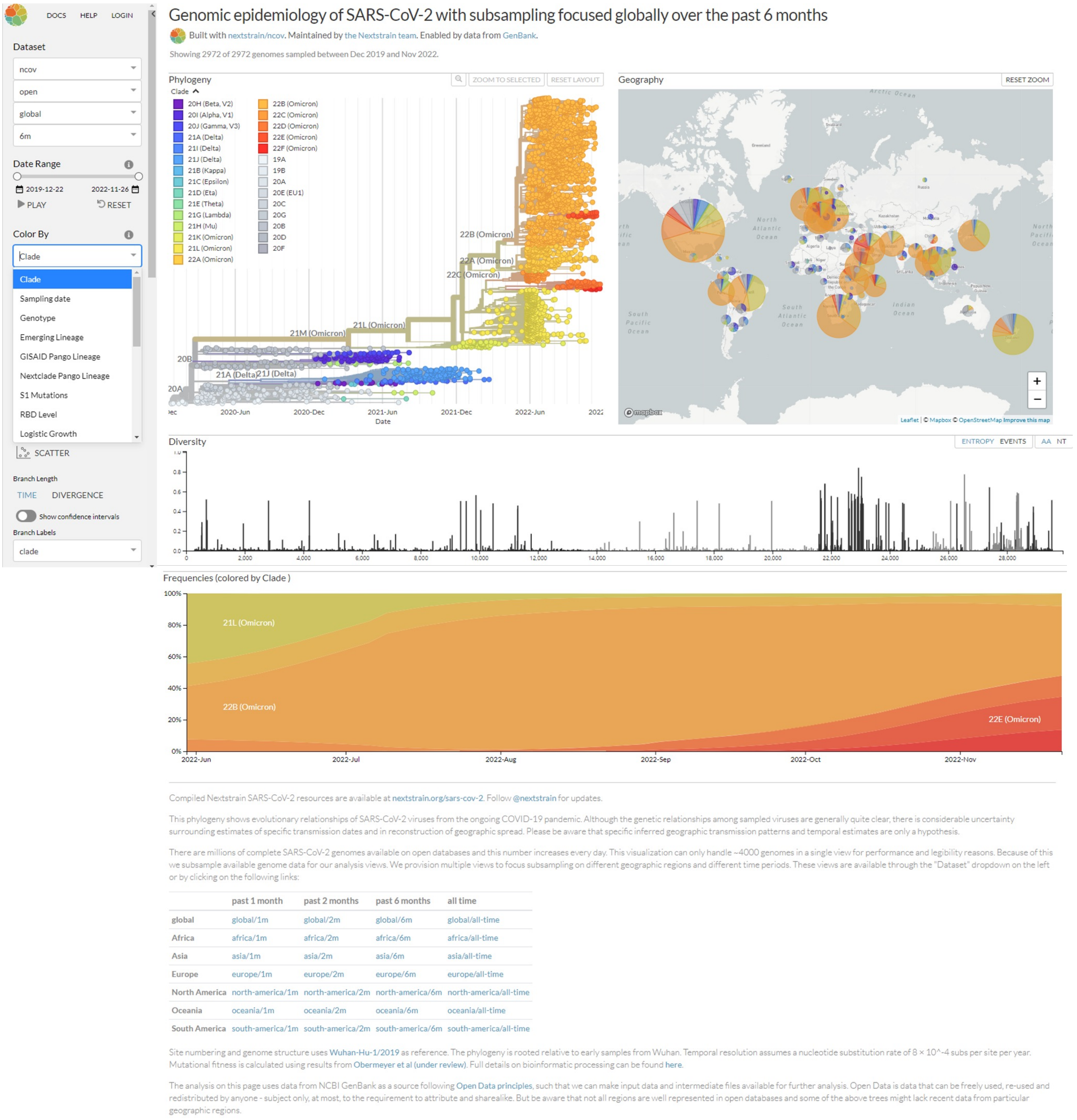

Visualization tool based SARS-CoV-2 genome data provided

Nextstrain

Main functions: Clade/variant/lineage

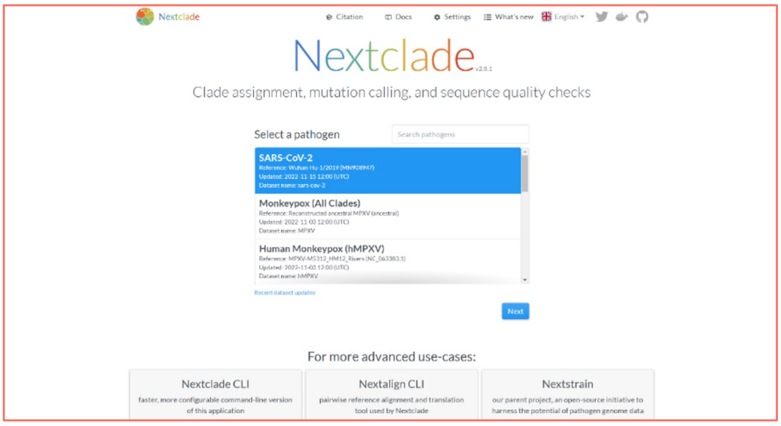

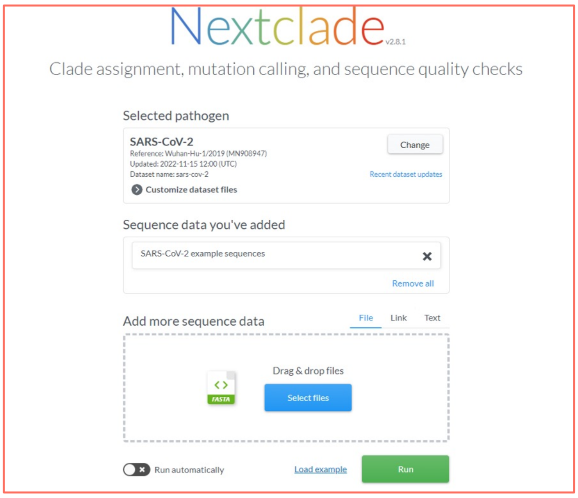

① ②

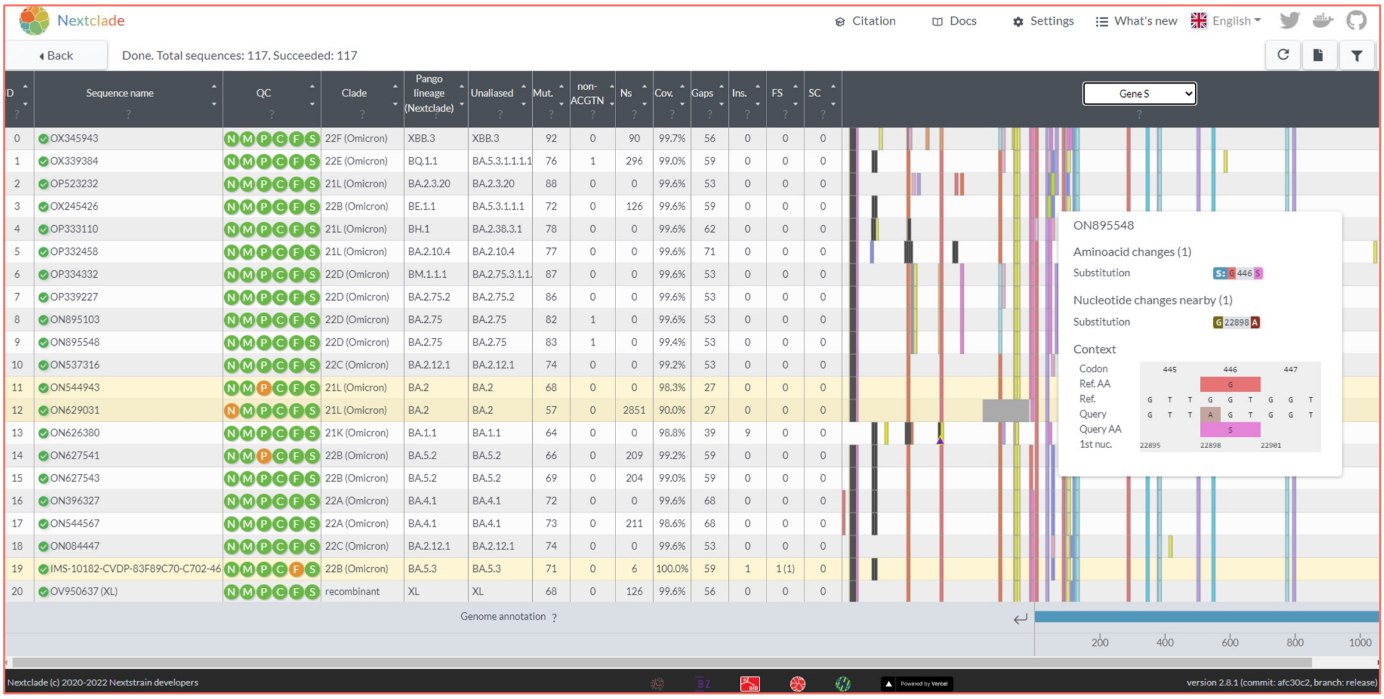

③

https://clades.nextstrain.org/

Nextclade is a tool that compares sequences against reference sequence and reports on clade mutation assignment and sequence quality.
① After selecting select a pathogen, next click
② After linking fasta, text file, or file directory to Provide sequence data, Run.
③ seq. name, calde, mutation, gene position, etc data and visualization provided

Nextstrain

Main functions: Protein structure

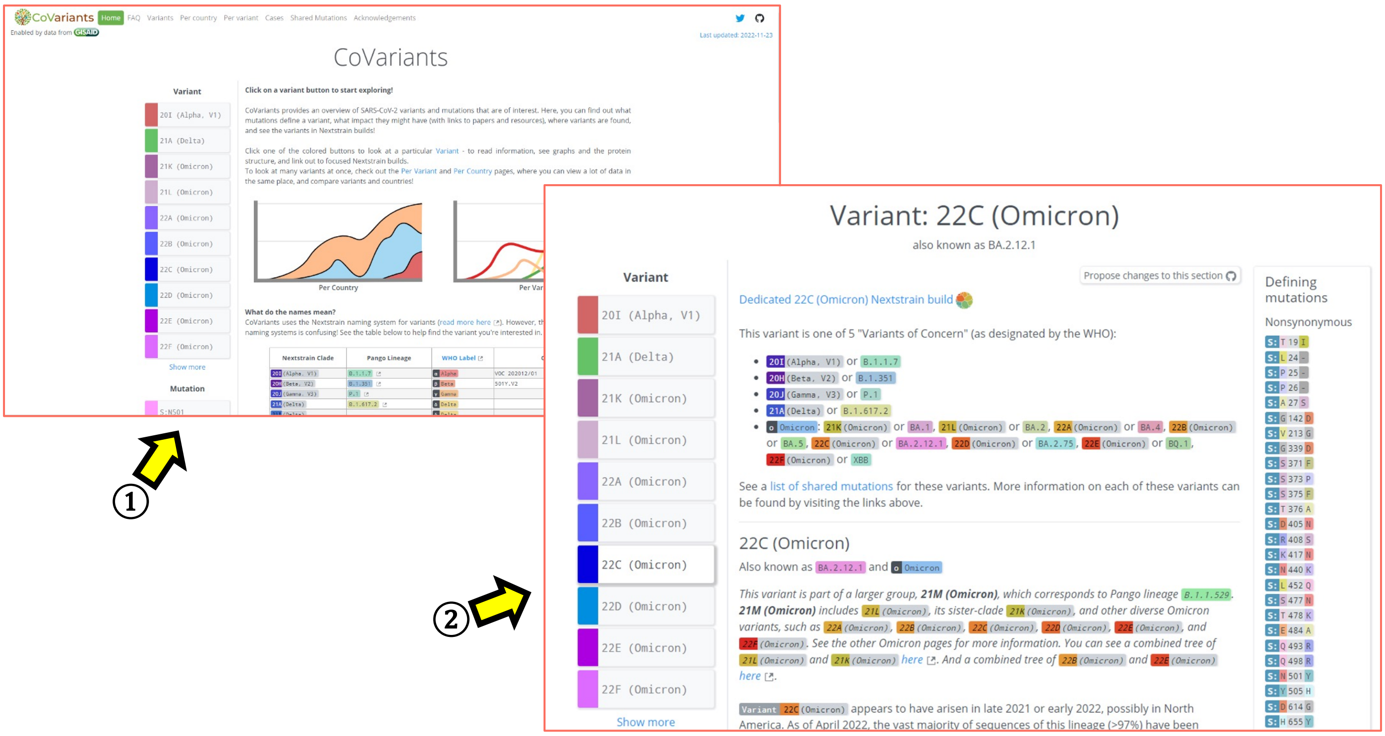

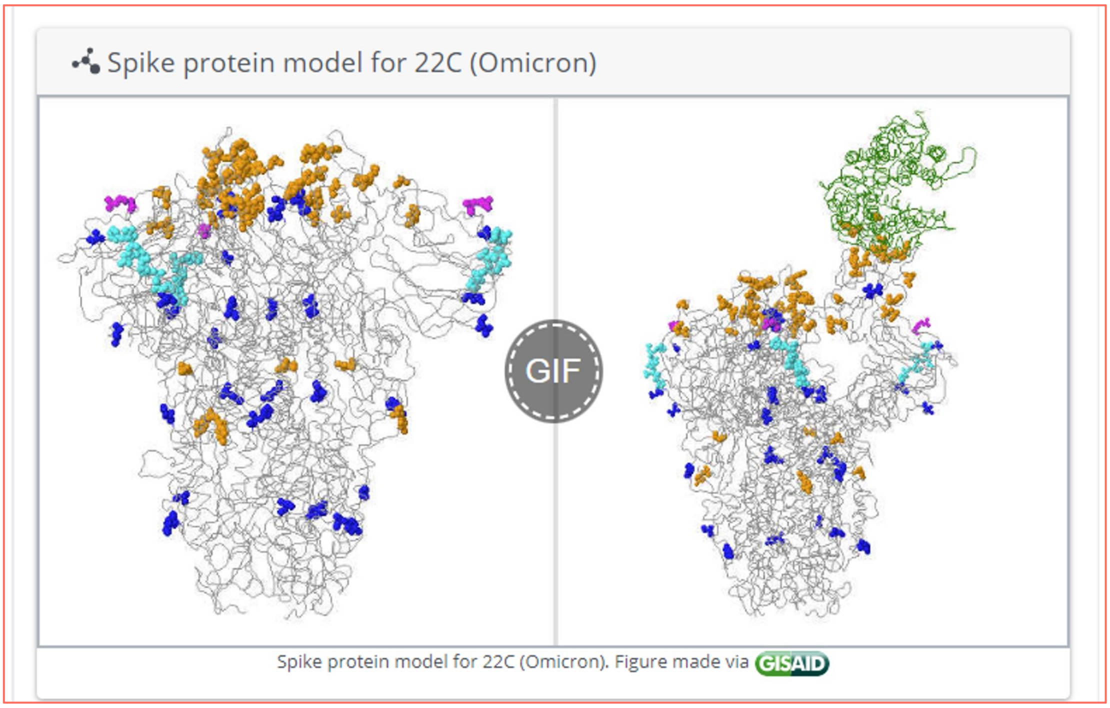

https://covariants.org/

Nextclade provides SARS-CoV-2 variant and mutations.
① Variant type (composed of clade, WHO label)
② For example 22C, clicking on Omicron, variants and mutation data are provided.
③ In the bottom section 22C, Omicron spike protein 3D structure model provided.

Nextstrain

Main functions: Data analysis tool

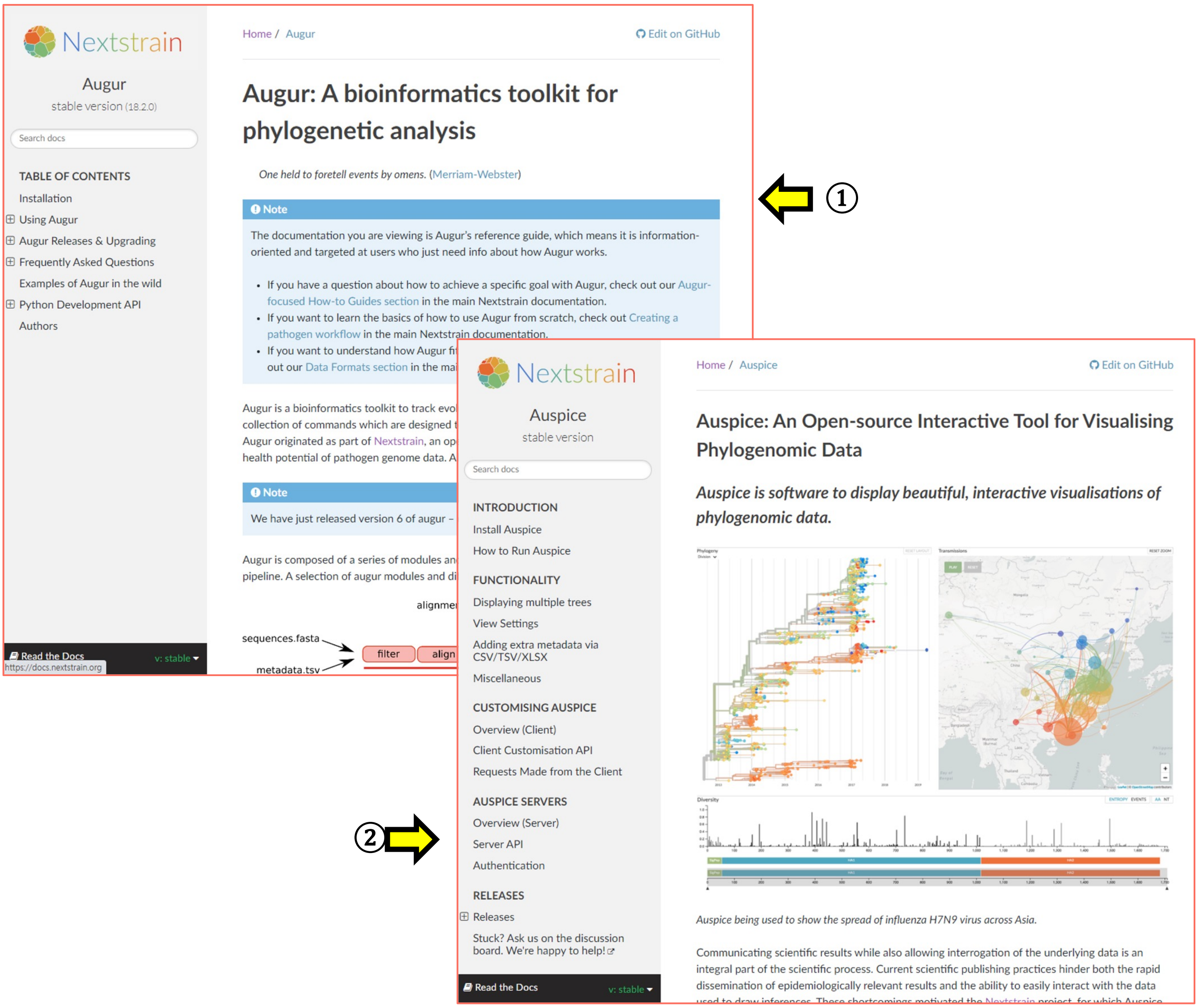

① **Augur**
https://docs.nextstrain.org/projects/augur/en/stable/index.html
Augur is a bioinformatics tool that tracks the evolution of sequence and serological data. Requires installation locally on personal computer.
Installation details are detailed in this address:
https://docs.nextstrain.org/projects/augur/en/stable/installation/installation.html
Usage is detailed in this page:
https://docs.nextstrain.org/projects/augur/en/stable/usage/usage.html

② **Auspice**
https://docs.nextstrain.org/projects/auspice/en/stable/index.html
Auspice is a tool that visualizes phylogenomic data. Also requires installation locally on personal computer. Installation is detailed here:
https://docs.nextstrain.org/projects/auspice/en/stable/introduction/install.html, **Usage is detailed in: https://docs.nextstrain.org/projects/auspice/en/stable/introduction/how-to-run.html**

NIH National Library of Medicine
National Center for Biotechnology Information

NCBI SARS-CoV-2 Resources

Main page

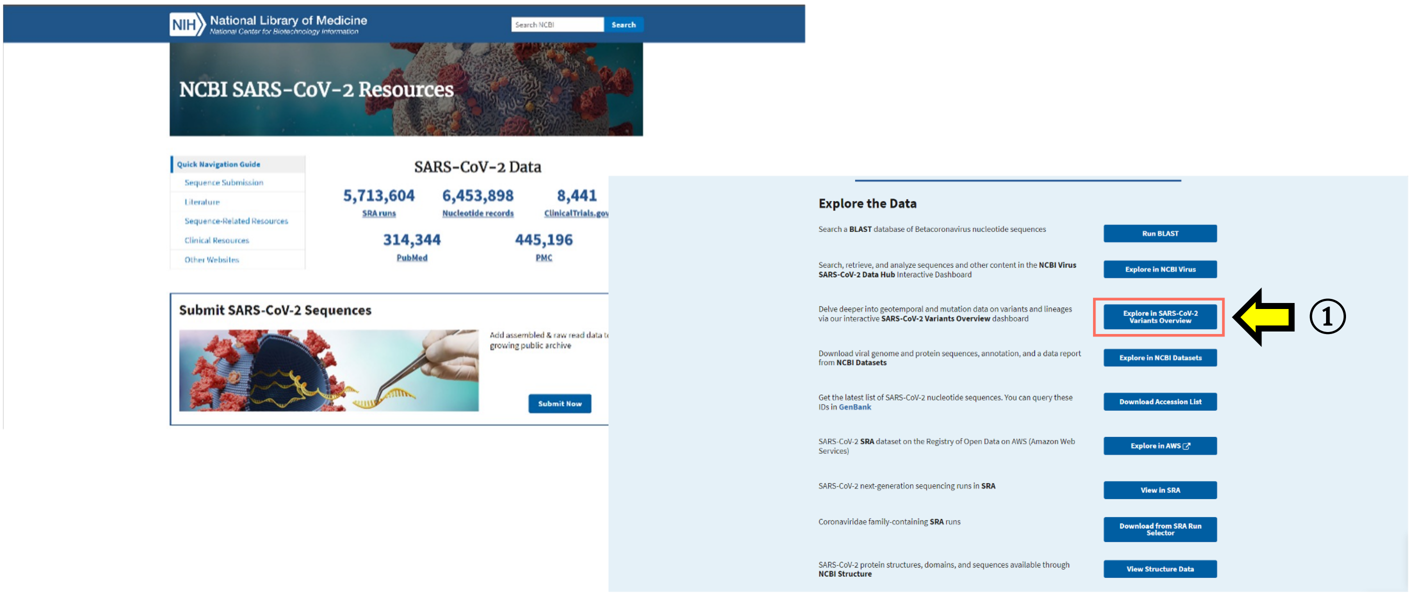

https://www.ncbi.nlm.nih.gov/sars-cov-2/

NIH provides SARS-CoV-2 resources and separates data into 3 categories: SARS-CoV-2 Literature, SARS-CoV-2 Sequence Resources, COVID-19 Clinical Resource

Main functions: Clade/variant/lineage

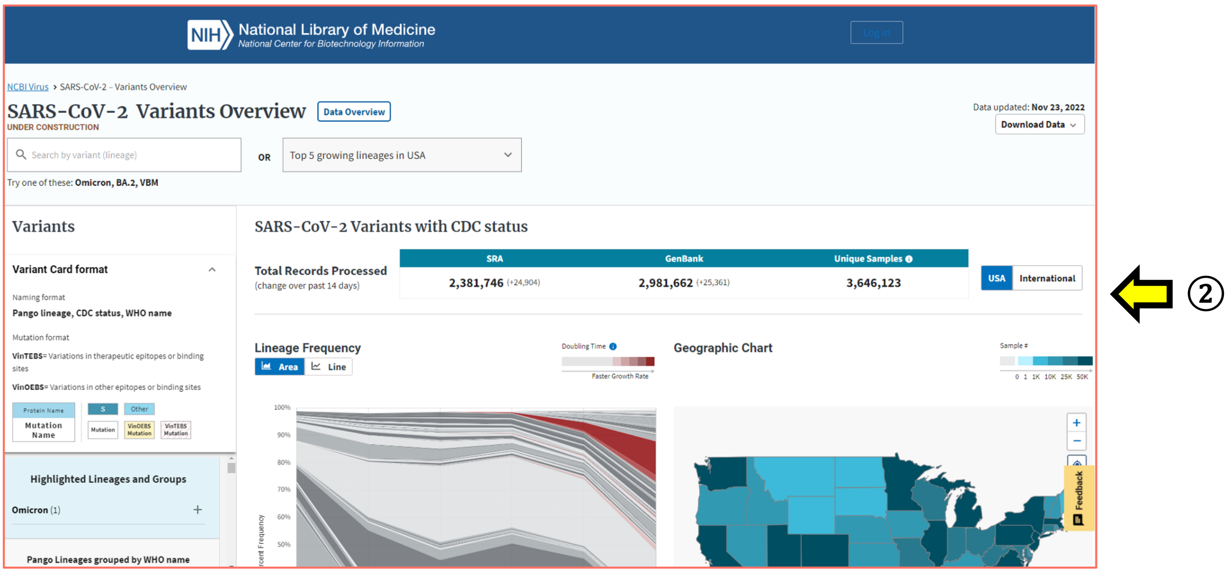

① **From SARS-CoV-2 Sequence Resources Explore the Data, click on Explore in SARS-CoV-2 Variants Overview**
② **Geotemporal and mutation data provided**

National Library of Medicine
National Center for Biotechnology Information

NCBI SARS-CoV-2 Resources

Main functions: Genome browser (sequence)

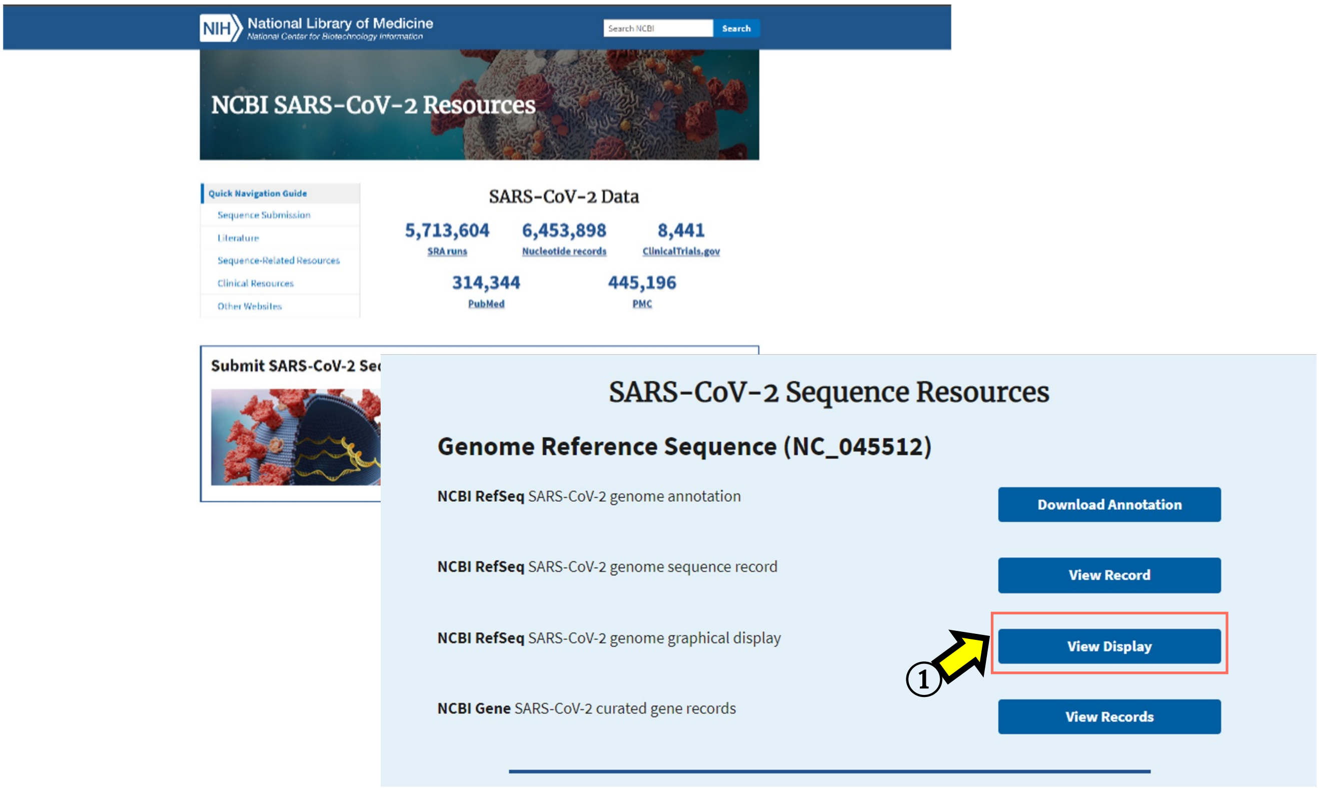

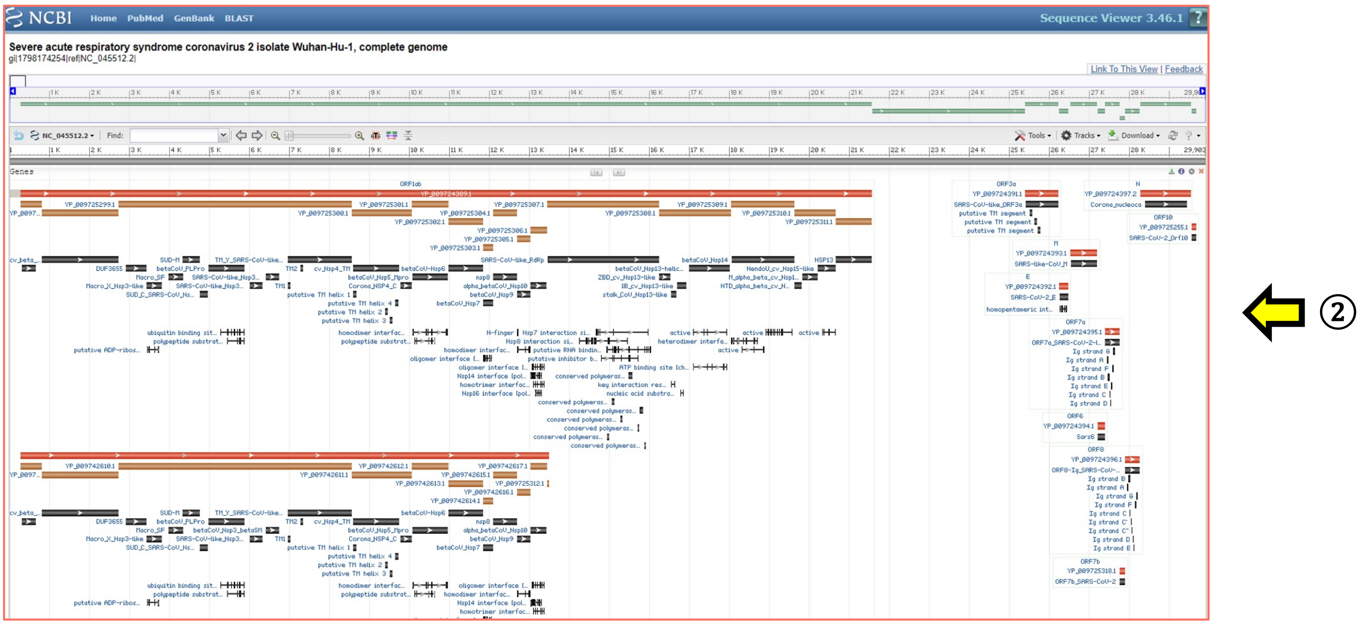

① From SARS-CoV-2 Sequence Resources Genome Reference Sequence (NC_045512), click on View Display
② Position browse inside NCBI RefSeq SARS-CoV-2 genome sequence

Main functions: Protein structure

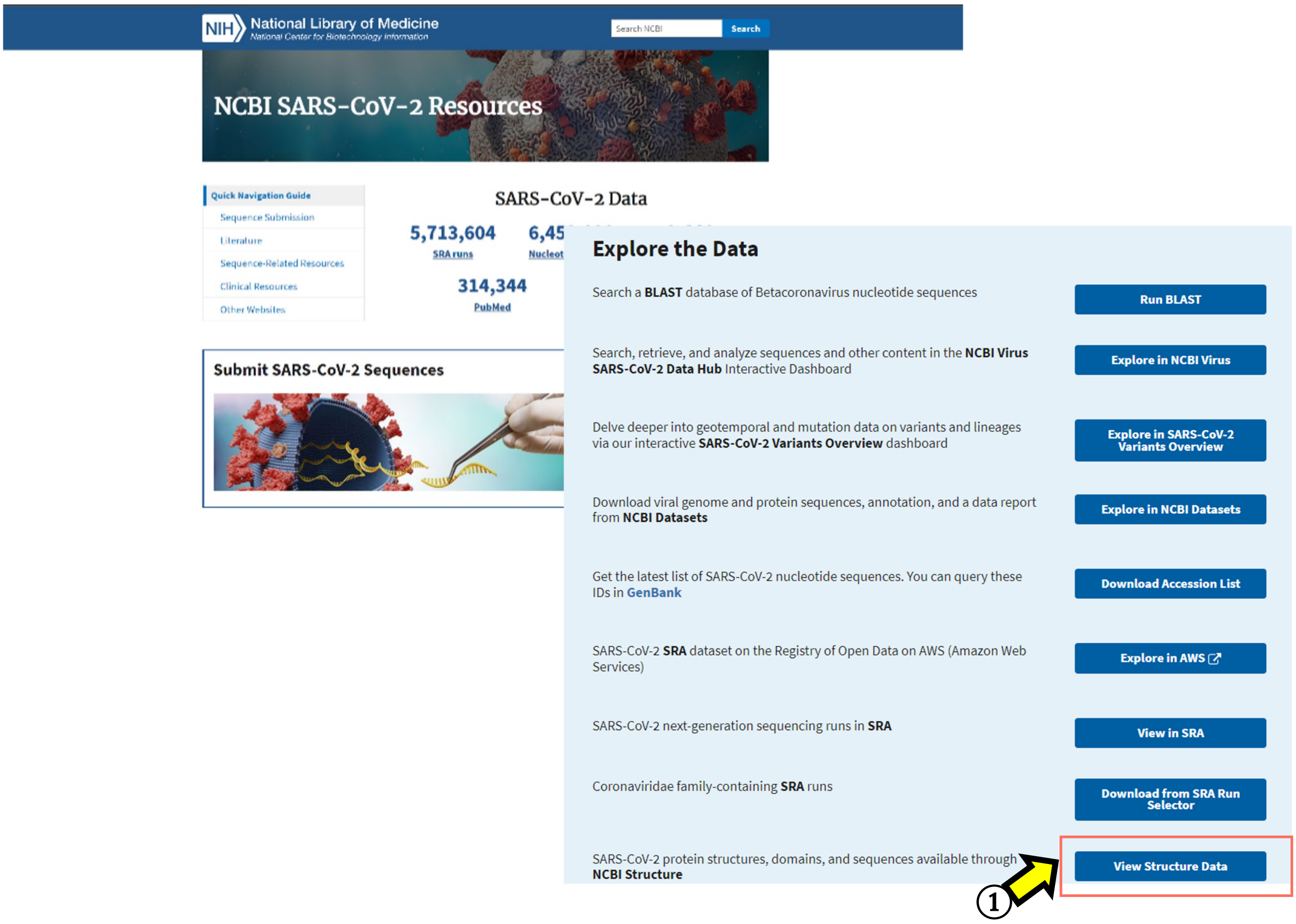

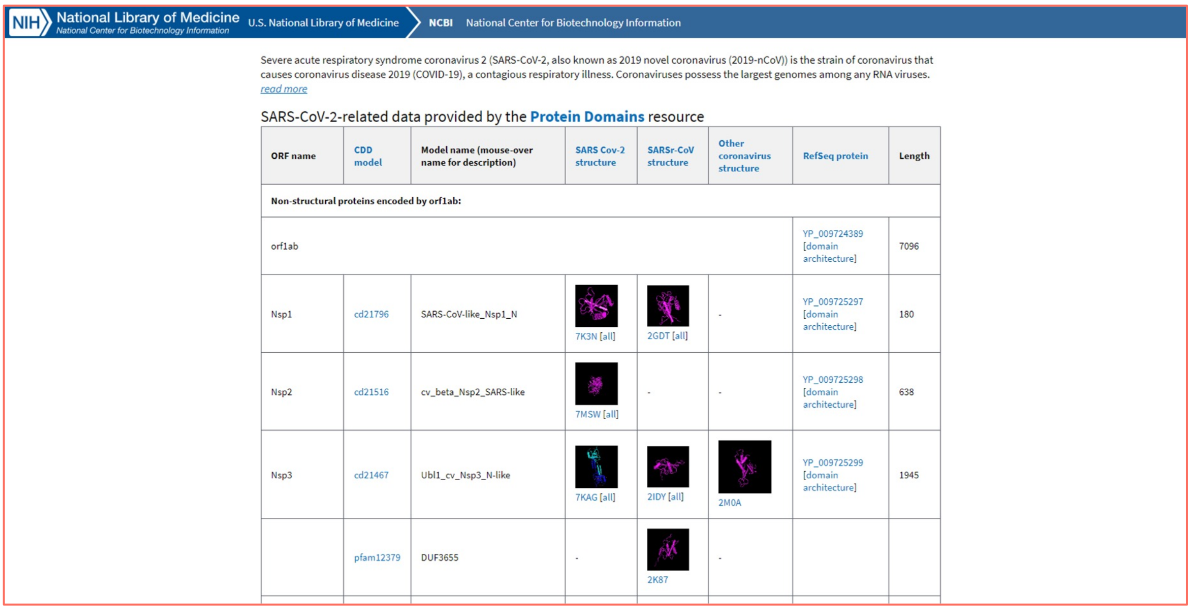

②

① From SARS-CoV-2 Sequence Resources Explore the Data, click on View Structure Data
② SARS-CoV-2 protein structures, domains, sequences data provided

Main functions: Visualization

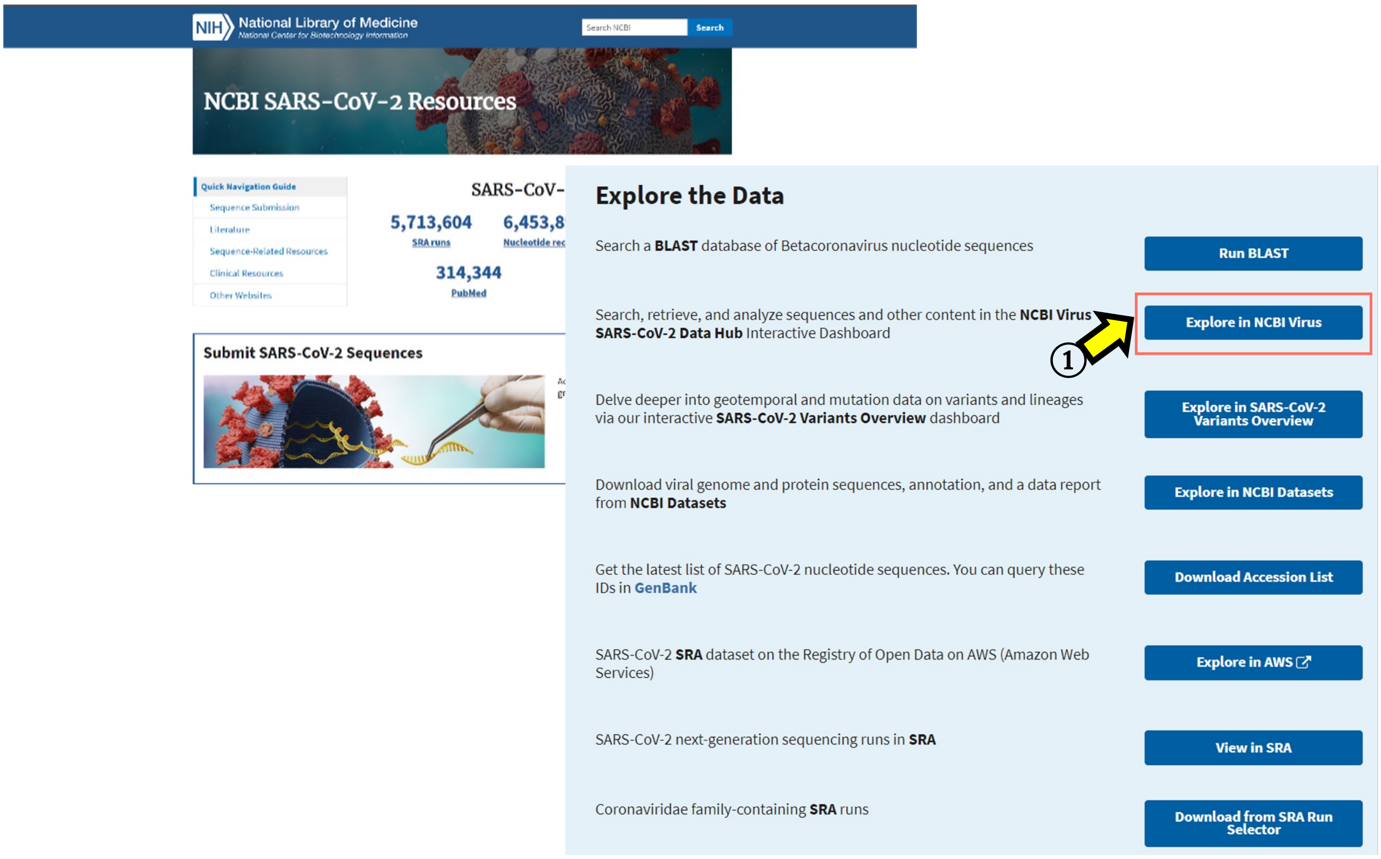

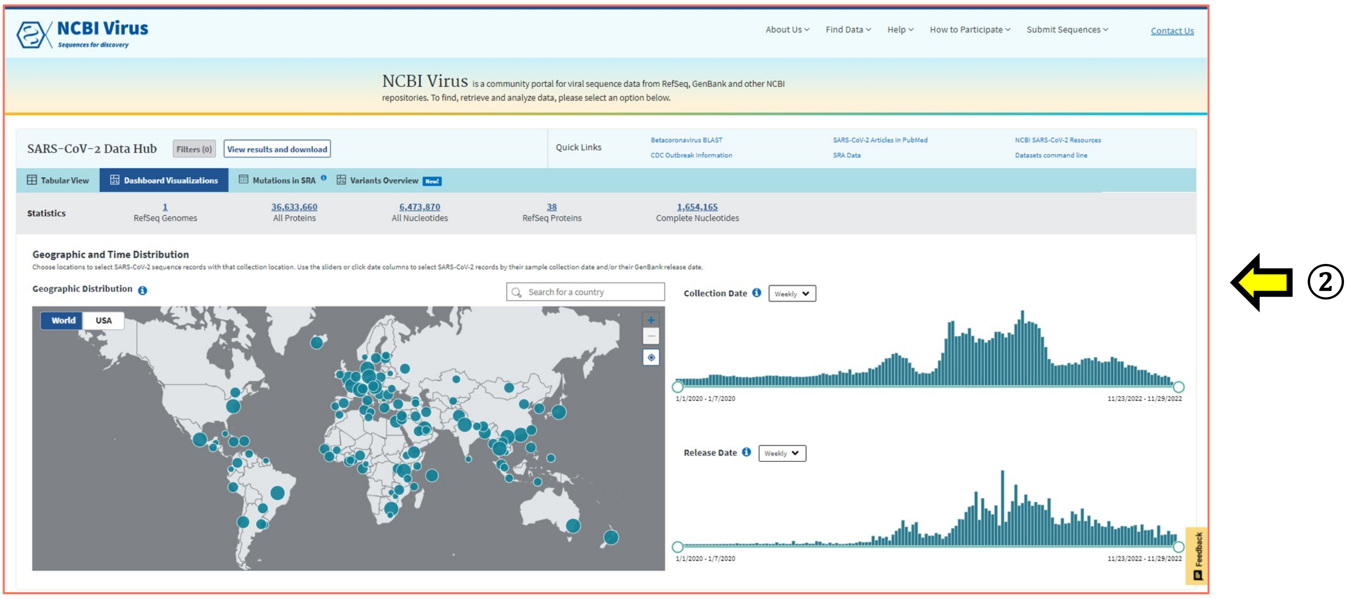

① From SARS-CoV-2 Sequence Resources Explore the Data, click on Explore in NCBI Virus
② SARS-CoV-2 data visualization

NIH National Library of Medicine
National Center for Biotechnology Information

NCBI SARS-CoV-2 Resources

Main functions: Visualization

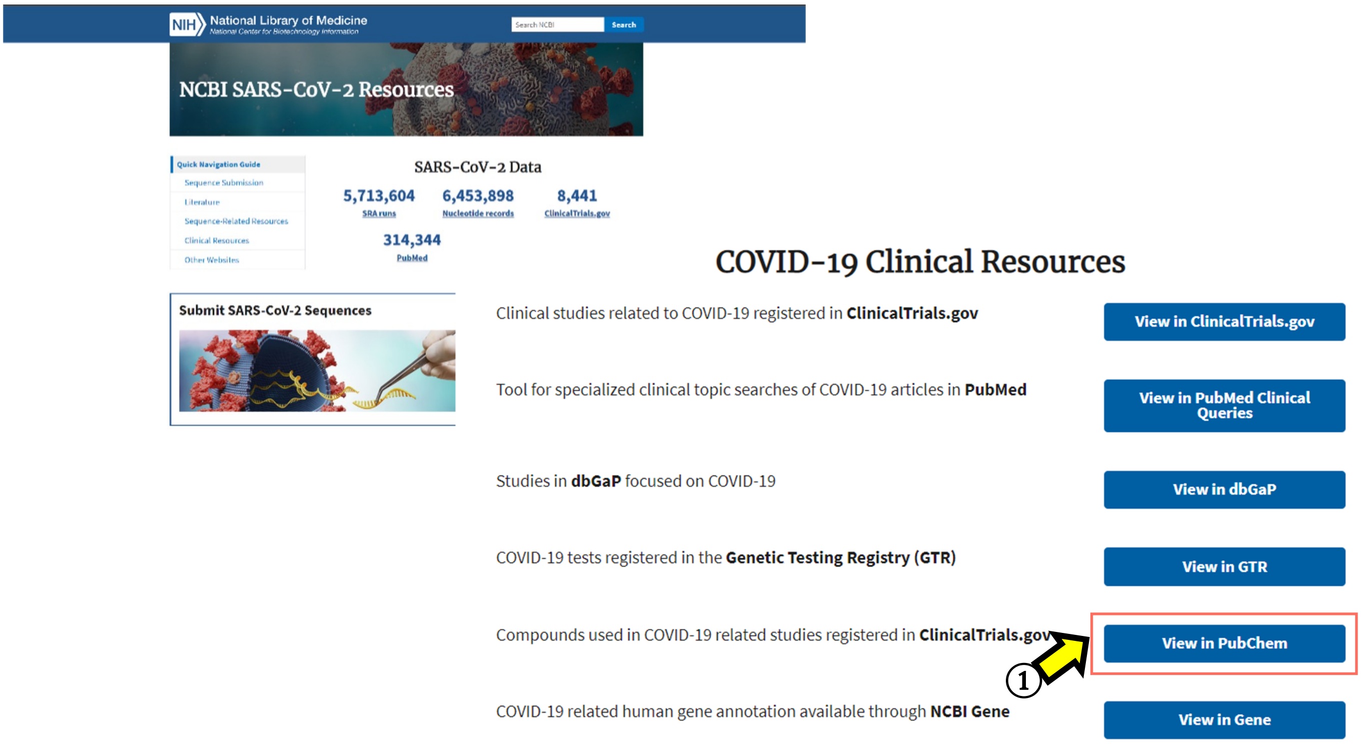

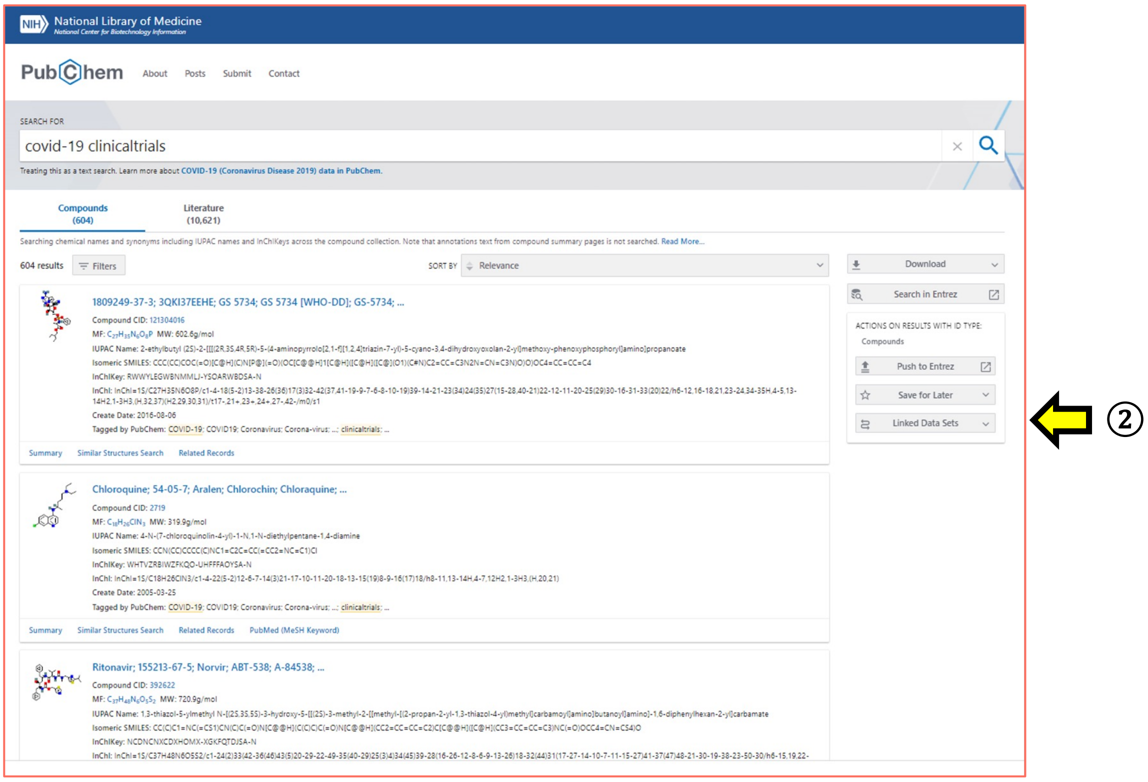

① From COVID-19 Clinical Resources, click on View in PubChem
② From ClinicalTrial.gov, used compounds data, chemical structure, and 3D conformer provided

NIH National Library of Medicine
National Center for Biotechnology Information

NCBI SARS-CoV-2 Resources

Main functions: Data analysis tool

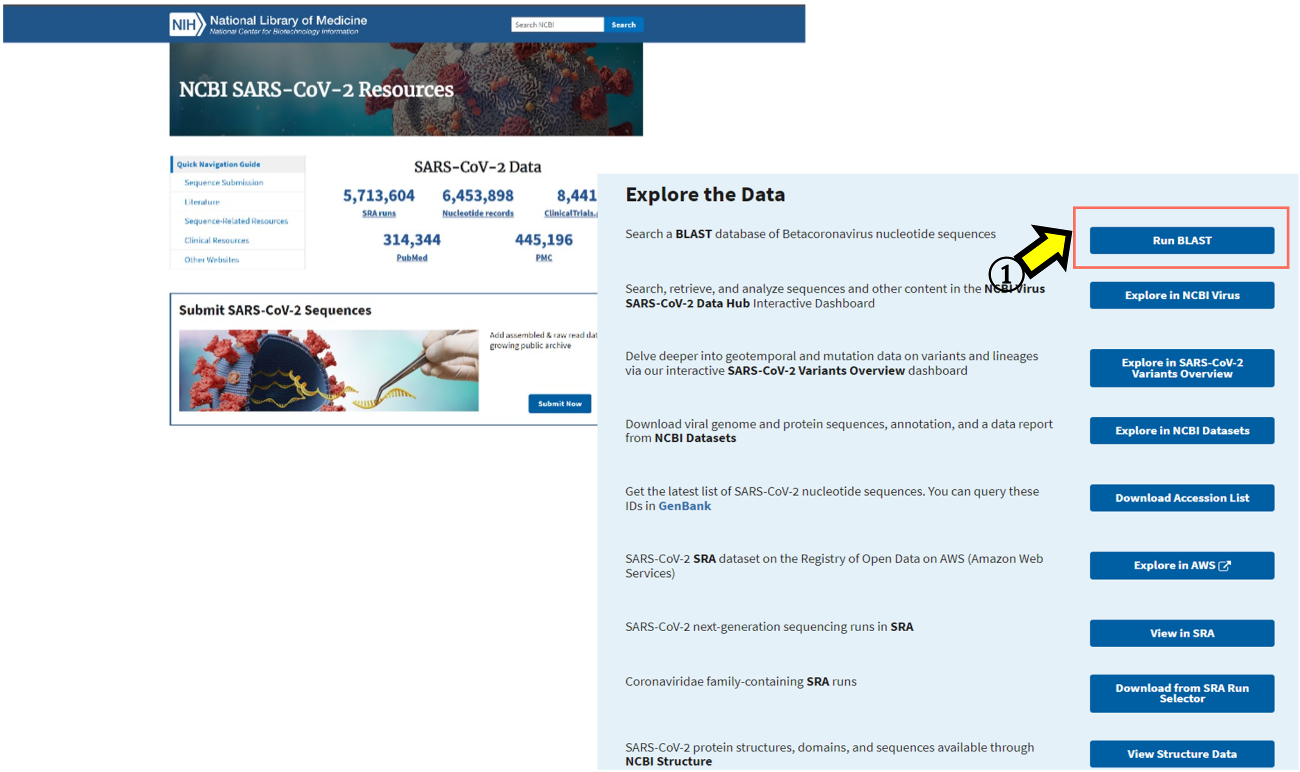

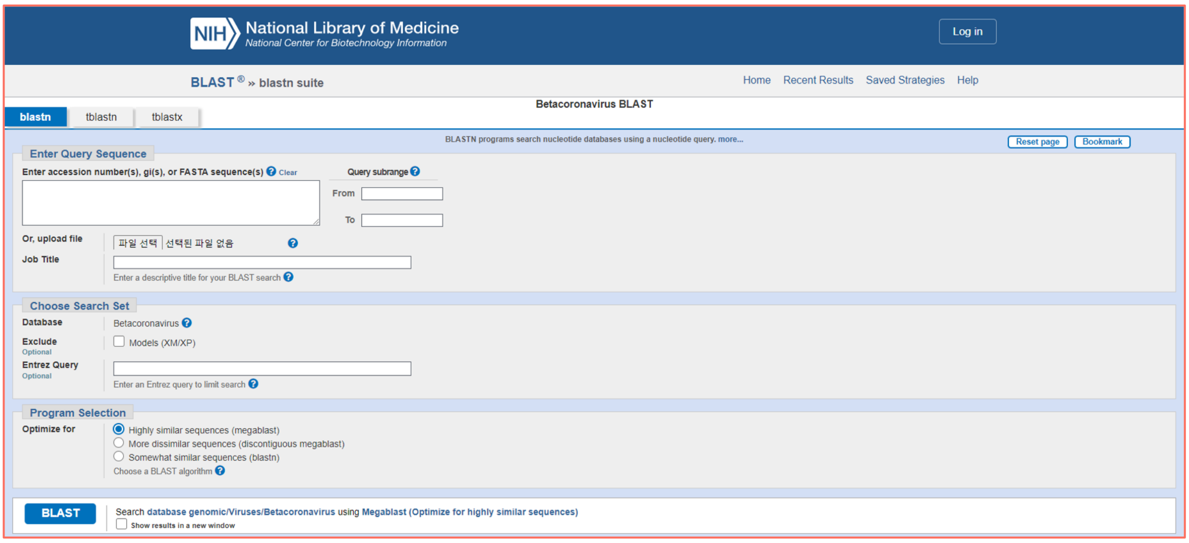

②

① **From SARS-CoV-2 Sequence Resources Explore the Data, click on Run BLAST**
② **Betacoronavirus nucleotide sequences database can be investigated**

National Library of Medicine
National Center for Biotechnology Information

NCBI SARS-CoV-2 Resources

Main functions: Literature

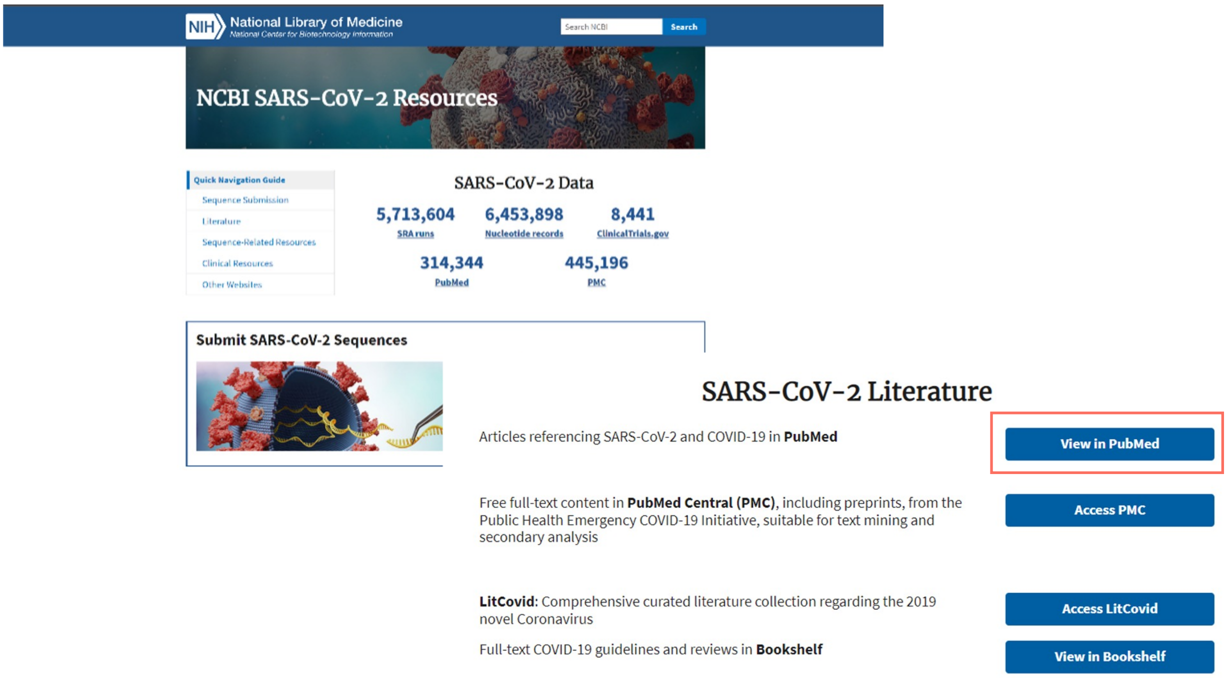

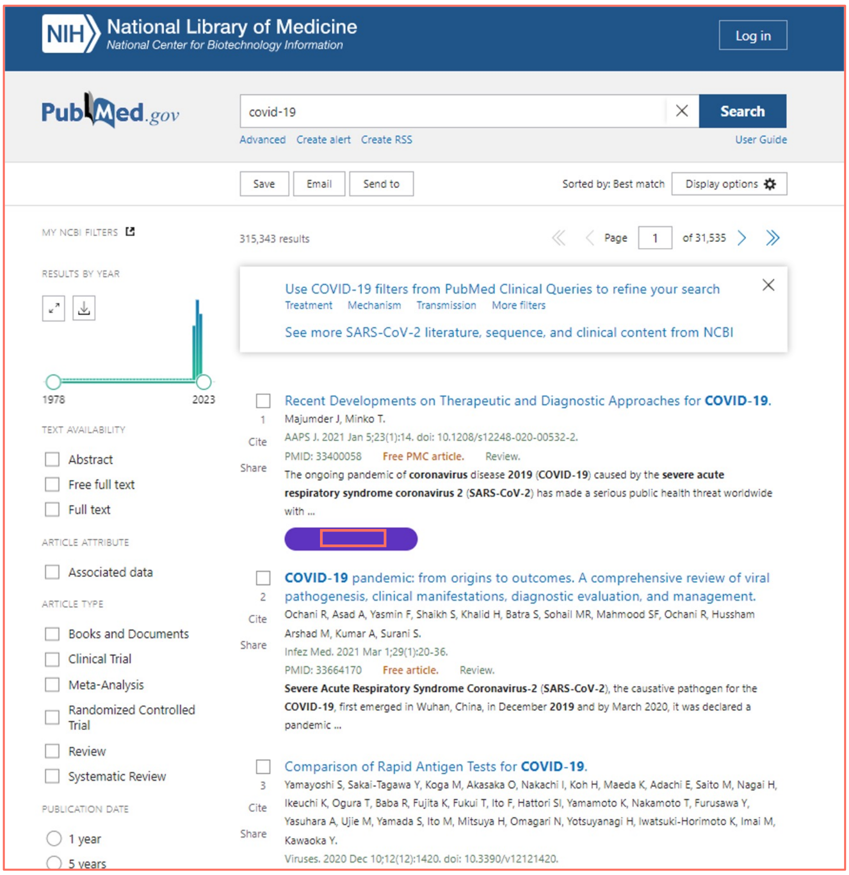

②

① From SARS-CoV-2 Literature, click on View in PubMed
② Articles referencing SARS-CoV-2 and COVID-19 in PubMed

National Library of Medicine
National Center for Biotechnology Information

NCBI SARS-CoV-2 Resources

Main functions: Literature

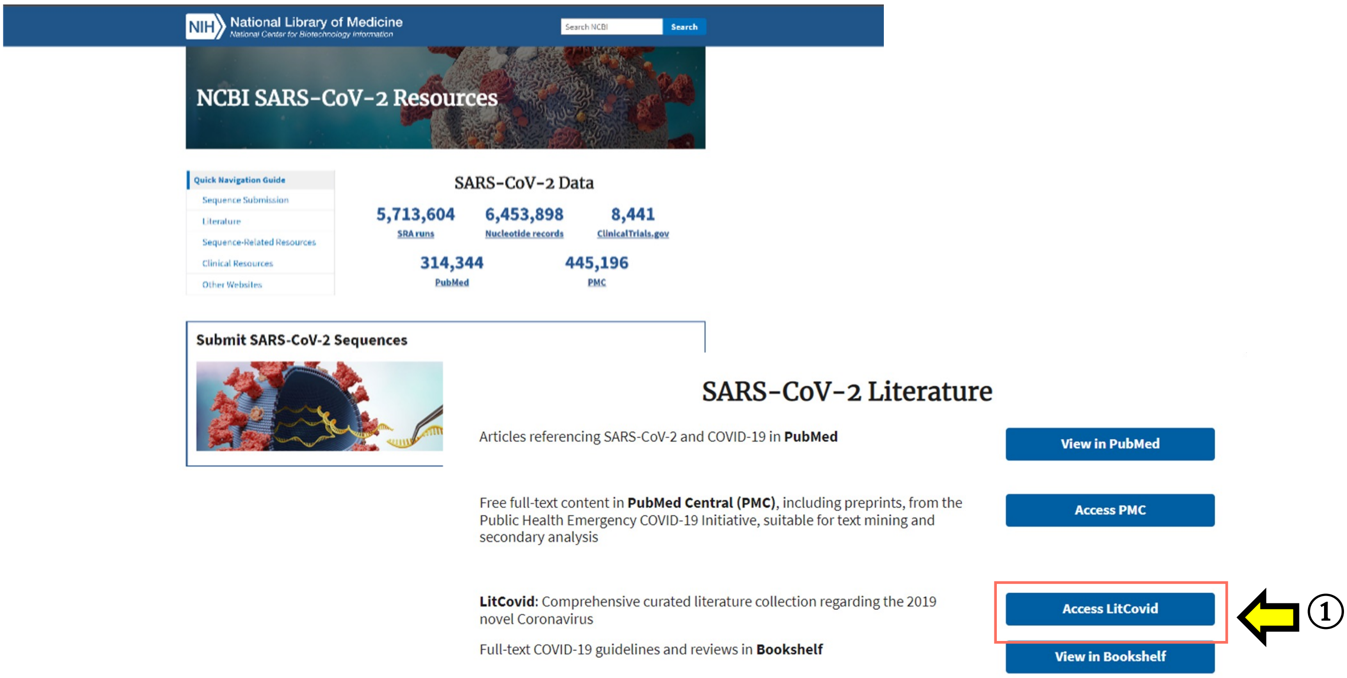

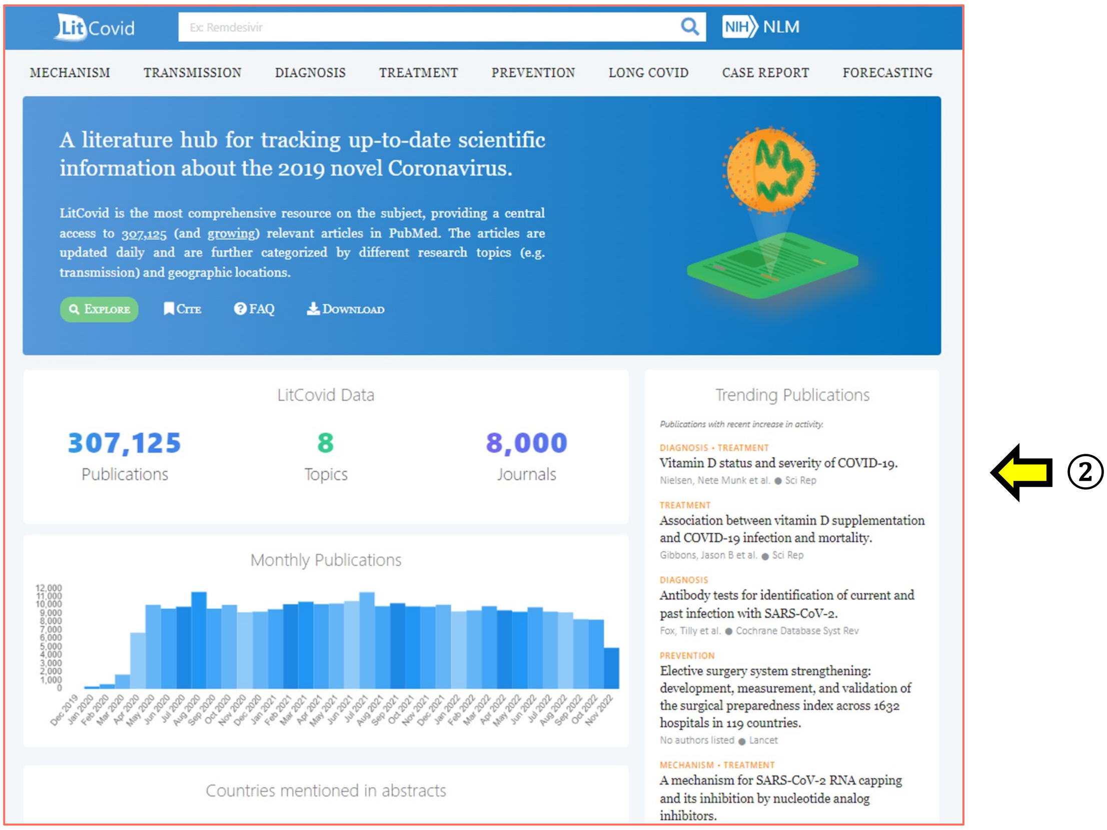

① From SARS-CoV-2 Literature, click on View in PubMed
② Comprehensive curated literature collection regarding the 2019 novel Coronavirus

Main page

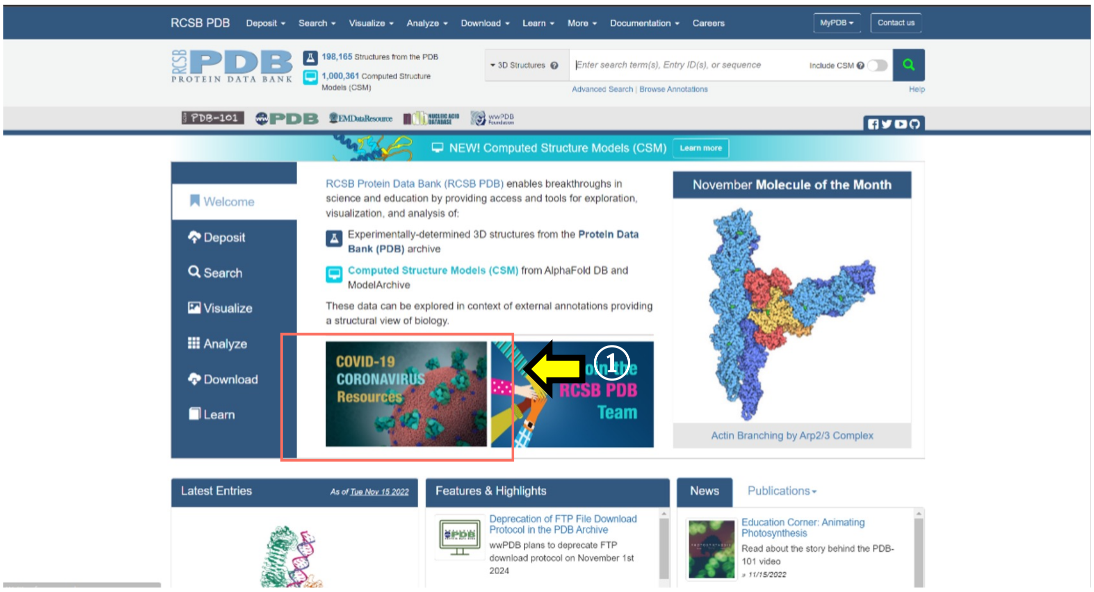

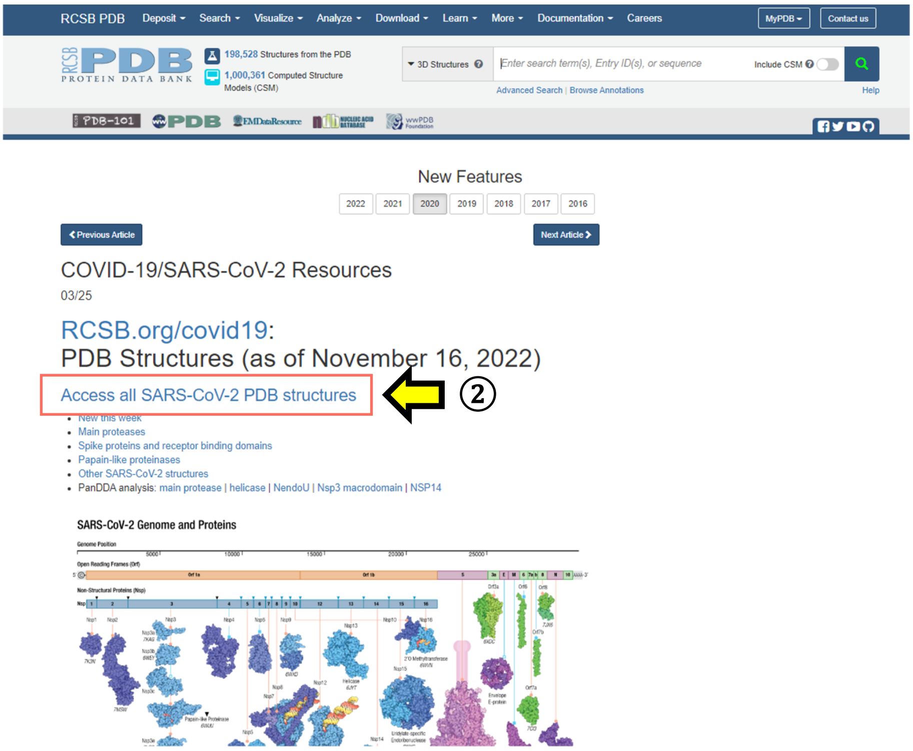

https://www.rcsb.org/

① Protein Data Bank (PDB) is a website that provides 3-D structure and data on molecules. Through covid-19 coronavirus resources, SARS-CoV-2 protein data is provided.
② Click on Access all SARS-CoV-2 PDB structures

Main functions: Protein structure, Visualization

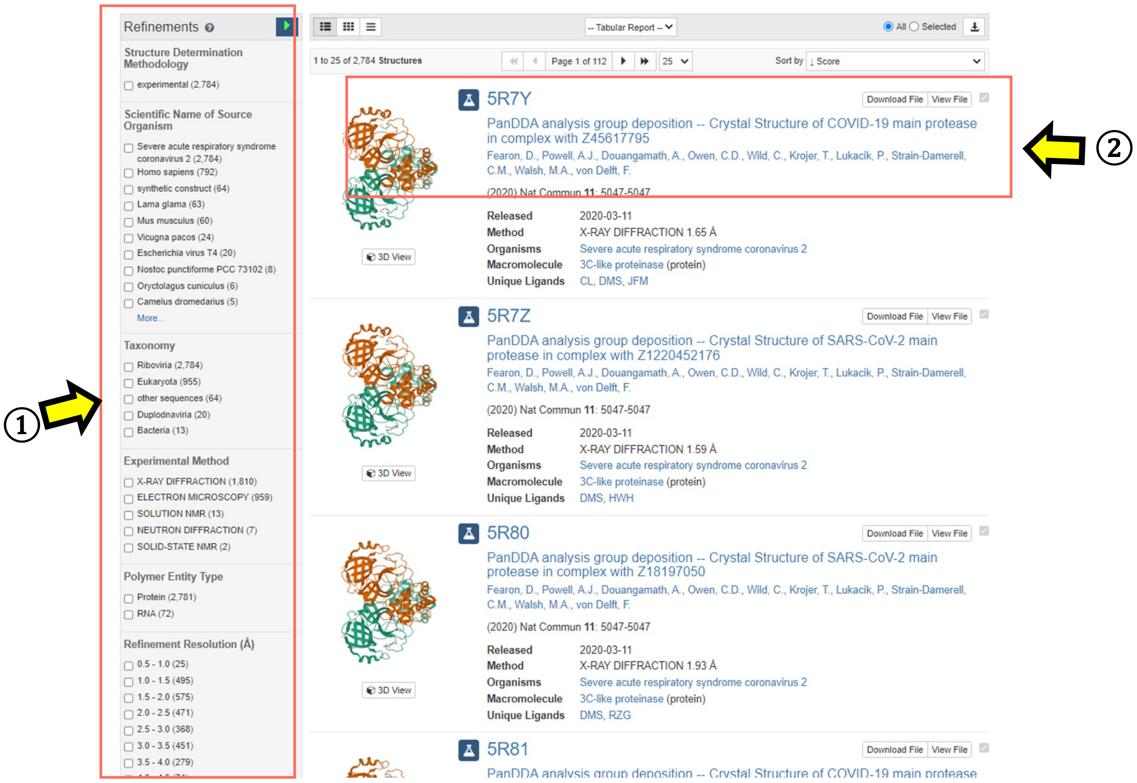

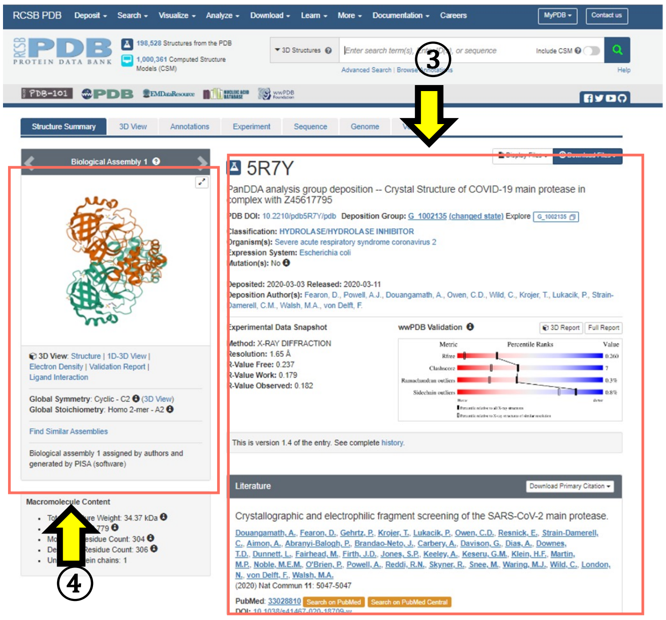

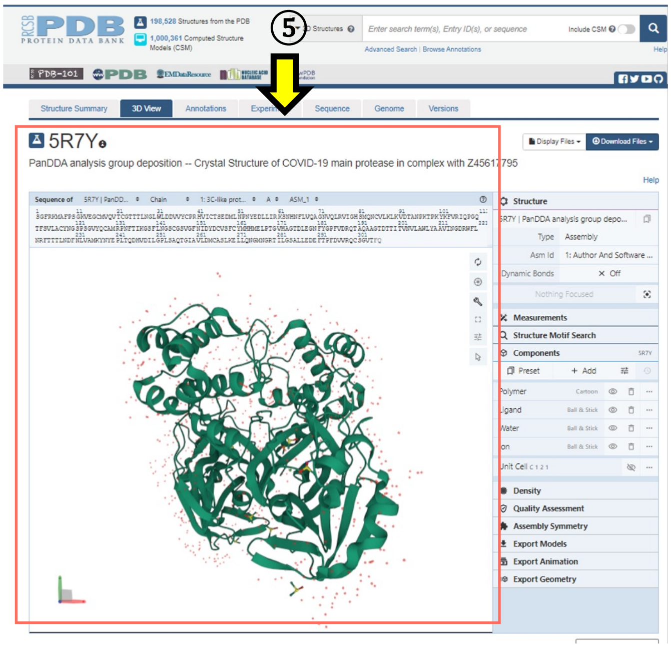

① Possible to select search options
② For example, select 5R7Y protein
③ 5R7Y data provided: Literature, Macromolecules, Small Molecules, Experimental Data & Validation
④ Protein 3D view
⑤ Protein 3D structure and sequence provided

Main functions: Data analysis tool

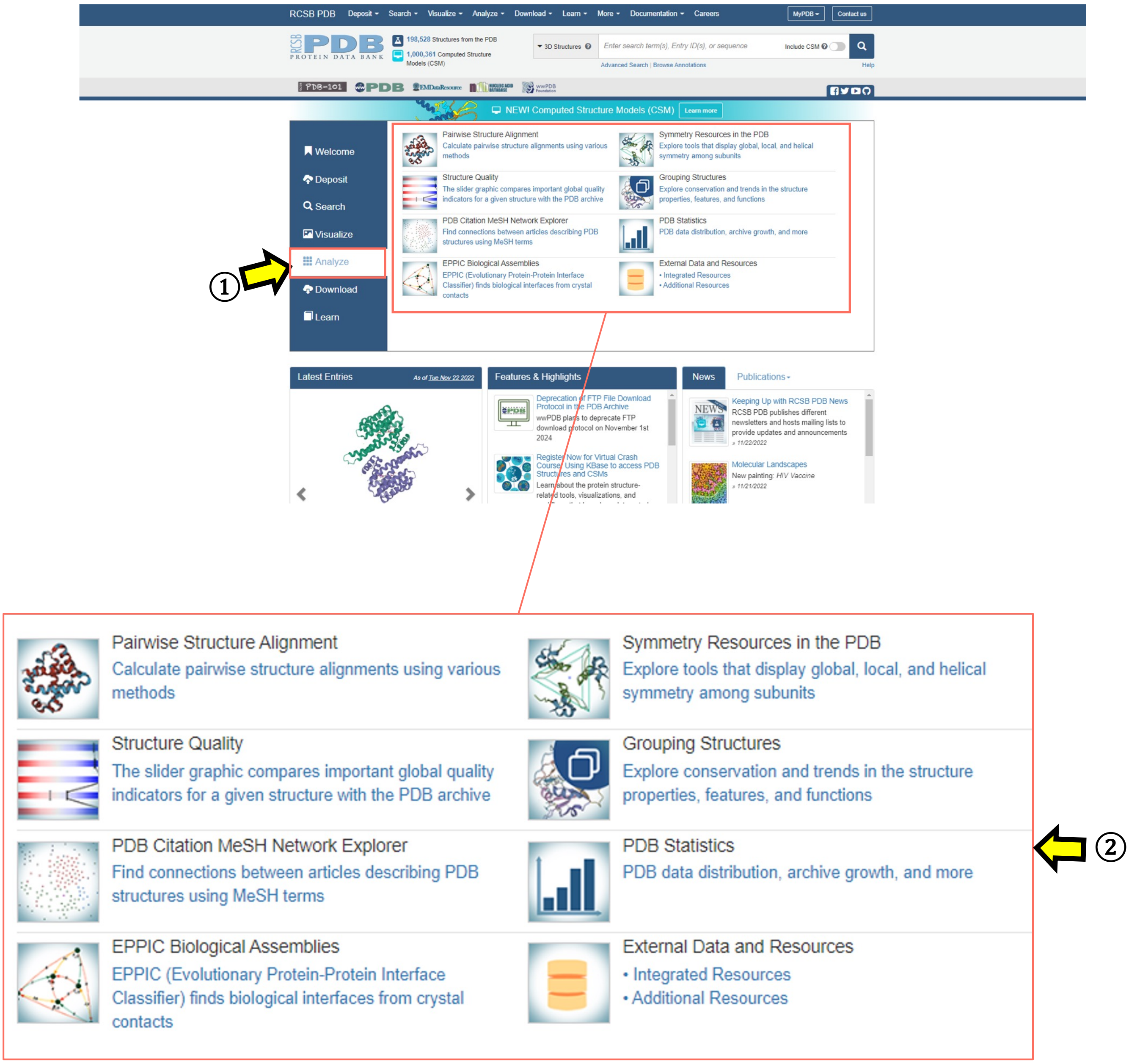

① Click on Analyze
② Pairwise Structure Alignment, Symmetry Resources in the PDB, Structure Quality, Grouping Structures, PDB Citation MeSH Network Explorer, PDB Statistics, EPPIC Biological Assemblies

Main functions: Literature

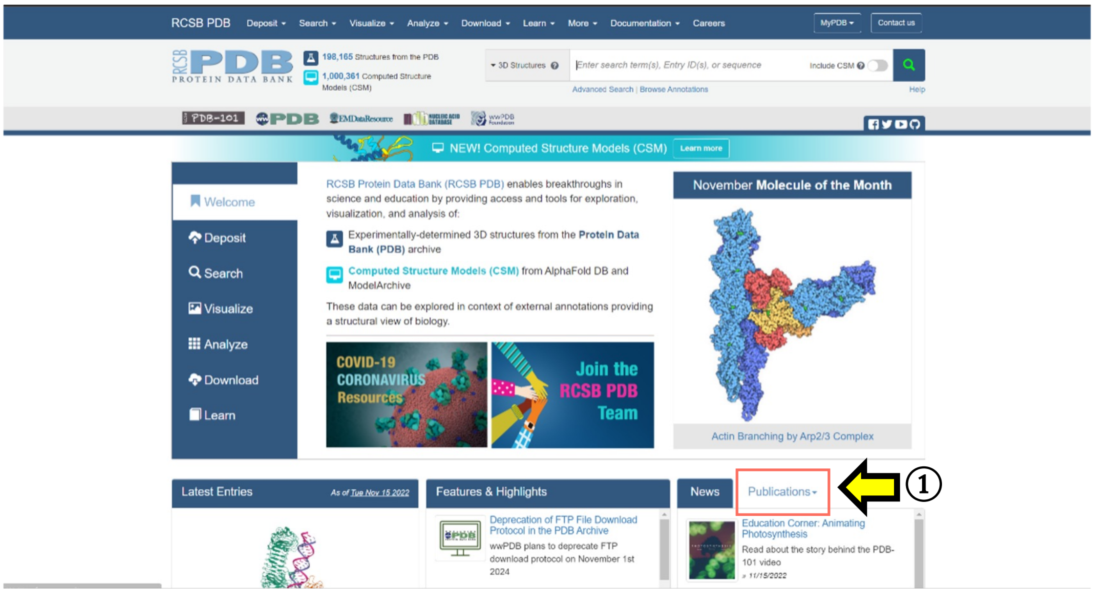

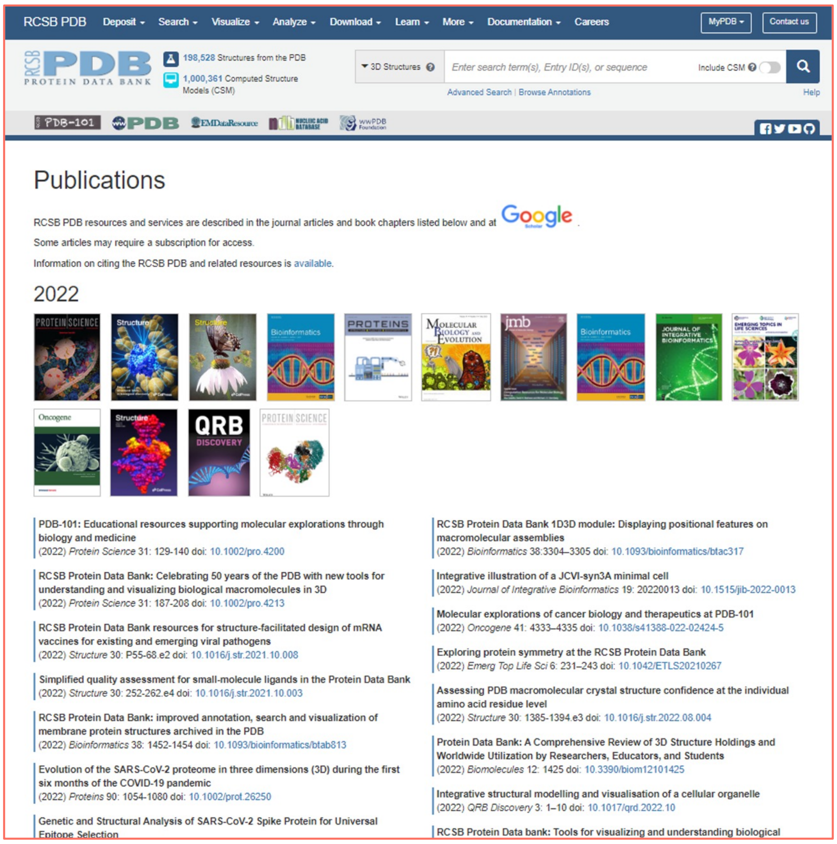

②

① Click on Publications
② Journal articles and book chapters list introduced

Main page

Main functions: Clade/variant/lineage

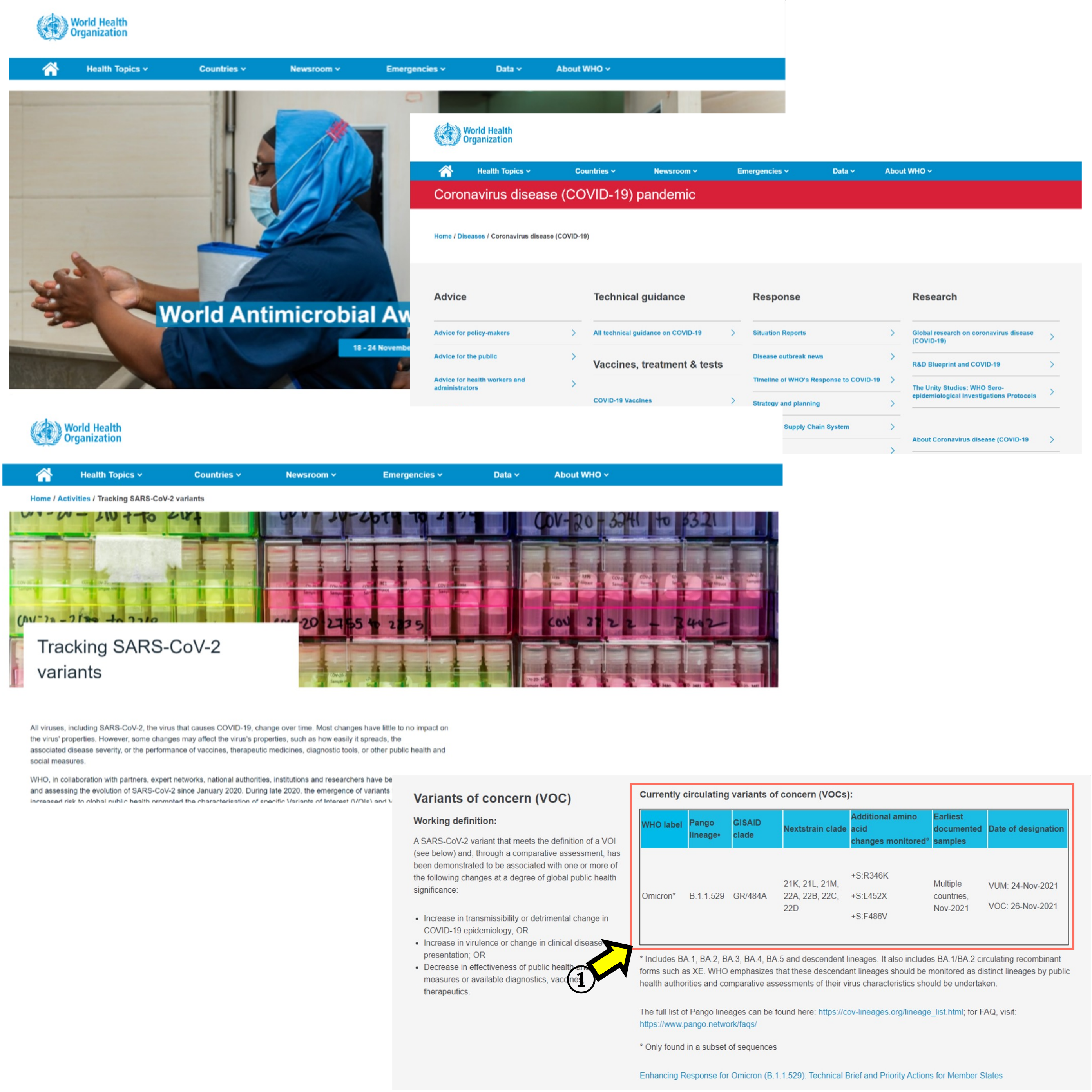

https://www.who.int/

The World Health Organization provides data on infectious disease COVID-19 caused by the Coronavirus.
① Through tracking SARS-CoV-2 variants, data on currently circulating variants of concern (VOCs) and past VOCs are provided

Main functions: Epidemiological data, Visualization

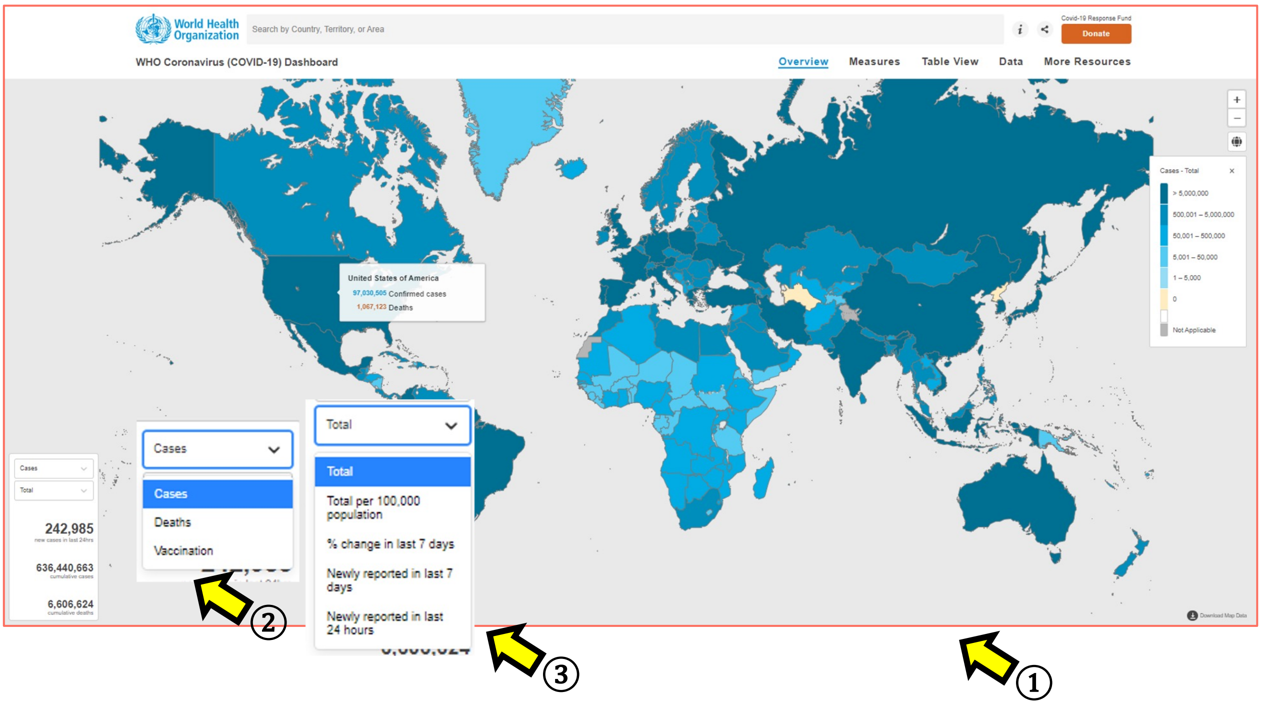

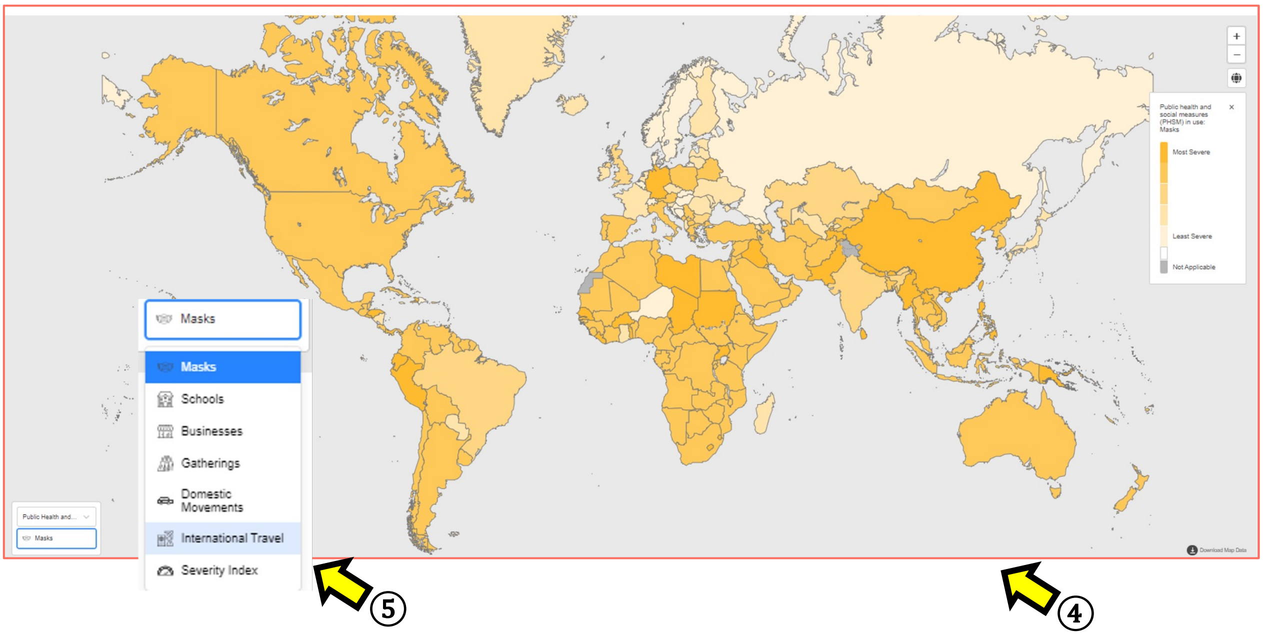

① In upper tab, select Overview
② Select Cases, Deaths, Vaccination
③ Total, Total per 100,000 population, % change in last 7 days, Newly reported in last 7 days, Newly reported in last 24 hours

Main functions: Epidemiological data, Visualization

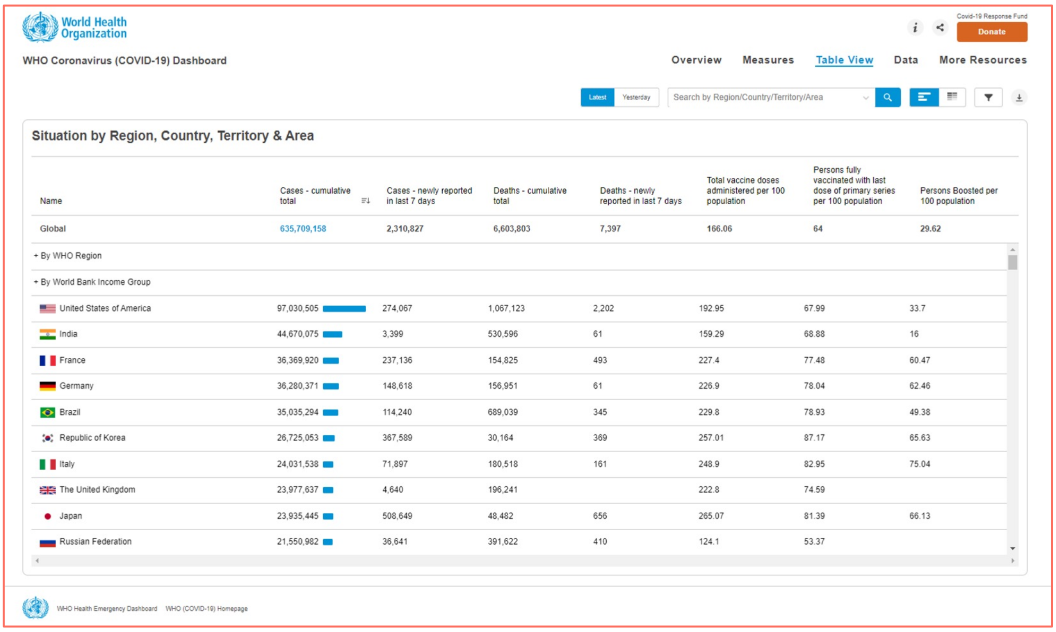

World Health Organization

WHO Coronavirus (COVID-19) Dashboard

Overview | Measures | Table View | Data | More Resources

Situation by Region, Country, Territory & Area

Name	Cases - cumulative total	Cases - newly reported in last 7 days	Deaths - cumulative total	Deaths - newly reported in last 7 days	Total vaccine doses administered per 100 population	Persons fully vaccinated with last dose of primary series per 100 population	Persons Boosted per 100 population
Global	635,709,158	2,310,827	6,603,803	7,397	166.06	64	29.62
+ By WHO Region							
+ By World Bank Income Group							
United States of America	97,030,505	274,067	1,067,123	2,202	192.95	67.99	33.7
India	44,670,075	3,399	530,596	61	159.29	68.88	16
France	36,369,920	237,136	154,825	493	227.4	77.48	60.47
Germany	36,280,371	148,618	156,951	61	226.9	78.04	62.46
Brazil	35,035,294	114,240	689,039	345	229.8	78.93	49.38
Republic of Korea	26,725,053	367,589	30,164	369	257.01	87.17	65.63
Italy	24,031,538	71,897	180,518	161	248.9	82.95	75.04
The United Kingdom	23,977,637	4,640	196,241		222.8	74.59	
Japan	23,935,445	508,649	48,482	656	265.07	81.39	66.13
Russian Federation	21,550,982	36,641	391,622	410	124.1	53.37	

①

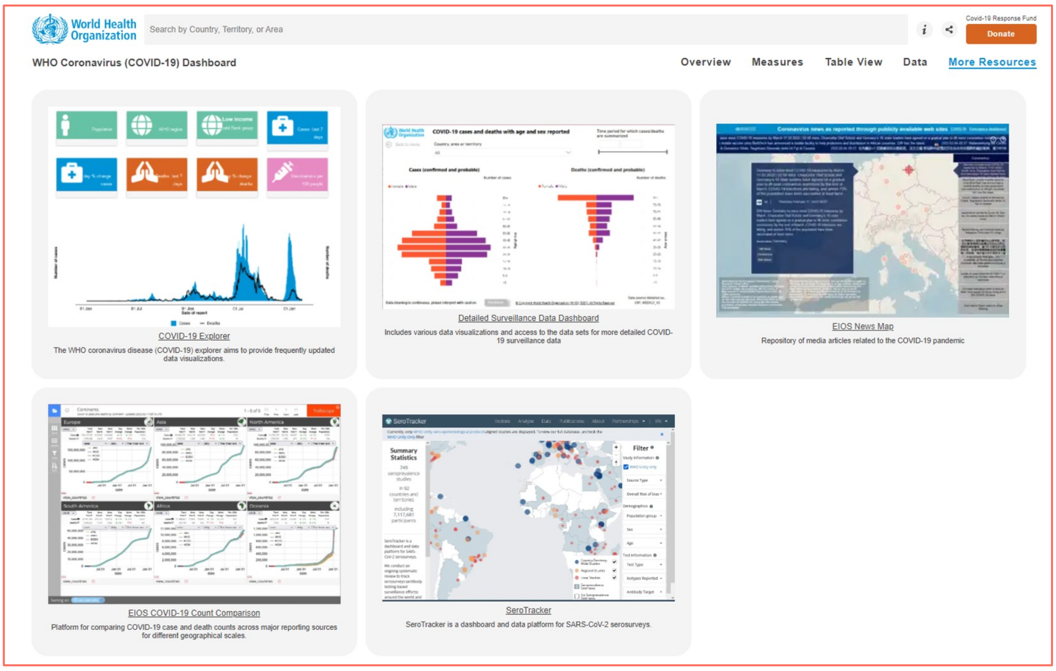

②

① In the upper tab, select Table view to visualize epidemiological data in table format
② In the upper tab, select more Resources, COVID-19 Explorer, Detailed Surveillance Data Dashboard, EIOS News Map, EIOS COVID-19 Count Comparison, SeroTracker provided

Main functions: Treatment (clinical trials, drug)

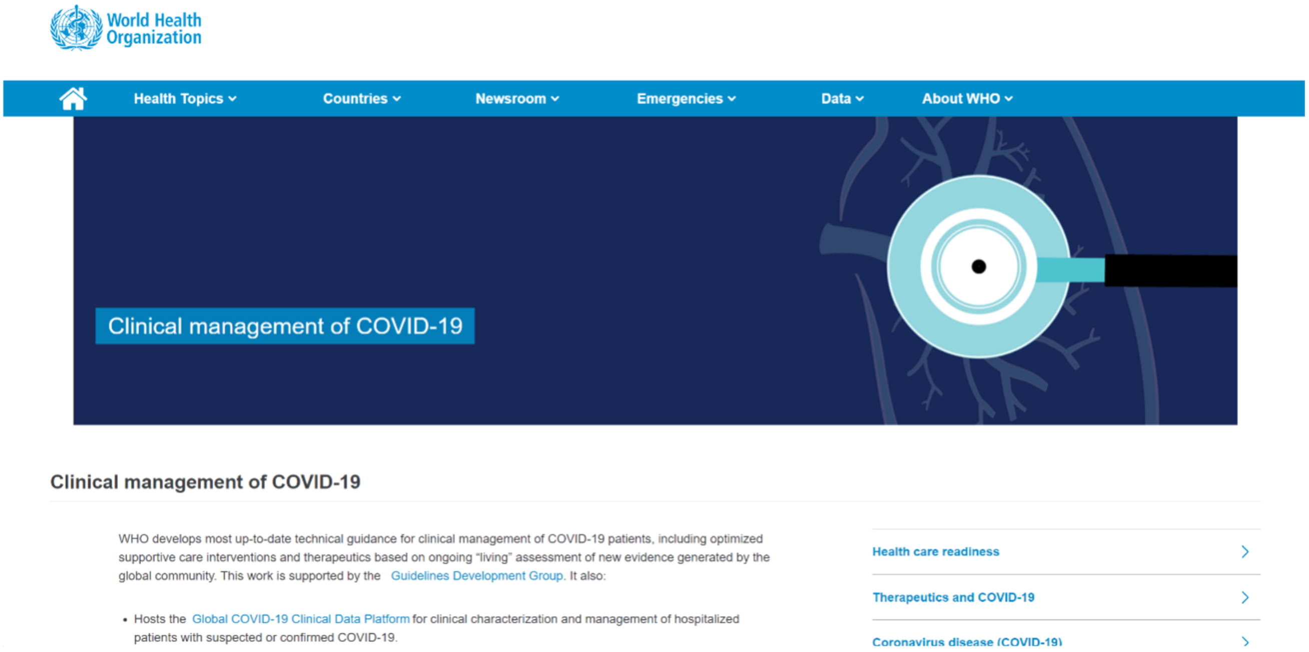

https://www.who.int/teams/health-care-readiness/covid-19

The WHO constantly assesses evidence created from worldwide communities. Optimized supportive treatment intervention and treatment methods are outlined. Latest technological guidelines and developments for clinical management of COVID-19 patients are provided.

Main functions: Literature

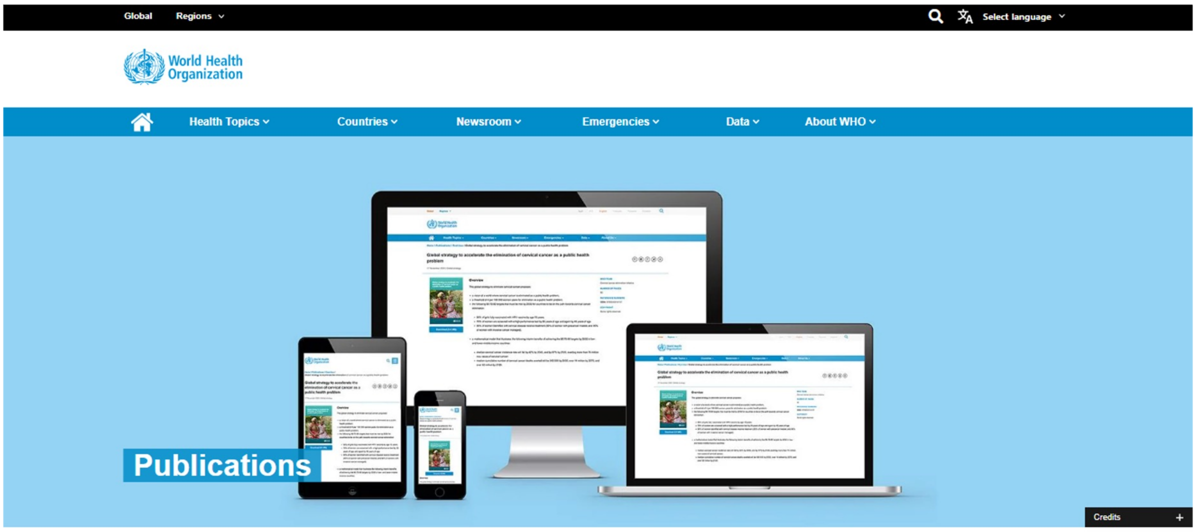

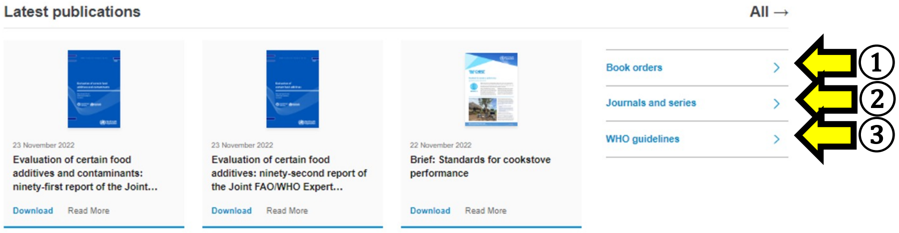

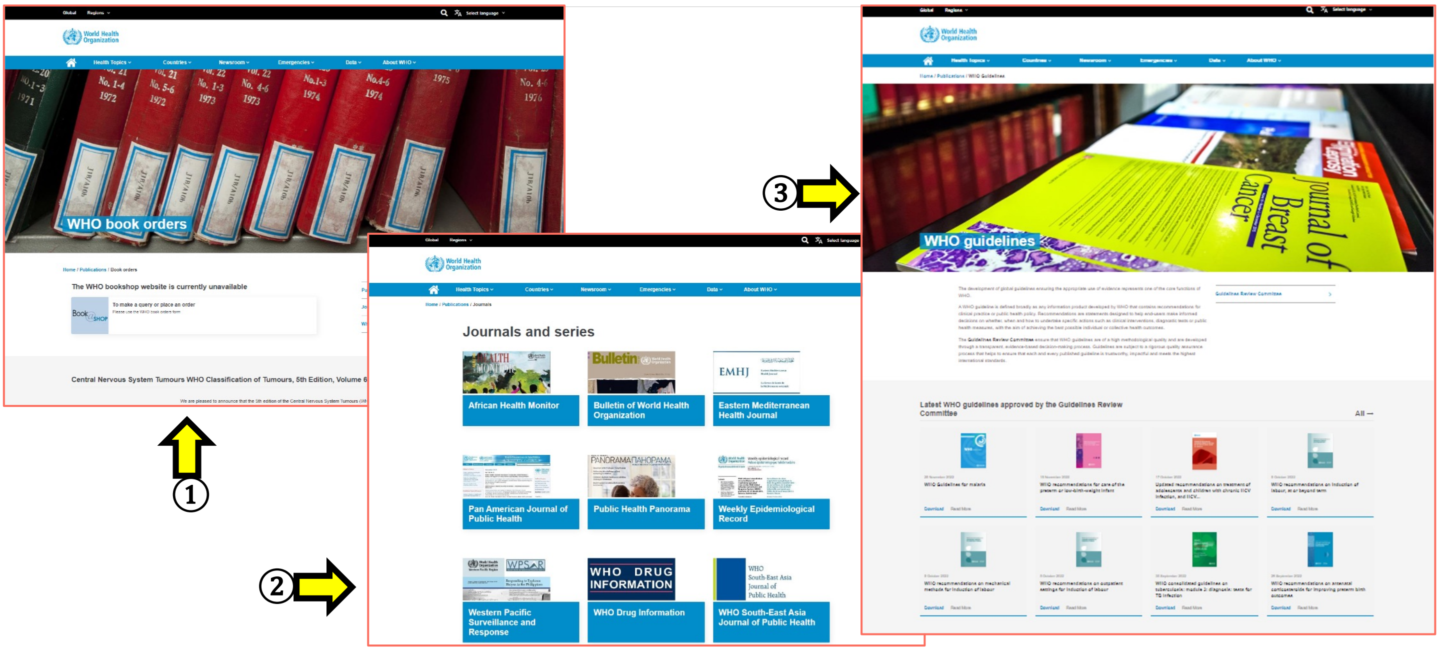

https://www.who.int/publications

① Book
② Journals and series
③ WHO guidelines

COVID-19 Research at UCSC

Main page

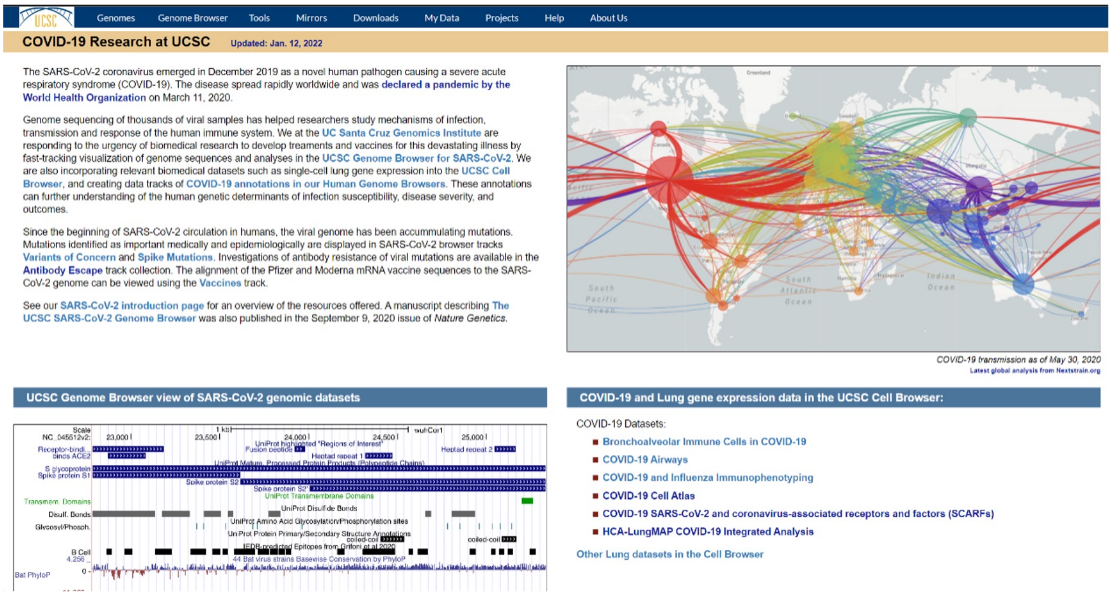

https://genome.ucsc.edu/covid19.html

Fast visualization and analysis of Genome sequence are provided, single cell lung gene expression data is provided

Main functions: Genome browser (sequence), Clade/variant/lineage

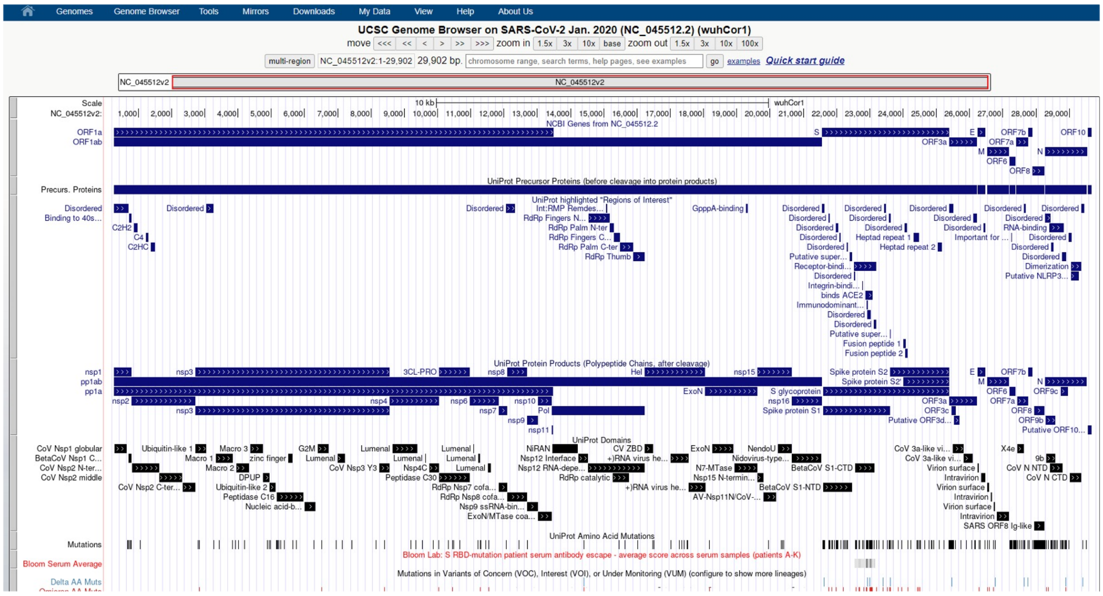

UCSC Genome Browser view of SARS-CoV-2 genomic datasets

COVID-19 Research at UCSC

Main functions: Genome browser (sequence), Clade/variant/lineage

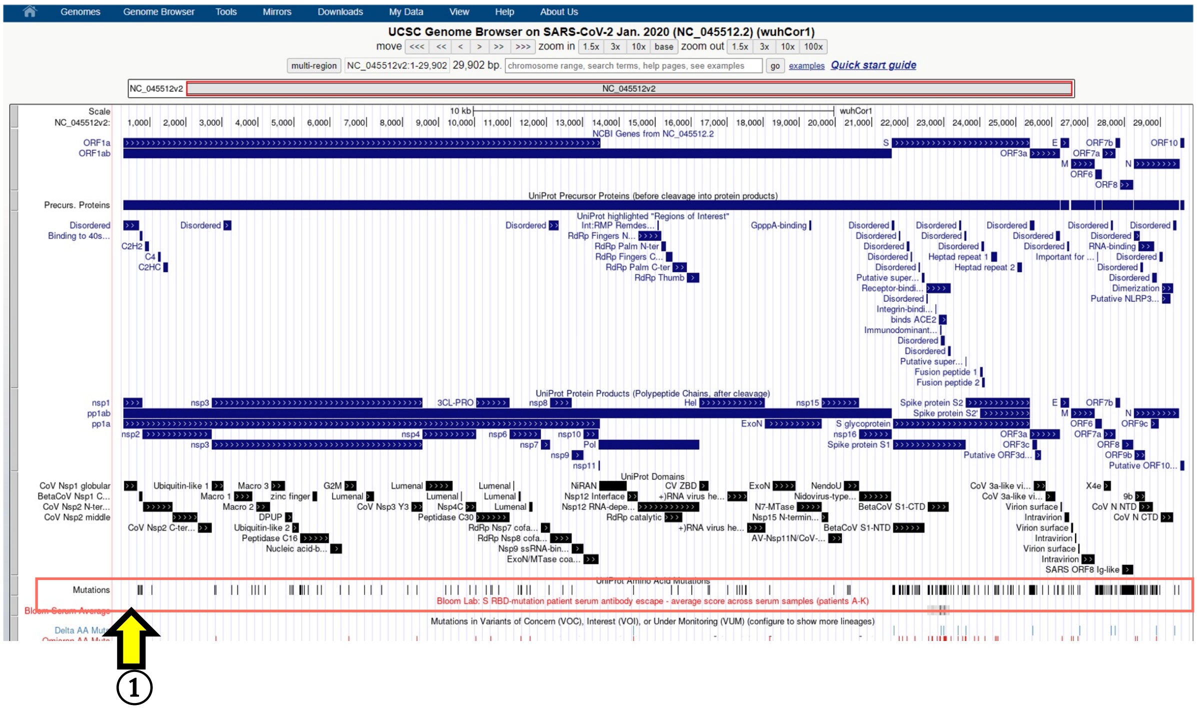

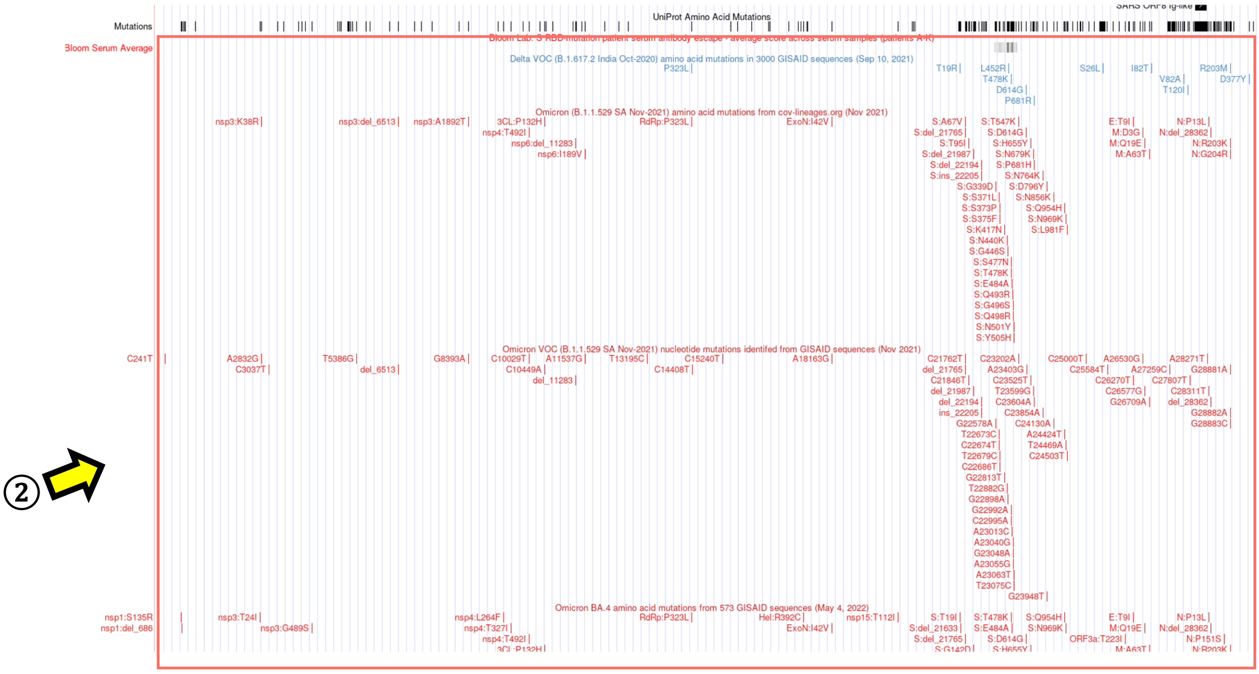

① In the bottom section of UCSC Genome Browser, select mutation
② Mutation data of SARS-CoV-2 genes provided

UCSC **COVID-19 Research at UCSC**

Main functions: Visualization

COVID-19 and Lung gene expression data in the UCSC Cell Browser:

COVID-19 Datasets:

- Bronchoalveolar Immune Cells in COVID-19 ①
- COVID-19 Airways ②
- COVID-19 and Influenza Immunophenotyping
- COVID-19 Cell Atlas
- COVID-19 SARS-CoV-2 and coronavirus-associated receptors and factors (SCARFs) ④
- HCA-LungMAP COVID-19 Integrated Analysis ⑤

Other Lung datasets in the Cell Browser

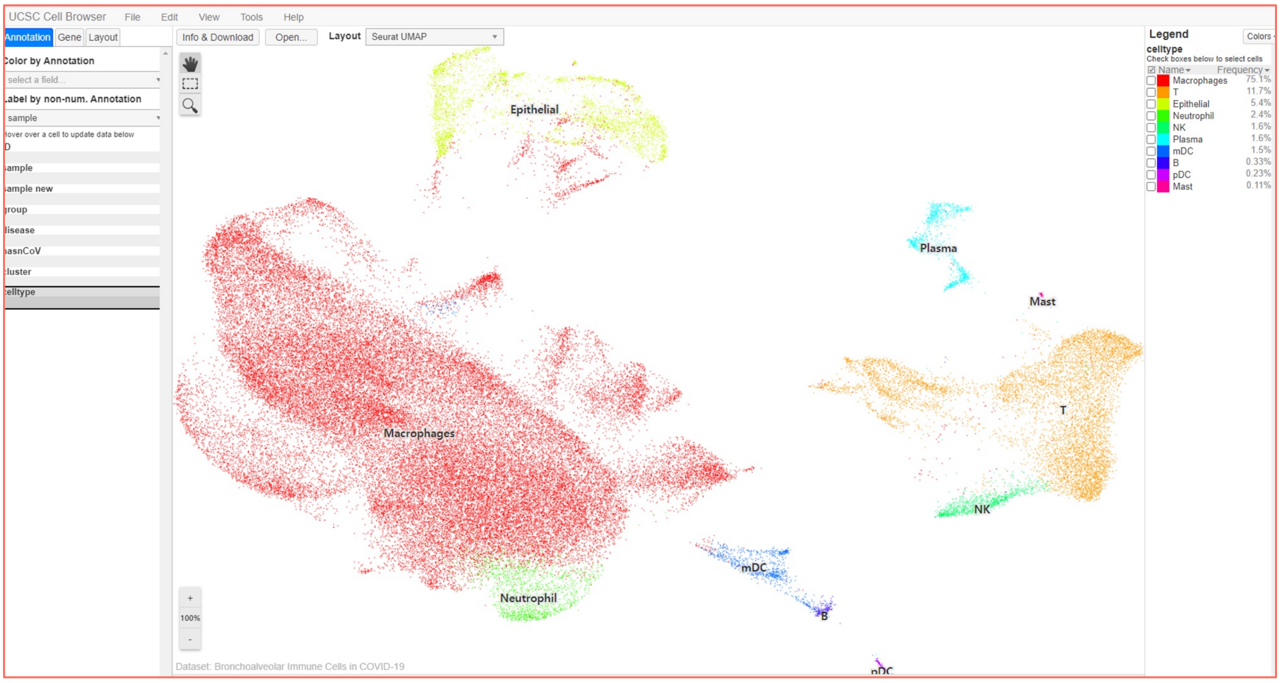

①-1

① **Select Bronchoalveolar Immune Cells in COVID-19**
①**-1 Check Single cell expression data**

Main functions: Visualization

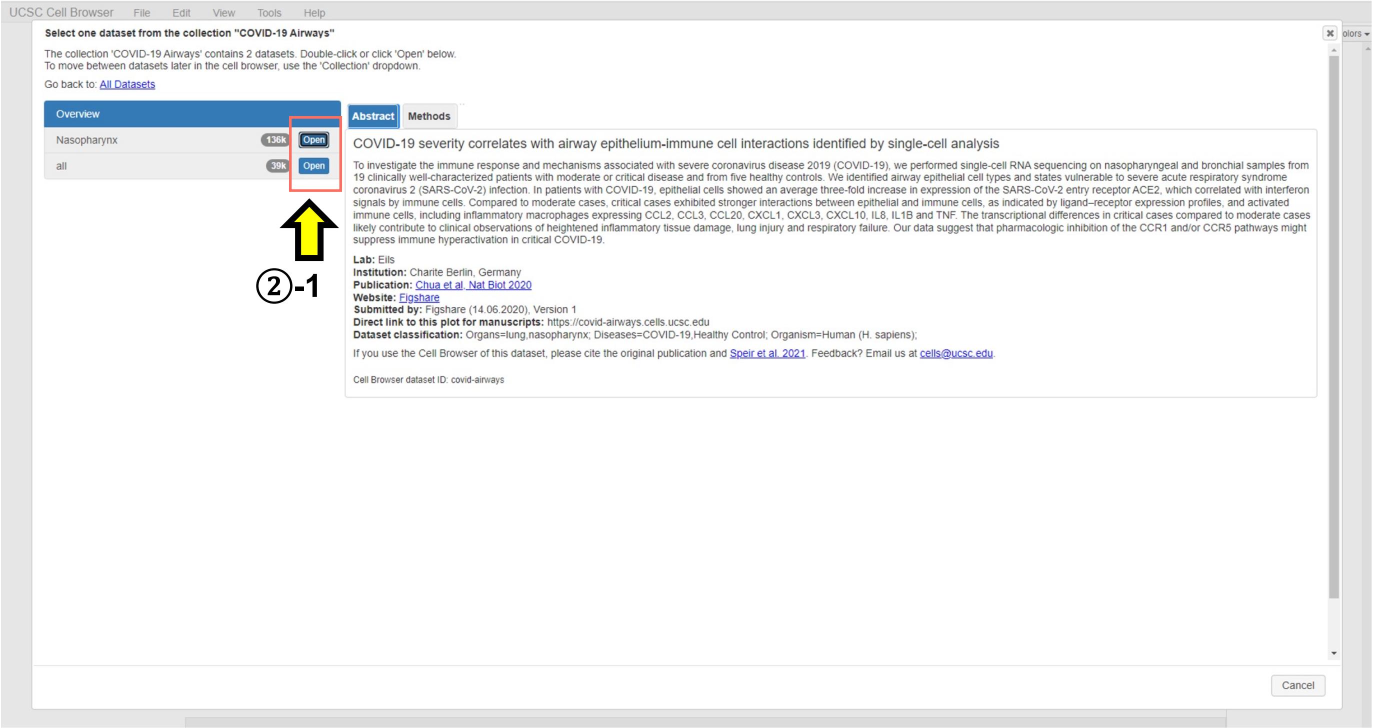

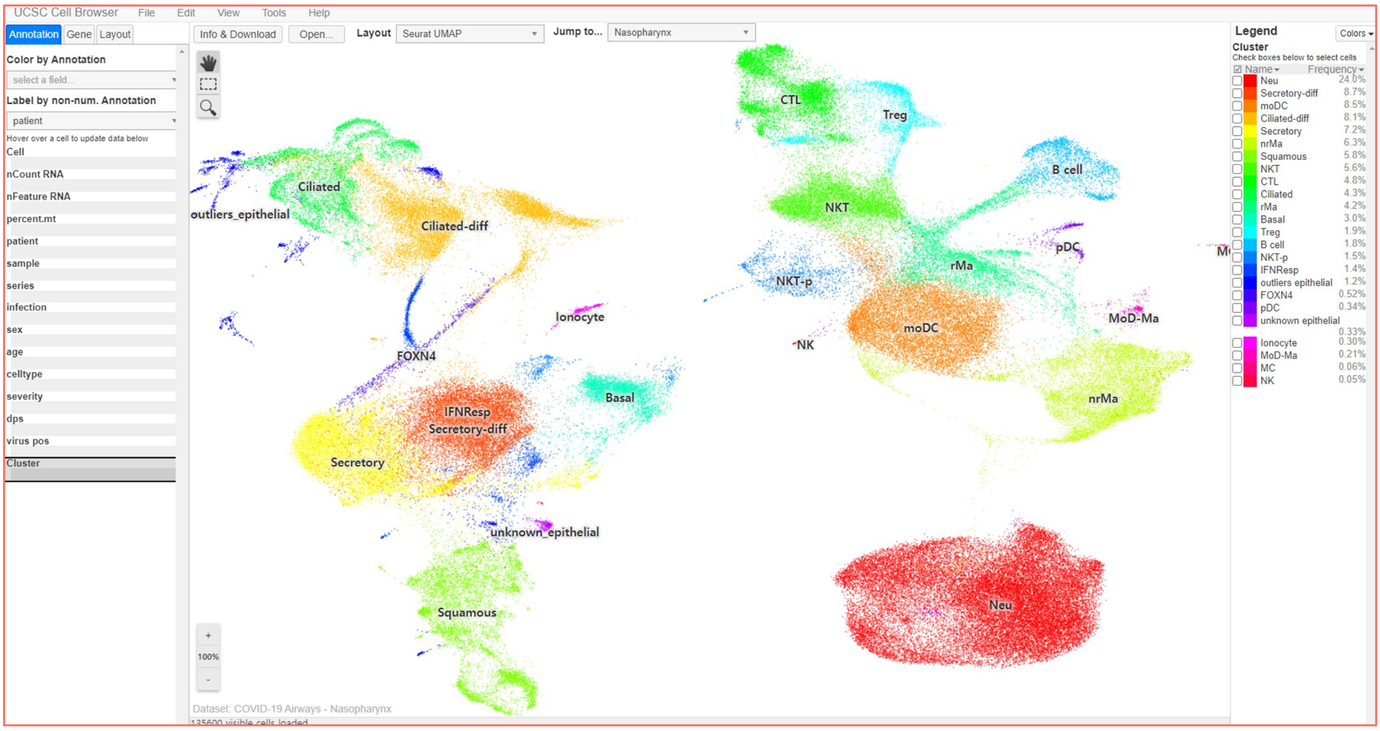

②-2

② Select COVID-19 Airways,
②-1 After selecting Overview, open click
②-2 Check Single cell expression data

COVID-19 Research at UCSC

Main functions: Visualization

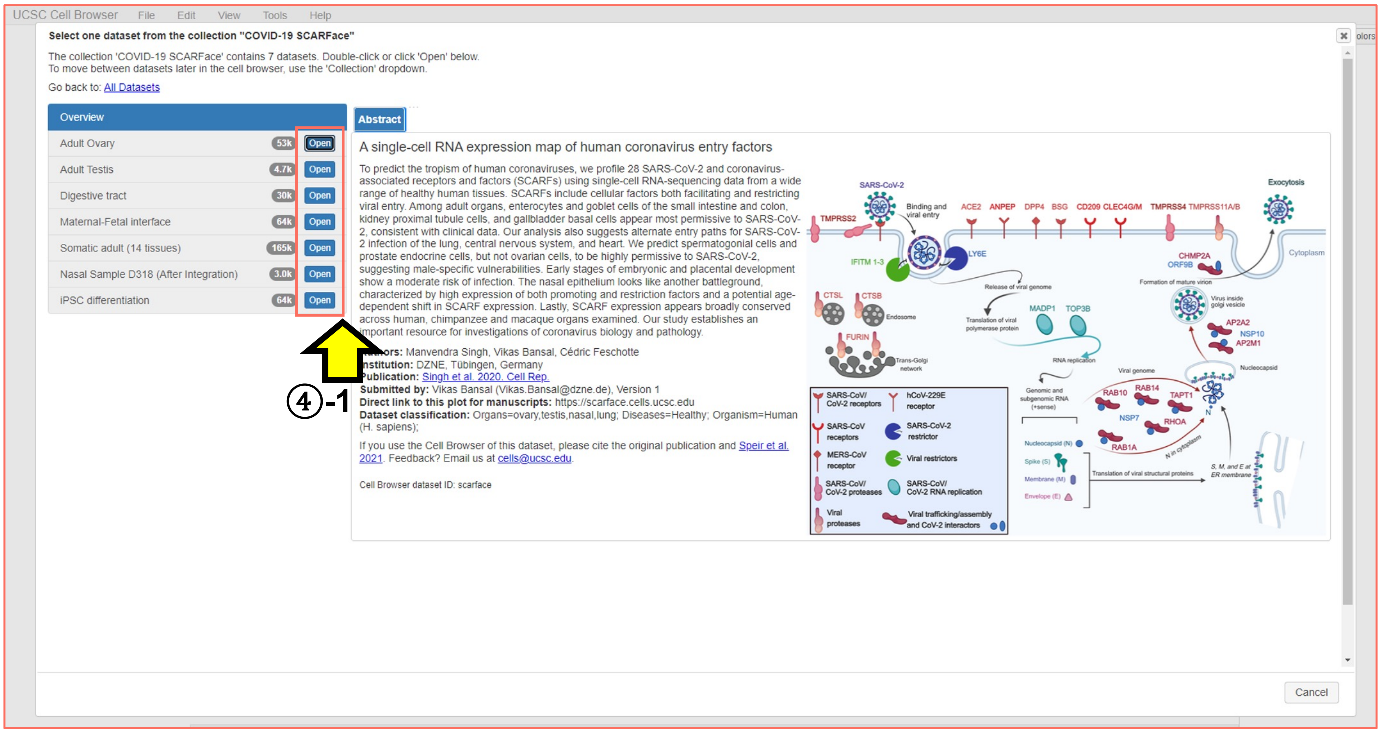

④-1

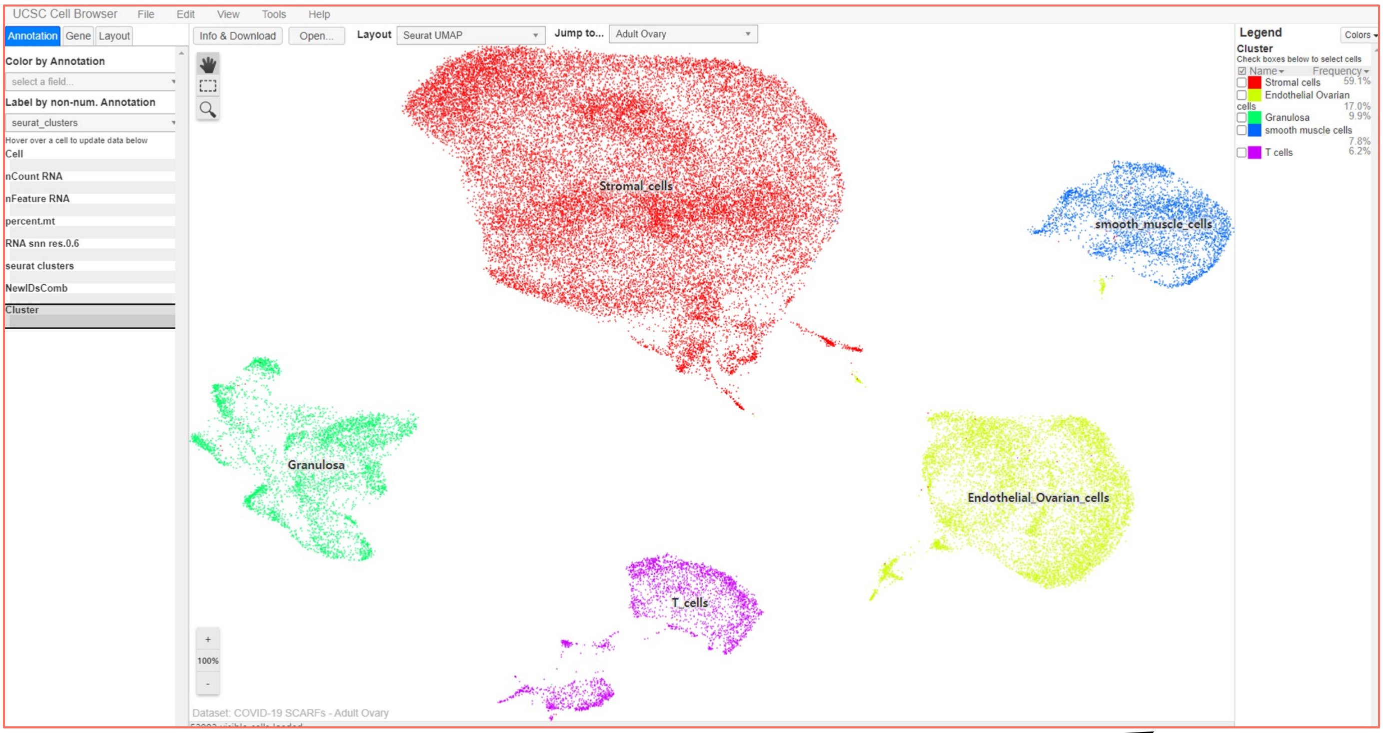

④-2

④ Select COVID-19 SARS-CoV-2 and coronavirus-associated receptors and factors (SCARFs),
④-1 After selecting Overview, open click
④-2 Check Single cell expression data

COVID-19 Research at UCSC

Main functions: Visualization

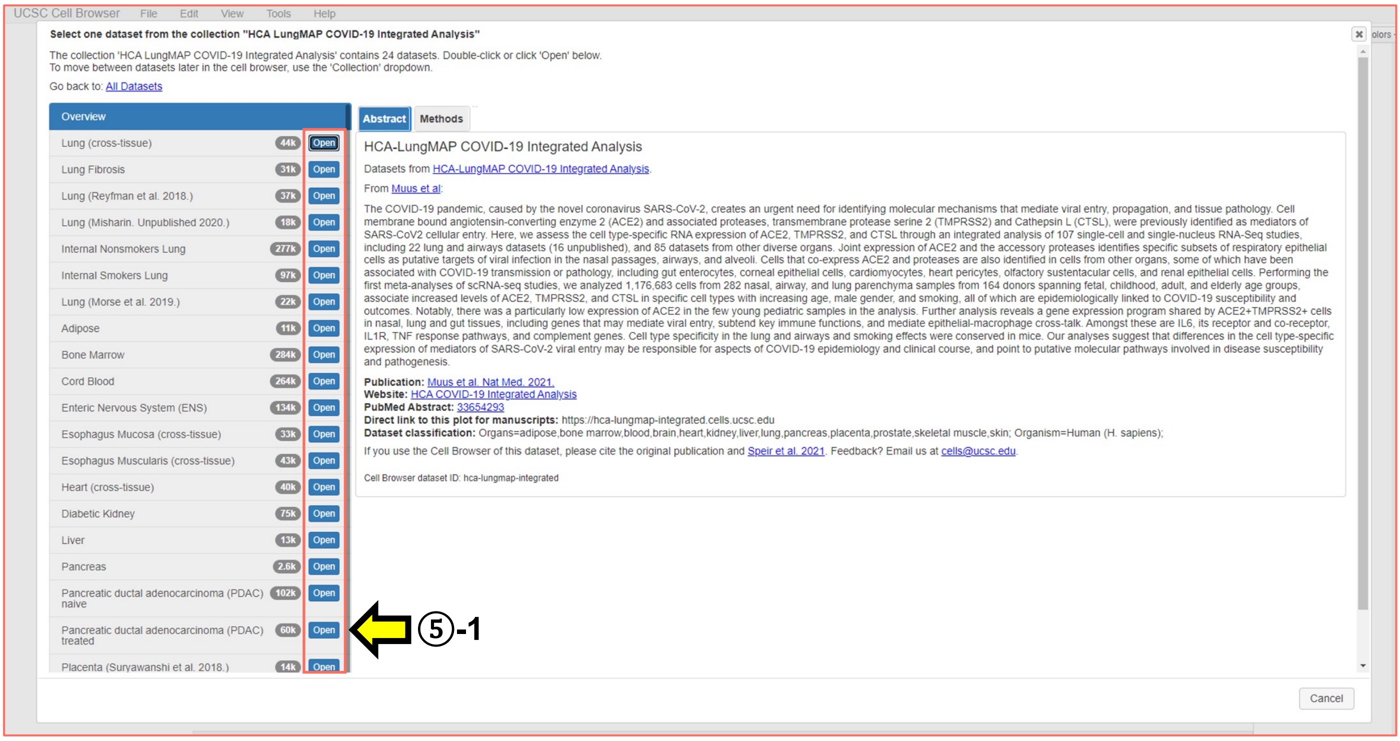

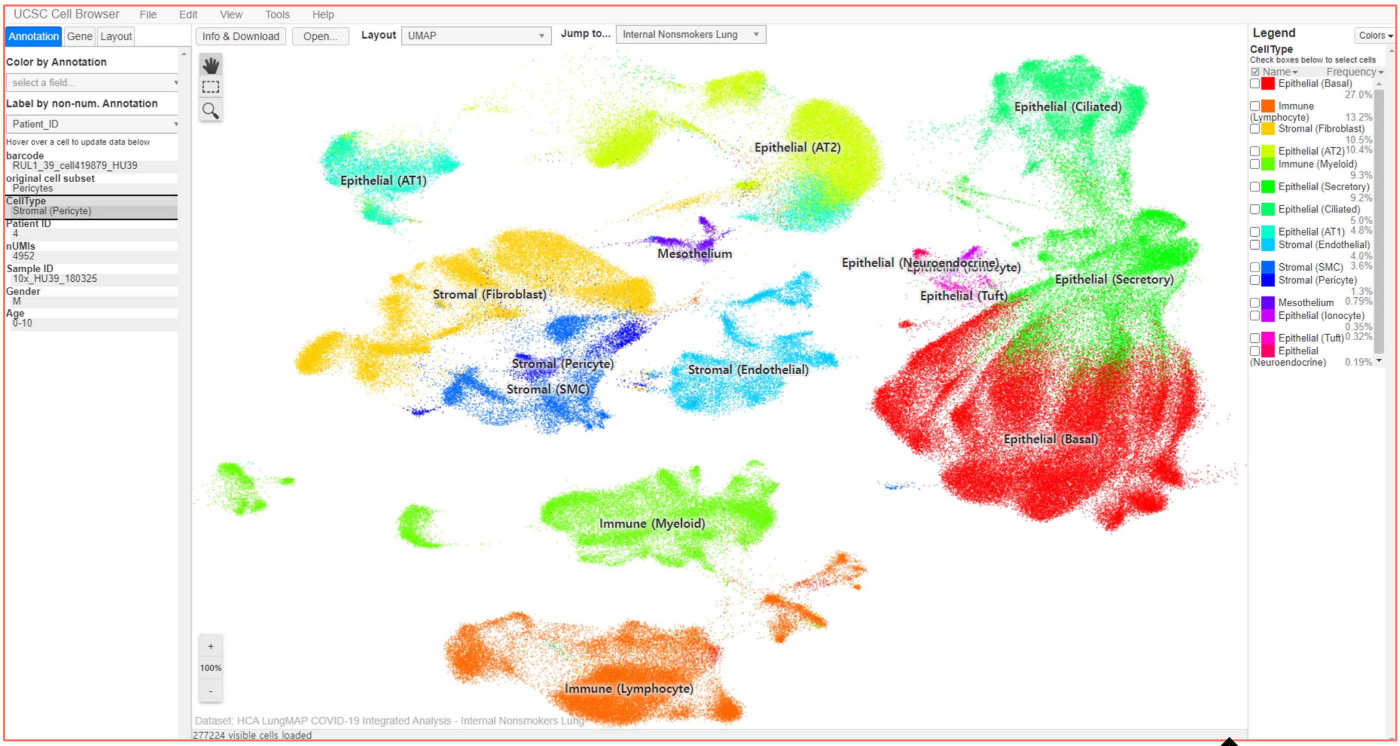

⑤-2

⑤ **Select HCA-LungMAP COVID-19 Integrated Analysis,**
⑤-1 **After selecting Overview, open click**
⑤-2 **Check Single cell expression data**

Main functions: Data analysis tool

UShER for real-time genomic contact tracing

In addition to the Genome Browser, we offer a **web interface** to **Ultrafast Sample placement on Existing tRees (UShER) (Turakhia et al.)**, a tool for identifying the relationships among a user's newly sequenced viral genomes and all known SARS-CoV-2 virus genomes. UShER identifies relationships between viral genomes by adding them to an existing phylogenetic tree of similar sequences that visually depicts the evolutionary relationships among the genome sequences. This approach empowers "genomic contact tracing". That is, UShER tells you whether virus genomes are closely related and therefore possibly from the same source, or if they are distantly related and the infections come from distinct sources. When newly sequenced virus genomes are added to a comprehensive tree of previously sequenced genomes, contact tracers are often able to determine where in the world those genomes came from. UShER is the only available method that can do this in "real time". Our tool places genomes onto a comprehensive global phylogeny of more than 80,000 virus genomes in less than one second. More information about UShER can be found on the UCSC news article, **New tools enable rapid analysis of coronavirus sequences and tracking of variants**. The number of genome sequences available has increased to 5.6 million since the article was initially published with 1.2 million sequences on May 2021.

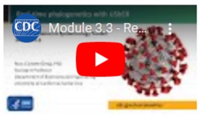

The **CDC COVID-19 Genomic Epidemiology Toolkit** now includes a training module for UShER! Module 3.3 includes a **video**, slides (**PDF**), and links to more resources.

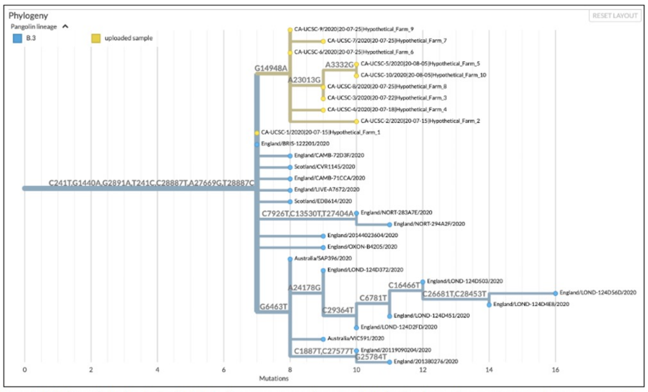

Example UShER results displayed using Nextstrain. Sequences representing a hypothetical outbreak are yellow; previously sampled sequences are blue. Branches are labeled by nucleotide mutations.

Genomes | Genome Browser | Tools | Mirrors | Downloads | My Data | Projects | Help | About Us

UShER: Ultrafast Sample placement on Existing tRee

Place your sequences in a global phylogenetic tree

Choose your pathogen: [SARS-CoV-2]

Select your FASTA, VCF or list of sequence names/IDs: [파일 선택] 선택된 파일 없음

or paste in sequence names/IDs:

Phylogenetic tree version:
[6,475,733 genomes from GenBank, COG-UK and CNCB (2022-11-21); sarscov2phylo 13-11-20 tree with newer sequences added by UShER]

Number of samples per subtree showing sample placement: [50]

[Upload] [Upload Example File] More example files

More information

Upload your SARS-CoV-2 sequence (FASTA or VCF file) to find the most similar complete, high-coverage samples from GISAID or from public sequence databases (INSDC: GenBank/ENA/DDBJ accessed using NCBI Virus, COG-UK and the China National Center for Bioinformation), and your sequence's placement in the phylogenetic tree generated by the sarscov2phylo pipeline. Placement is performed by Ultrafast Sample placement on Existing tRee (UShER) (Turakhia *et al.*). UShER also generates local subtrees to show samples in the context of the most closely related sequences. The subtrees can be visualized as Genome Browser custom tracks and/or using Nextstrain's interactive display which supports drag-and-drop of local metadata that remains on your computer.

GISAID data displayed in the Genome Browser are subject to GISAID's Terms and Conditions. SARS-CoV-2 genome sequences and metadata are available for download from GISAID EpiCoV™.

COVID-19 Pandemic Resources at UCSC

Privacy and sharing

Please do not upload Protected Health Information (PHI).

If even virus sequence files must remain local on your computer, then you can try ShUShER which runs entirely in your web browser so that no files leave your computer.

https://genome.ucsc.edu/cgi-bin/hgPhyloPlace

Tool that draws phylogenetic tree from uploaded SARS-CoV-2 sequence (FASTA or VCF file)

Dock CoV-2

Main page

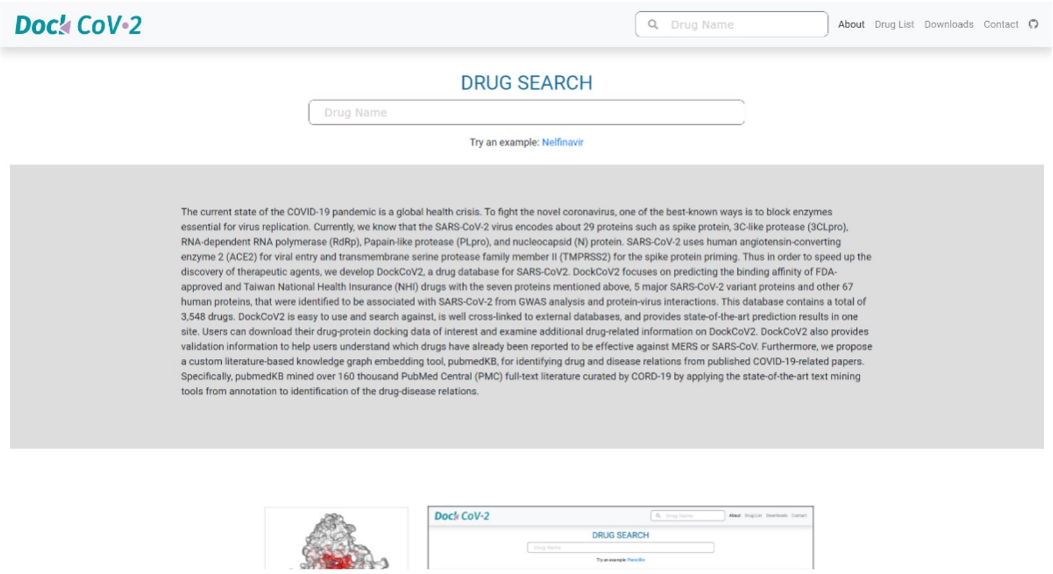

https://covirus.cc/drugs/

Dock CoV2 is a website that measures SARS-CoV-2 protein and protein drug interaction to provide to users.

Main functions: Visualization

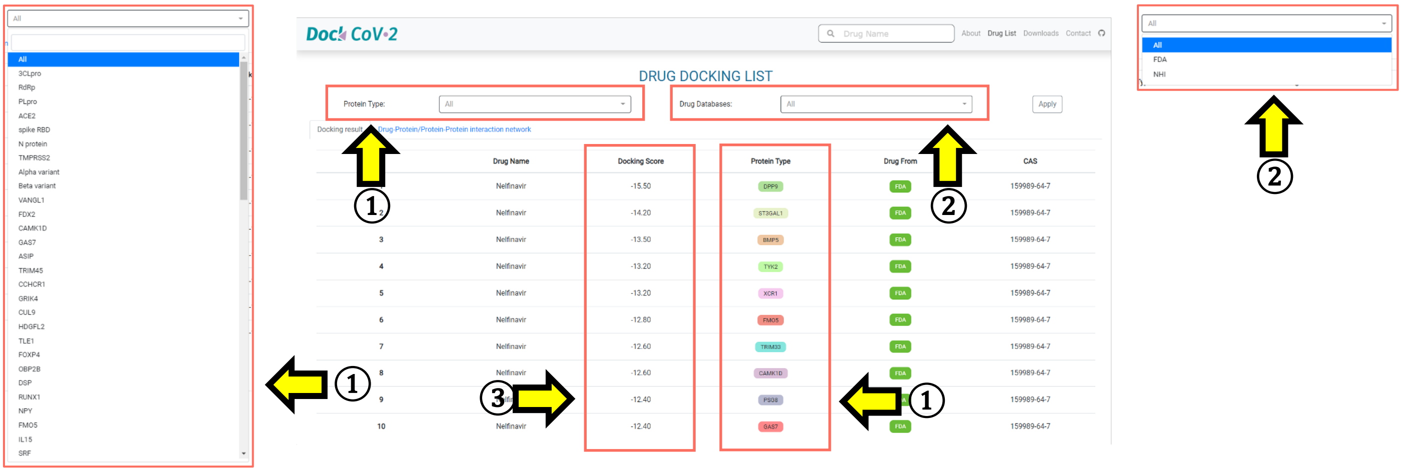

Drug docking list
① Protein type: SARS-CoV-2 protein ist, protein list, VOC variant list
② Drug Database: FDA-approved drugs, Taiwan National Health Insurance (NHI) drugs
③ Docking Score: Protein binding affinity

Dock CoV-2

Main functions: Visualization, Protein structure

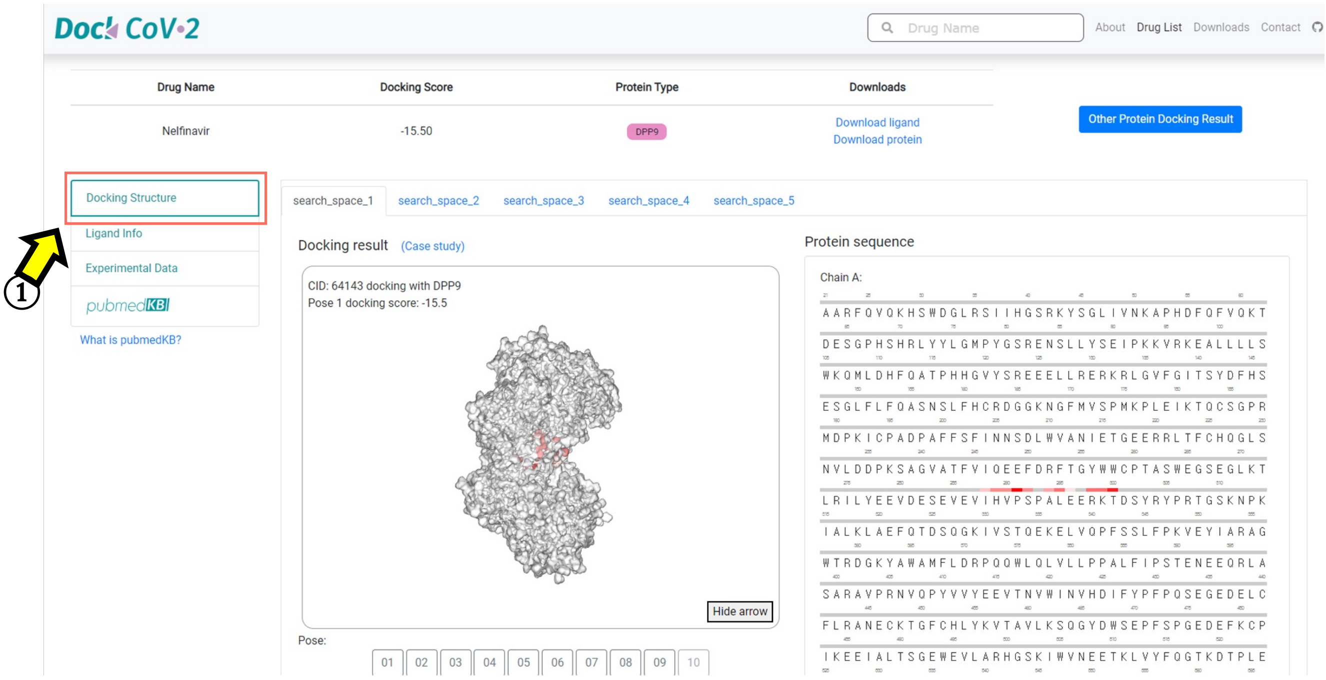

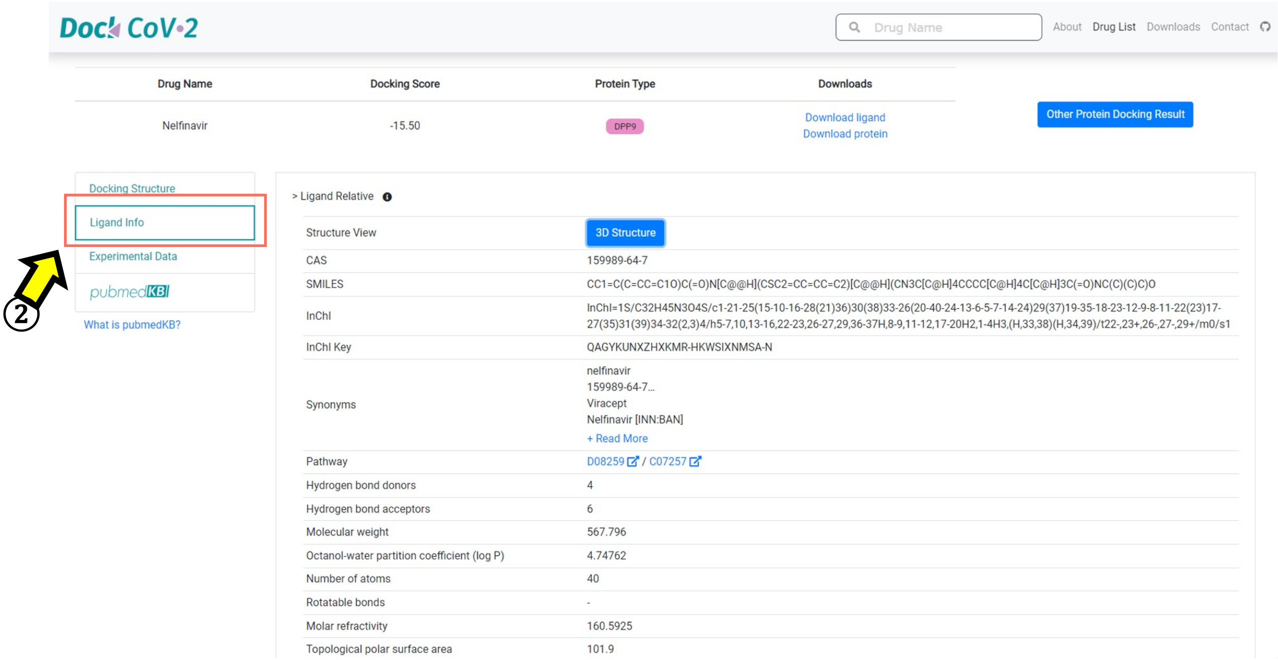

① Docking Structure: Docking result, protein 3D structure, protein sequence

② Ligand info: Ligand Relative, Clinical Relative, Druglikeness, Other Relative Info, Drug Similarity

Dock CoV-2

Main functions: Visualization, Treatment (clinical trials, drug)

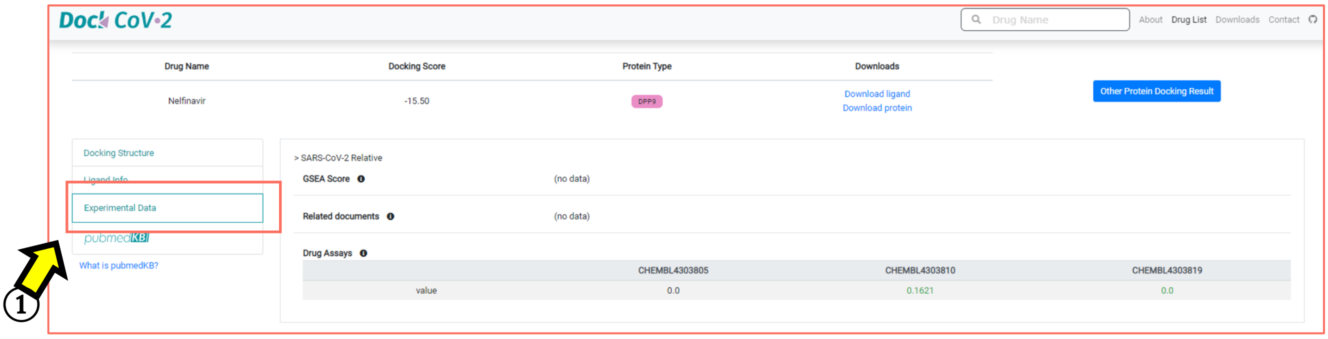

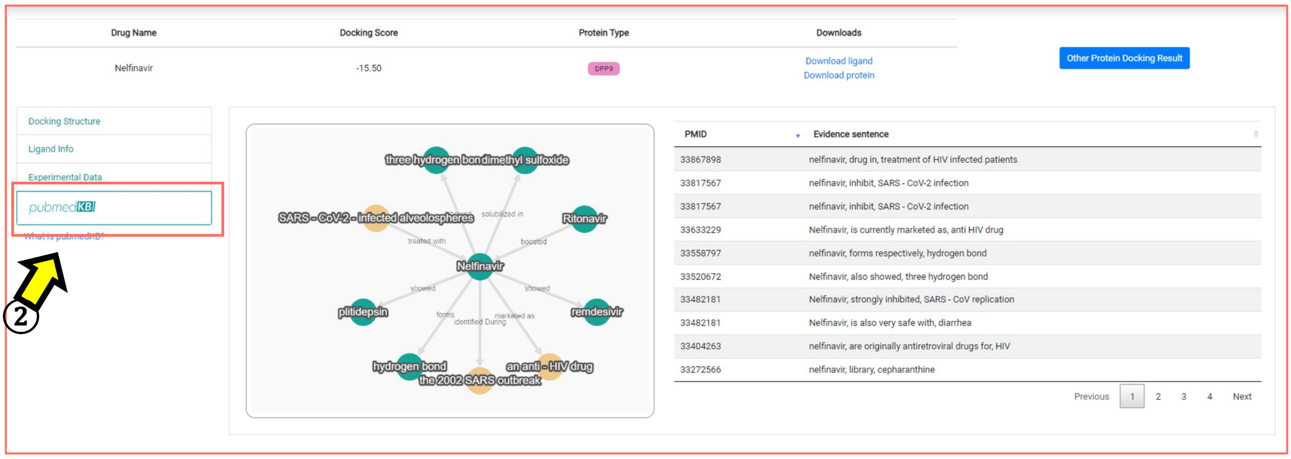

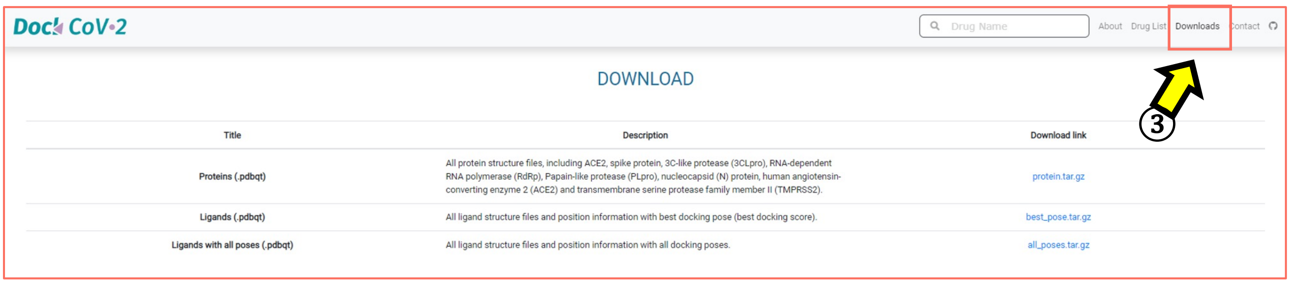

① Experimental Data: GSEA Score, Related documents, Drug Assays
② PubmedKB: Natural language processing service that provides custom literature-based knowledge graphs. Displays drug-disease networks.
③ Download: protein, ligands, Ligands with all poses

Our World in Data

Main page

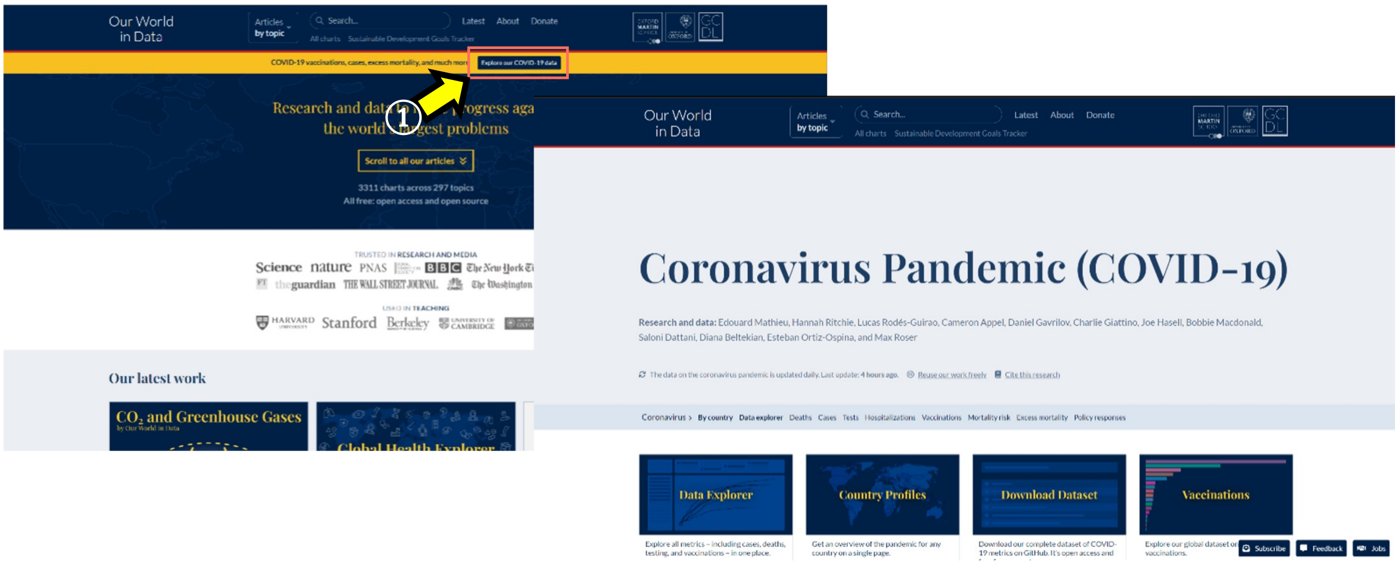

https://ourworldindata.org/
Our World in Data is a scientific online publication that deals with global issues such as disease, hunger, poverty, etc. Currently, the website compiles global data and studies related to the COVID-19 pandemic.
① Click on Explore our COVID-19 data

Main functions: Epidemiological data, Visualization

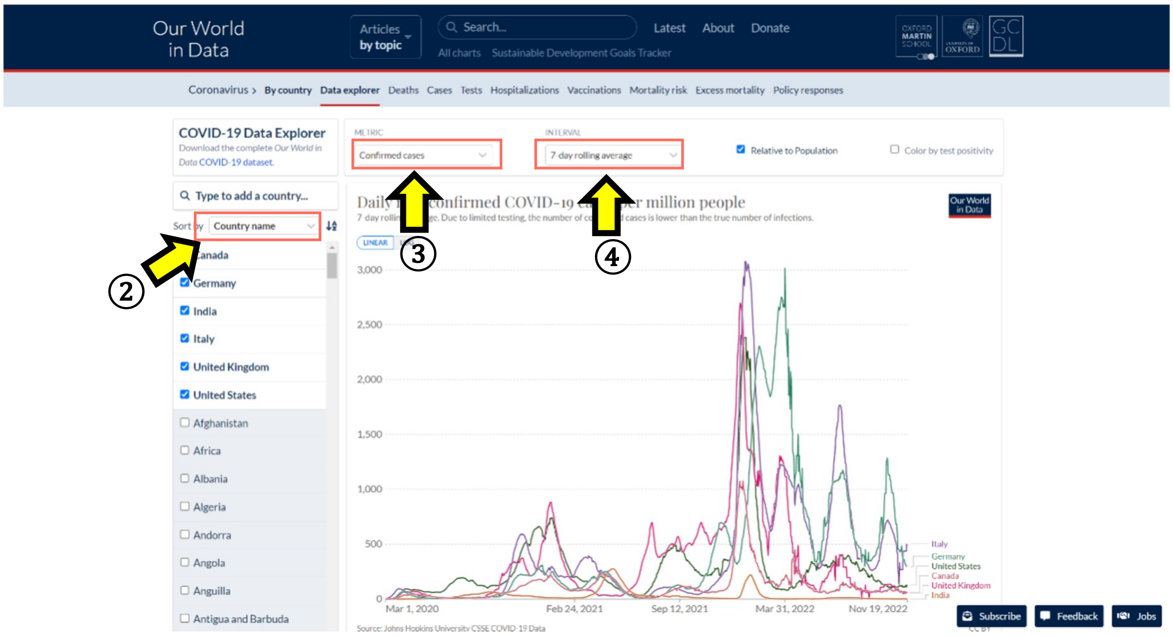

Options for chart creation and visualization
② Sort by: country select
③ METRIC
④ INTERVAL

Our World in Data

Main functions: Epidemiological data, Visualization

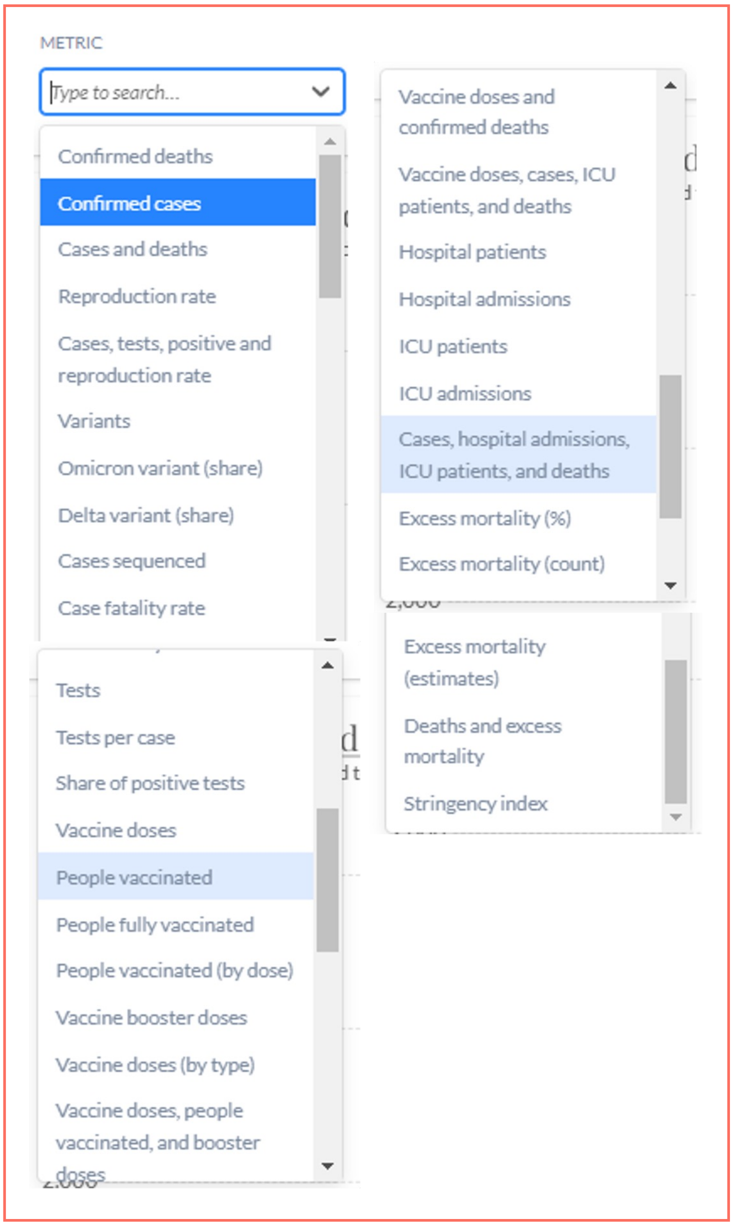

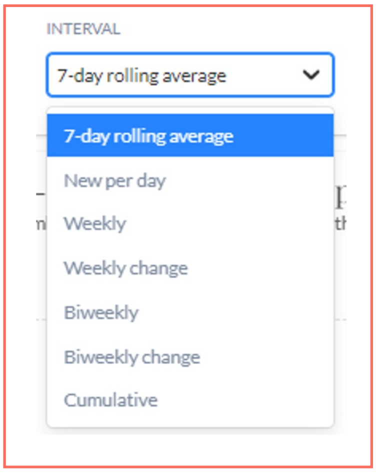

③-1

②-1

②-1: METRIC list
③-1: INTERVAL list

Our World in Data

Main functions: Epidemiological data, Visualization

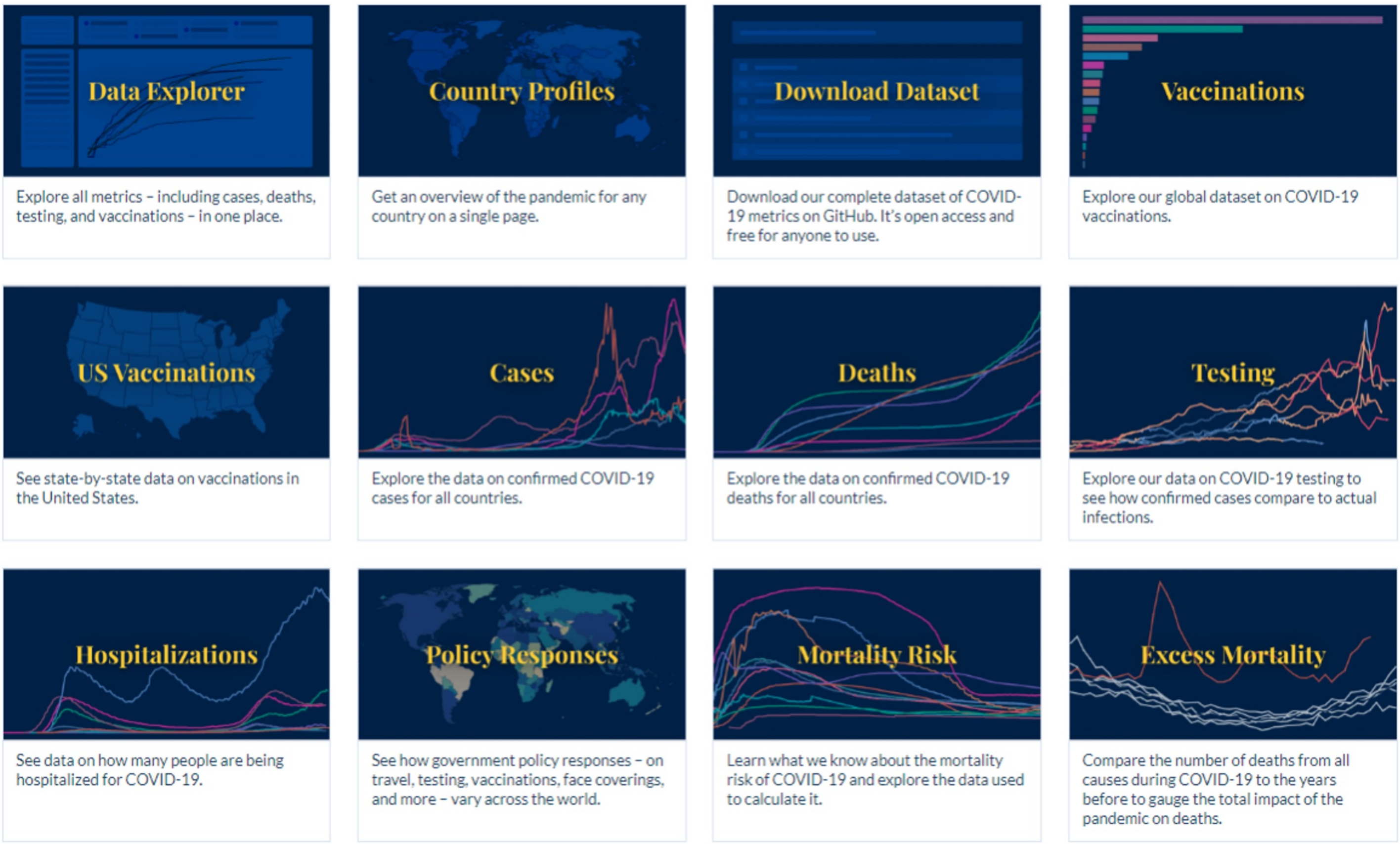

Coronavirus Pandemic (COVID-19) related data

- ① Data Explorer
- ② Country Profiles
- ③ Download Dataset
- ④ Vaccinations
- ⑤ US Vaccinations
- ⑥ Cases
- ⑦ Deaths
- ⑧ Testing
- ⑨ Hospitalizations : specific queries included
- ⑩ Policy Responses: specific queries included
- ⑪ Mortality Risk
- ⑫ Excess Mortality

JOHNS HOPKINS UNIVERSITY & MEDICINE | CORONAVIRUS RESOURCE CENTER

Main page

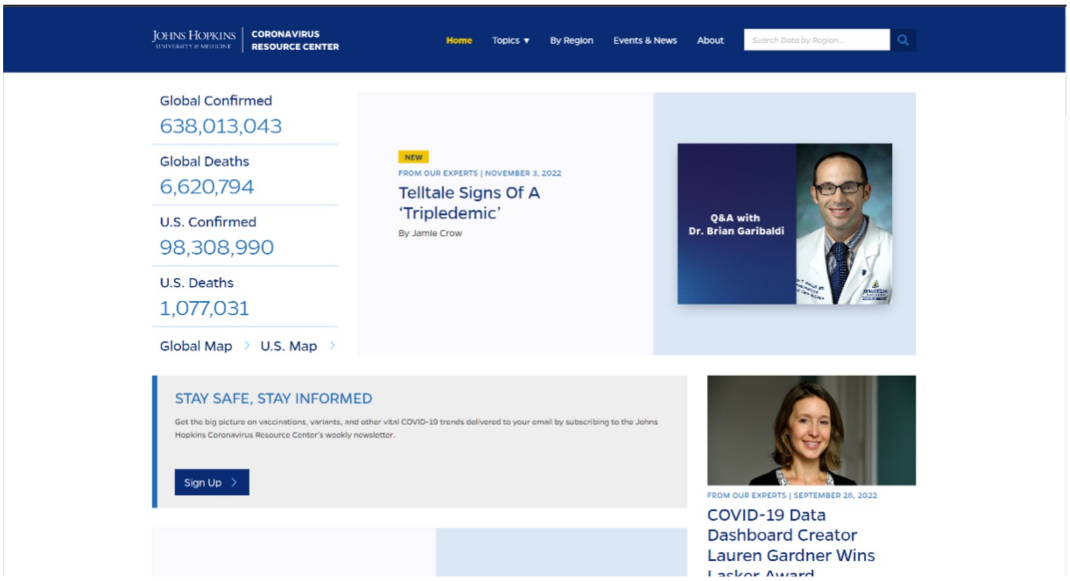

https://coronavirus.jhu.edu/

Johns Hopkins university provides COVID-19 epidemiological data (global, U.S.)

Main functions: Epidemiological data, Visualization

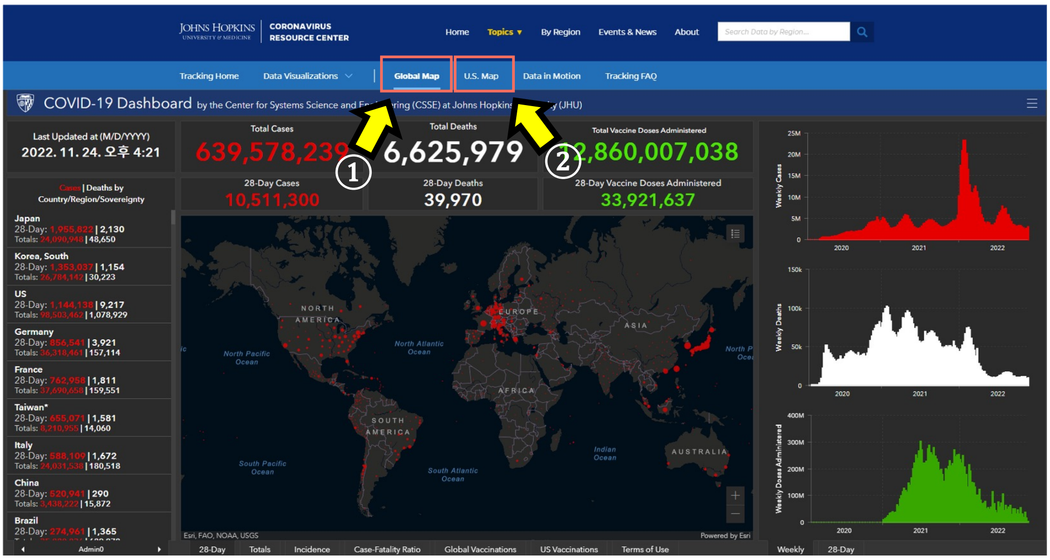

Johns Hopkins COVID-19 Testing Dashboard
① Global Map
② U.S Map

Main functions: Epidemiological data, Visualization

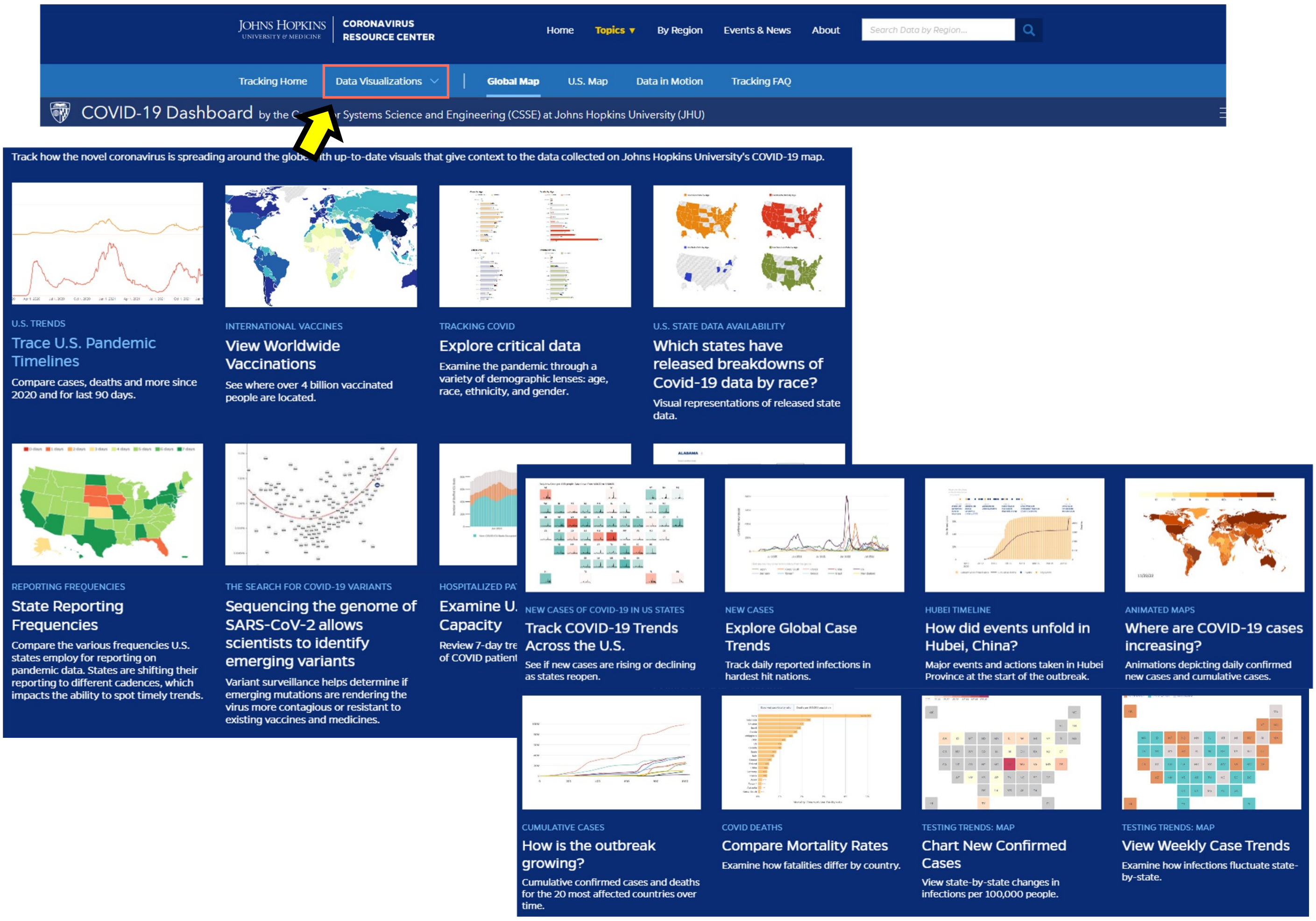

➡ **Click on Data Visualization**
Tracking Critical Data list

① Trace U.S. Pandemic Timelines
② View Worldwide Vaccinations
③ Explore critical data
④ Which states have released breakdowns of Covid-19 data by race?
⑤ State Reporting Frequencies
⑥ Sequencing the genome of SARS-CoV-2 allows scientists to identify emerging variants
⑦ Examine U.S. Hospital Capacity
⑧ Timeline of COVID-19 policies, cases, and deaths in your state
⑨ Track COVID-19 Trends Across the U.S.
⑩ Explore Global Case Trends
⑪ How did events unfold in Hubei, China?
⑫ Where are COVID-19 cases increasing?
⑬ How is the outbreak growing?
⑭ Compare Mortality Rates
⑮ Chart New Confirmed Cases
⑯ View Weekly Case Trends

Main page

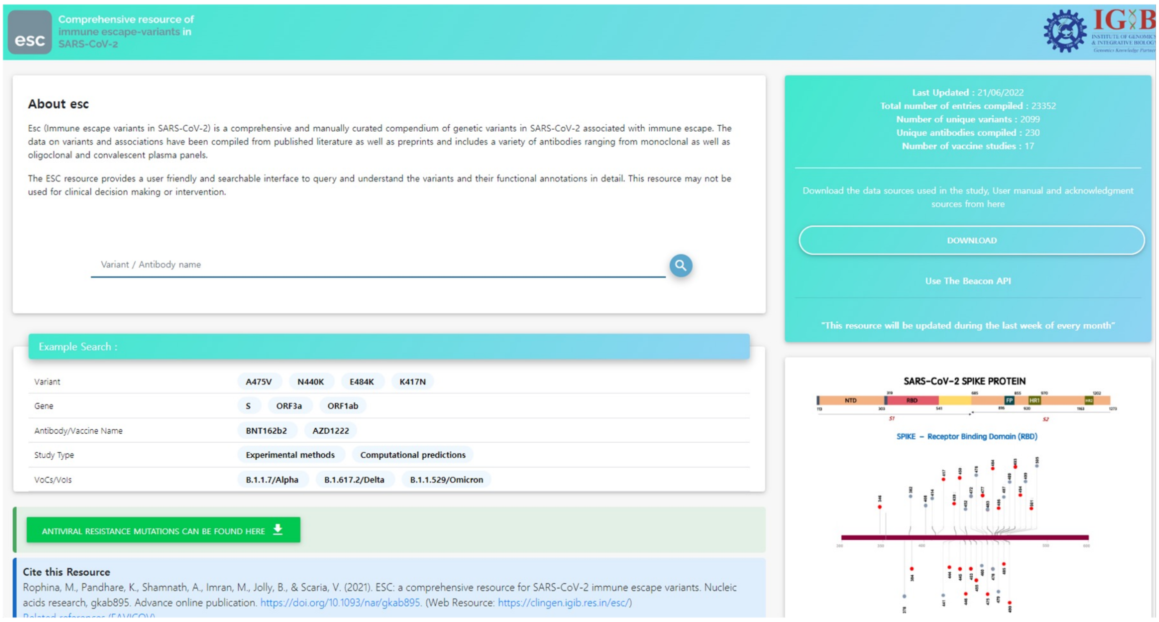

https://clingen.igib.res.in/esc/

Immune escape variants in SARS-CoV-2 (ESC) provides various antibody and vaccine data for immune escape related SARS-CoV-2 genetic variants. But, should not be used for clinical decision making or intervention.

Main functions: Clade/variant/lineage

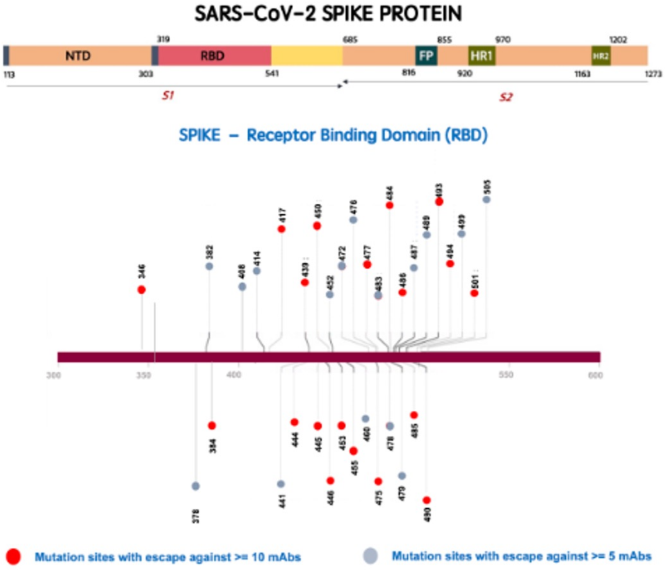

For spike protein, receptor binding domain (RBD) mutation data is provided.

esc Comprehensive resource of immune escape-variants in SARS-CoV-2

Main functions: Visualization, Treatment, Immunity

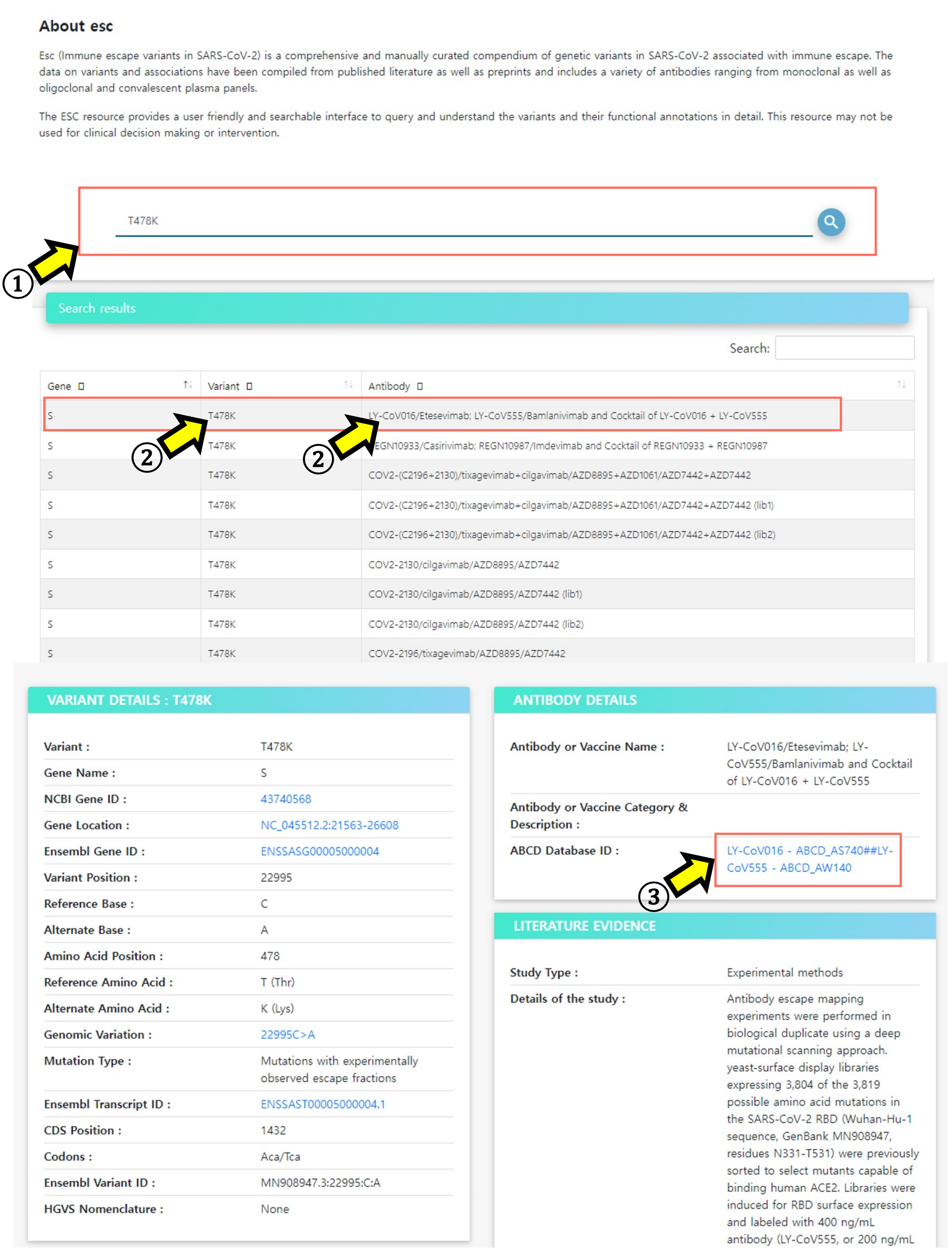

① In the search bar, enter mutation or antibody details. For example, T478K, a RBD mutation
② Antibody list provided for T478K mutation entered in search list. Click on mutation or antibody list.
③ Data provided for VARIANT DETAILS, ANTIBODY DETAILS, EPITOPE DETAILS, etc. Among those, click on ABCD Database ID in ANTIBODY DETAILS.

Main functions: Visualization, Treatment, Immunity

Expasy ABCD

Search | Clear

ABCD_AS740 in the ABCD (AntiBodies Chemically Defined) Database

Antigen information	
**Target type**	Protein
**Target link**	UniProt: P0DTC2 Severe acute respiratory syndrome coronavirus 2 (2019-nCoV) (SARS-CoV-2)
**Target name**	S, Spike protein, Spike glycoprotein
**Epitope**	ACE2 binding domain (residues 403-RGDEVRQ-409, 415-TGKIADY-421, 455-LFRKSN-460, 473-YQAGS-477, 484-EGFNCYFPLQSYGFQPTNGVGY-505)
**Antibody information**	
**Antibody name**	etesevimab
**Antibody synonyms**	anti-SARS-CoV-2 CB6, JS016, CB-6, JS-016, LY3832479, LY-CoV016
**Applications**	Flow cytometry, Neutralization, Surface plasmon resonance, Therapeutic, X-ray crystallography
**Cross-references**	IMGT/mAb-DB: 1107<br>PDB: 7C01
**Publications**	PMID: 32454512<br>PMID: 33475701

Antibody sequence

If you want to have the protein sequence of this antibody, please check the Publications and Cross-references links (a more comprehensive step-by-step guide on how to find sequences can be found here). If you have trouble finding it, just send us an email using the contact form.

Would you like to obtain this antibody?

It can be produced at the Geneva Antibody facility (for more information, please check here).

③-1

③-1 Clicking on ABCD Database ID provides antibody data through Expasy (Swiss Bioinformatics Resource Portal) and ABCD (Antibodies Chemically Defined).

esc Comprehensive resource of immune escape-variants in SARS-CoV-2

Main functions: Visualization, Treatment, Immunity

VARIANT DETAILS : T478K

Variant :	T478K
Gene Name :	S
NCBI Gene ID :	43740568
Gene Location :	NC_045512.2:21563-26608
Ensembl Gene ID :	ENSSASG00005000004
Variant Position :	22995
Reference Base :	C
Alternate Base :	A
Amino Acid Position :	478
Reference Amino Acid :	T (Thr)
Alternate Amino Acid :	K (Lys)
Genomic Variation :	22995C>A
Mutation Type :	Mutations with experimentally observed escape fractions
Ensembl Transcript ID :	ENSSAST00005000004.1
CDS Position :	1432
Codons :	Aca/Tca
Ensembl Variant ID :	MN908947.3:22995:C:A
HGVS Nomenclature :	None

VARIANTS OF CONCERN/INTEREST

VoCs/VoIs :	B.1.617.2 (Delta)
VoCs/VoIs Aliases :	21A/S:478K
VoCs/VoIs Description :	Potential reduction in neutralization by some EUA monoclonal antibody treatments. Potential reduction in neutralization by post-vaccination sera

PROTEIN DOMAIN DETAILS

PROTEIN NAME :	Spike glycoprotein
UNIPROT PROTEIN ID :	P0DTC2
PROTEIN LENGTH :	1273 amino acids
Protein Description :	Spike protein is one of the structural proteins of SARS-CoV-2. The monomeric protein consists of one large ectodomain, a single-pass transmembrane anchor, and a short intracellular tail at C-terminus. It encompasses 22 glycosylation sites. S protein cleaves into two subunits namely S1 and S2 following receptor recognition. Receptor Binding Domain (RBD) in S1 subunit plays a major role in ACE2 receptor binding.
Protein Domain :	Receptor Binding Domain (RBD) of SARS-CoV-2. The SARS-CoV-2 RBD has a twisted five-stranded antiparallel β sheet (β1, β2, β3, β4 and β7) with short connecting helices and loops that form the core. Between the β4 and β7 strands in the core, there is an extended insertion containing the short β5 and β6 strands, α4 and α5 helices

ANTIBODY DETAILS

Antibody or Vaccine Name :	LY-CoV016/Etesevimab; LY-CoV555/Bamlanivimab and Cocktail of LY-CoV016 + LY-CoV555
Antibody or Vaccine Category & Description :	
ABCD Database ID :	LY-CoV016 - ABCD_AS740##LY-CoV555 - ABCD_AW140

LITERATURE EVIDENCE

Study Type :	Experimental methods
Details of the study :	Antibody escape mapping experiments were performed in biological duplicate using a deep mutational scanning approach. yeast-surface display libraries expressing 3,804 of the 3,819 possible amino acid mutations in the SARS-CoV-2 RBD (Wuhan-Hu-1 sequence, GenBank MN908947, residues N331-T531) were previously sorted to select mutants capable of binding human ACE2. Libraries were induced for RBD surface expression and labeled with 400 ng/mL antibody (LY-CoV555, or 200 ng/mL each of LY-CoV555 and LY-CoV016 for 400 ng/mL total antibody). Cells were then incubated with 1:200 PE-conjugated goat anti-human-IgG (Jackson ImmunoResearch 109-115-098) to label for bound antibody and 1:100 FITC-conjugated anti-Myc (Immunology Consultants Lab CYMC-45F) to label for RBD surface expression. Yeast expressing the unmutated SARS-CoV-2 RBD were prepared in parallel to library samples and labeled at 400 ng/mL and 4 ng/mL with the corresponding antibody/cocktail for setting selection gates.
Experiment Type :	Mutations in isolation
Neutralization Quantification :	LY-CoV016-0.001044;LY-CoV555+LY-CoV016-0.001392;LY-CoV555-0.001778
Antibody Generation :	The LY-CoV555 antibody variable domain sequences were acquired from the LY-CoV555 crystal structure file PDB: 7KMG Purified antibody was produced by Genscript as human IgG in HD 293F mammalian cells Affinity Purification by RoboColumn Eshmuno A 0.6mL columns
SARS-CoV-2 Mutants Generation :	Yeast-surface display libraries expressing 3,804 of the 3,819 possible amino acid mutations in the SARS-CoV-2 RBD (Wuhan-Hu-1 sequence, GenBank MN908947, residues N331-T531) Libraries were induced for RBD surface expression and labeled with 400 ng/mL antibody (LY-CoV555, or 200 ng/mL each of LY-CoV555 and LY-CoV016 for 400 ng/mL total antibody)
Antibody Binding escape Profile :	Deep mutational scanning approach

Result list
VARIANT DETAILS, ANTIBODY DETAILS, LITERATURE EVIDENCE, FUNCTIONAL ANNOTATION, VARIANT FREQUENCY, VARIANTS OF CONCERN/ INTEREST, PROTEIN DOMAIN DETAILS, EPITOPE DETAILS, REFERENCES

Comprehensive resource of immune escape-variants in SARS-CoV-2

Main functions: Visualization, Treatment, Immunity

	insertion containing the short β5 and β6 strands, α4 and α5 helices and loops.
**Domain Position :**	SARS-CoV-2 RBD (residues Arg319–Phe541)
**Function of the Domain :**	Receptor Binding Domain is a vital immunogenic fragment in the Spike protein of SARS-CoV-2 virus that binds to specific endogenous receptors in the host. SARS-CoV-2 gains entry and causes infection in humans by binding to ACE2 receptor.

EPITOPE DETAILS

**Epitope Type :**	Linear peptide
**IEDB Reference B cell Epitope Sequence :**	FERDISTEIYQAGSTPCNGV (464-483)<br>ERDISTEIYQAGSTPCNG (465-482)<br>YQAGSTPCNGVEGFNCYF (473-490)<br>QAGSTPCNGVEGFNCYFPLQ (474-493)
**IEDB B cell Epitope ID :**	1309468<br>1087499<br>1087808<br>1309554
**IEDB B cell Antigen Accession :**	QII57161.1<br>QHD43416.1<br>QHD43416.1<br>QII57161.1
**IEDB Reference T cell epitope Sequence :**	RDISTEIYQAGSTPC (466-480)<br>EIYQAGSTPCNGVEG (471-485)<br>AGSTPCNGVEGFNCY (475-489)<br>GSTPCNGVEGFNCYF (476-490)
**T cell Epitope ID :**	1310761<br>1310360<br>1069064<br>1310461
**IEDB T cell Antigen Accession :**	QKE11719.1<br>QKE11719.1<br>QHD43416.1<br>QKE11719.1

**Antibody Binding escape Profile :**	Deep mutational scanning approach Antibody-escape cells were selected via fluorescence-activated cell sorting (FACS) on a BD FACSAria II

FUNCTIONAL ANNOTATION

**RefGene Function :**	Protein Coding Region
**Variation Type :**	Nonsynonymous SNV
**SIFT Score :**	-2.14
**SIFT Prediction :**	-1.73682
**GERP :**	0
**PhyloP :**	0.87
**PhastCons :**	Tolerated
**Uniprot Domains :**	disulf_bond
**UNIPROT Disulphite Bond :**	BetaCoV_S1-CTD
**UNIPROT glyphos :**	NA
**UNIPROT Transmembrane :**	NA
**IEDB B-Cell Epitopes :**	
**IEDB CD4 Epitopes :**	NA
**IEDB cd4Epitope Score :**	NA
**IEDB CD8 Epitopes :**	NA
**IEDB cd8Epitope Score :**	NA
**MPDI Potential Immunogenic Regions :**	NA
**MPDI Potential Immunodominant Epitopes :**	NA
**ARTIC Primers :**	NA
**RT PCR Primers Probes :**	
**Sequencing Error Sites :**	NA
**Homoplasic Positions :**	NA
**Hypermutable Sites :**	NA

VARIANT FREQUENCY

**Global Variant Frequency :**	0.4459300766
**Variant Frequency by Geography :**	Africa(0.314129848330436)<br>Asia(0.363458011973464)<br>Europe(0.458930161992063)<br>NorthAmerica(0.460996516009646)<br>Oceania(0.440888464294244)<br>SouthAmerica(0.182057908338168)
**Variant Population genetics :**	None
**Variant Sample Genotype :**	None

REFERENCES

Starr, T. N., Greaney, A. J., Dingens, A. S., & Bloom, J. D. (n.d.). Complete map of SARS-CoV-2 RBD mutations that escape the monoclonal antibody LY-CoV555 and its cocktail with LY-CoV016. https://doi.org/10.1101/2021.02.17.431683

Result list

VARIANT DETAILS, ANTIBODY DETAILS, LITERATURE EVIDENCE, FUNCTIONAL ANNOTATION, VARIANT FREQUENCY, VARIANTS OF CONCERN/ INTEREST, PROTEIN DOMAIN DETAILS, EPITOPE DETAILS, REFERENCES

T-cell COVID-19 Atlas (T-CoV)

Browse how SARS-CoV-2 mutations affect CD8 and CD4 T-cell epitopes

Main page

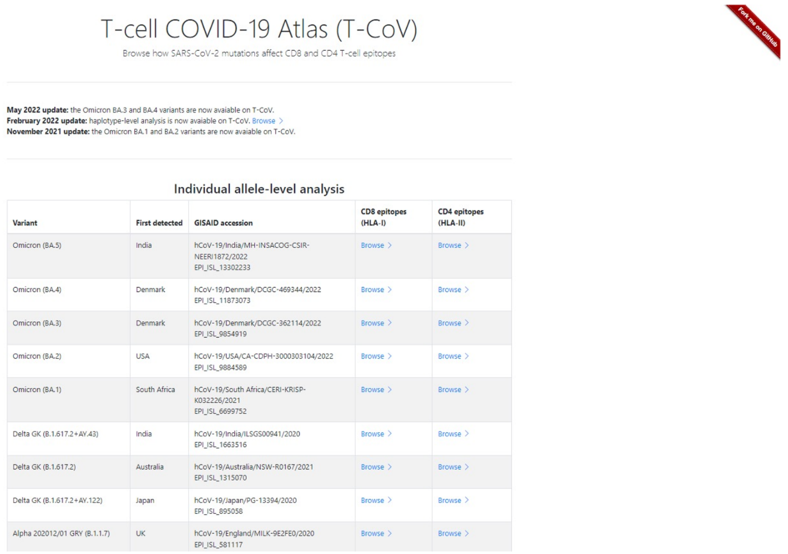

T-cell COVID-19 Atlas (T-CoV)

Browse how SARS-CoV-2 mutations affect CD8 and CD4 T-cell epitopes

May 2022 update: the Omicron BA.3 and BA.4 variants are now avaiable on T-CoV.
Frebruary 2022 update: haplotype-level analysis is now avaiable on T-CoV. Browse >
November 2021 update: the Omicron BA.1 and BA.2 variants are now avaiable on T-CoV.

Individual allele-level analysis

Variant	First detected	GISAID accession	CD8 epitopes (HLA-I)	CD4 epitopes (HLA-II)
Omicron (BA.5)	India	hCoV-19/India/MH-INSACOG-CSIR-NEERI1872/2022 EPI_ISL_13302233	Browse >	Browse >
Omicron (BA.4)	Denmark	hCoV-19/Denmark/DCGC-469344/2022 EPI_ISL_11873073	Browse >	Browse >
Omicron (BA.3)	Denmark	hCoV-19/Denmark/DCGC-362114/2022 EPI_ISL_9854919	Browse >	Browse >
Omicron (BA.2)	USA	hCoV-19/USA/CA-CDPH-3000303104/2022 EPI_ISL_9884589	Browse >	Browse >
Omicron (BA.1)	South Africa	hCoV-19/South Africa/CERI-KRISP-K032226/2021 EPI_ISL_6699752	Browse >	Browse >
Delta GK (B.1.617.2+AY.43)	India	hCoV-19/India/ILSGS00941/2020 EPI_ISL_1663516	Browse >	Browse >
Delta GK (B.1.617.2)	Australia	hCoV-19/Australia/NSW-R0167/2021 EPI_ISL_1315070	Browse >	Browse >
Delta GK (B.1.617.2+AY.122)	Japan	hCoV-19/Japan/PG-13394/2020 EPI_ISL_895058	Browse >	Browse >
Alpha 202012/01 GRY (B.1.1.7)	UK	hCoV-19/England/MILK-9E2FE0/2020 EPI_ISL_581117	Browse >	Browse >

https://t-cov.hse.ru/

T-CoV is a database that predicts binding affinities between peptides and HLA alleles for CD8 and CD4 epitope due to SARS-CoV-2 mutation.

Main functions: Clade/variant/lineage, Visualization, Immunity

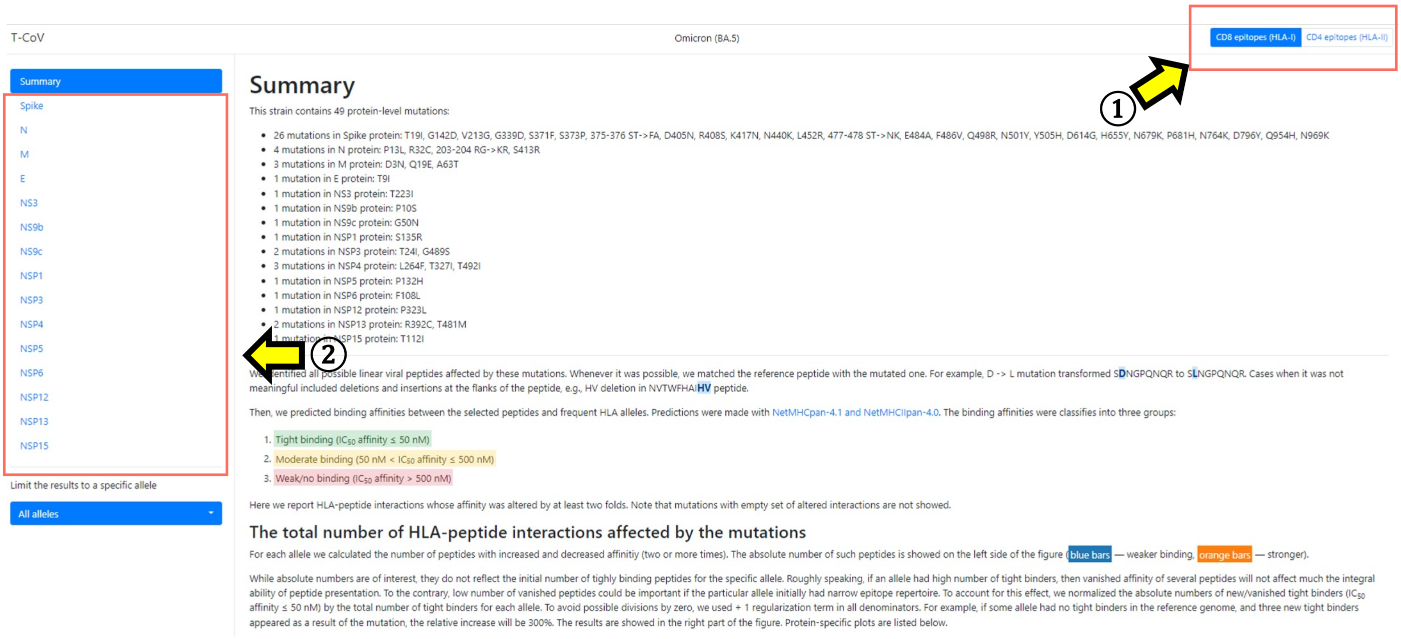

T-CoV

Omicron (BA.5)

CD8 epitopes (HLA-I) | CD4 epitopes (HLA-II)

Summary | Spike | N | M | E | NS3 | NS9b | NS9c | NSP1 | NSP3 | NSP4 | NSP5 | NSP6 | NSP12 | NSP13 | NSP15

Limit the results to a specific allele

All alleles

Summary

This strain contains 49 protein-level mutations:

- 26 mutations in Spike protein: T19I, G142D, V213G, G339D, S371F, S373P, 375-376 ST->FA, D405N, R408S, K417N, N440K, L452R, 477-478 ST->NK, E484A, F486V, Q498R, N501Y, Y505H, D614G, H655Y, N679K, P681H, N764K, D796Y, Q954H, N969K
- 4 mutations in N protein: P13L, R32C, 203-204 RG->KR, S413R
- 3 mutations in M protein: D3N, Q19E, A63T
- 1 mutation in E protein: T9I
- 1 mutation in NS3 protein: T223I
- 1 mutation in NS9b protein: P10S
- 1 mutation in NS9c protein: G50N
- 1 mutation in NSP1 protein: S135R
- 2 mutations in NSP3 protein: T24I, G489S
- 3 mutations in NSP4 protein: L264F, T327I, T492I
- 1 mutation in NSP5 protein: P132H
- 1 mutation in NSP6 protein: F108L
- 1 mutation in NSP12 protein: P323L
- 2 mutations in NSP13 protein: R392C, T481M
- 1 mutation in NSP15 protein: T112I

We identified all possible linear viral peptides affected by these mutations. Whenever it was possible, we matched the reference peptide with the mutated one. For example, D -> L mutation transformed S**D**NGPQNQR to S**L**NGPQNQR. Cases when it was not meaningful included deletions and insertions at the flanks of the peptide, e.g., HV deletion in NVTWFHAI**HV** peptide.

Then, we predicted binding affinities between the selected peptides and frequent HLA alleles. Predictions were made with NetMHCpan-4.1 and NetMHCIIpan-4.0. The binding affinities were classifies into three groups:

1. Tight binding (IC_{50} affinity ≤ 50 nM)
2. Moderate binding (50 nM < IC_{50} affinity ≤ 500 nM)
3. Weak/no binding (IC_{50} affinity > 500 nM)

Here we report HLA-peptide interactions whose affinity was altered by at least two folds. Note that mutations with empty set of altered interactions are not showed.

The total number of HLA-peptide interactions affected by the mutations

For each allele we calculated the number of peptides with increased and decreased affinitiy (two or more times). The absolute number of such peptides is showed on the left side of the figure (blue bars — weaker binding, orange bars — stronger).

While absolute numbers are of interest, they do not reflect the initial number of tighly binding peptides for the specific allele. Roughly speaking, if an allele had high number of tight binders, then vanished affinity of several peptides will not affect much the integral ability of peptide presentation. To the contrary, low number of vanished peptides could be important if the particular allele initially had narrow epitope repertoire. To account for this effect, we normalized the absolute numbers of new/vanished tight binders (IC_{50} affinity ≤ 50 nM) by the total number of tight binders for each allele. To avoid possible divisions by zero, we used + 1 regularization term in all denominators. For example, if some allele had no tight binders in the reference genome, and three new tight binders appeared as a result of the mutation, the relative increase will be 300%. The results are showed in the right part of the figure. Protein-specific plots are listed below.

① CD8 epitopes(HLA-I), CD4 epitopes(HLA-II) select
② SARS-CoV-2 protein structure selection available

T-cell COVID-19 Atlas (T-CoV)

Browse how SARS-CoV-2 mutations affect CD8 and CD4 T-cell epitopes

Main functions: Clade/variant/lineage, Visualization, Immunity

Spike protein

Download figure   Export data to csv

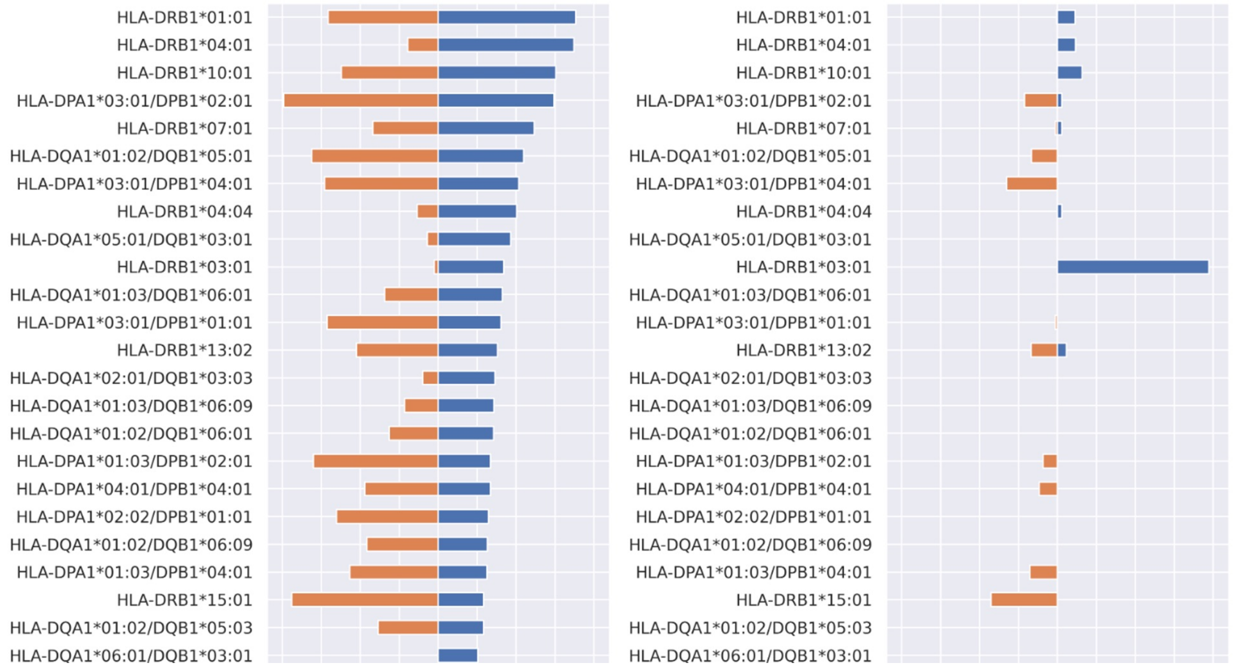

477-478 ST->NK

```
Reference   LYRLFRKSNLKPFERDISTEIYQAGSTPCNGVEGFNCYFPLQSYGFQPTNGV
            |||||||||||||||||||||||||  |||||||||||||||||||||||||
Mutated     RYRLFRKSNLKPFERDISTEIYQAGNKPCNGVAGVNCYFPLQSYGFRPTYGV
```

Export table to csv

Allele	Reference peptide	Mutated peptide	Reference affinity (IC_{50}, nM)	Mutated affinity (IC_{50}, nM)
HLA-DQA1*05:01/DQB1*03:01	STEIYQAGSTPCNGV	STEIYQAGNKPCNGV	91	1340
HLA-DQA1*05:01/DQB1*03:01	TEIYQAGSTPCNGVE	TEIYQAGNKPCNGVA	86	1108
HLA-DQA1*05:01/DQB1*03:01	EIYQAGSTPCNGVEG	EIYQAGNKPCNGVAG	99	1217
HLA-DQA1*05:01/DQB1*03:01	ISTEIYQAGSTPCNG	ISTEIYQAGNKPCNG	150	1810
HLA-DQA1*05:01/DQB1*03:01	STEIYQAGSTPCNGVEG	STEIYQAGNKPCNGVAG	148	1773
HLA-DQA1*05:01/DQB1*03:01	DISTEIYQAGSTPCNGV	DISTEIYQAGNKPCNGV	168	2000
HLA-DQA1*05:01/DQB1*03:01	GSTPCNGVEGFNCYF	GNKPCNGVAGVNCYF	3783	319
HLA-DQA1*05:01/DQB1*03:01	ISTEIYQAGSTPCNGVE	ISTEIYQAGNKPCNGVA	149	1686
HLA-DQA1*05:01/DQB1*03:01	ISTEIYQAGSTPCNGV	ISTEIYQAGNKPCNGV	125	1408
HLA-DQA1*06:01/DQB1*03:01	STEIYQAGSTPCNGV	STEIYQAGNKPCNGV	234	2602
HLA-DQA1*05:01/DQB1*03:01	TEIYQAGSTPCNGVEGF	TEIYQAGNKPCNGVAGV	158	1745
HLA-DQA1*05:01/DQB1*03:01	IYQAGSTPCNGVEGF	IYQAGNKPCNGVAGV	137	1502
HLA-DQA1*05:01/DQB1*03:01	TEIYQAGSTPCNGVEG	TEIYQAGNKPCNGVAG	120	1309
HLA-DQA1*05:01/DQB1*03:01	STEIYQAGSTPCNGVE	STEIYQAGNKPCNGVA	117	1273
HLA-DQA1*05:01/DQB1*03:01	STPCNGVEGFNCYFP	NKPCNGVAGVNCYFP	3849	363
HLA-DQA1*05:01/DQB1*03:01	EIYQAGSTPCNGVEGF	EIYQAGNKPCNGVAGV	139	1392

CD8 epitopes(HLA-I)
① HLA-peptide figure provided
② For reference and mutation, amino acid sequence and affinity comparison

T-cell COVID-19 Atlas (T-CoV)

Browse how SARS-CoV-2 mutations affect CD8 and CD4 T-cell epitopes

Main functions: Clade/variant/lineage, Visualization, Immunity

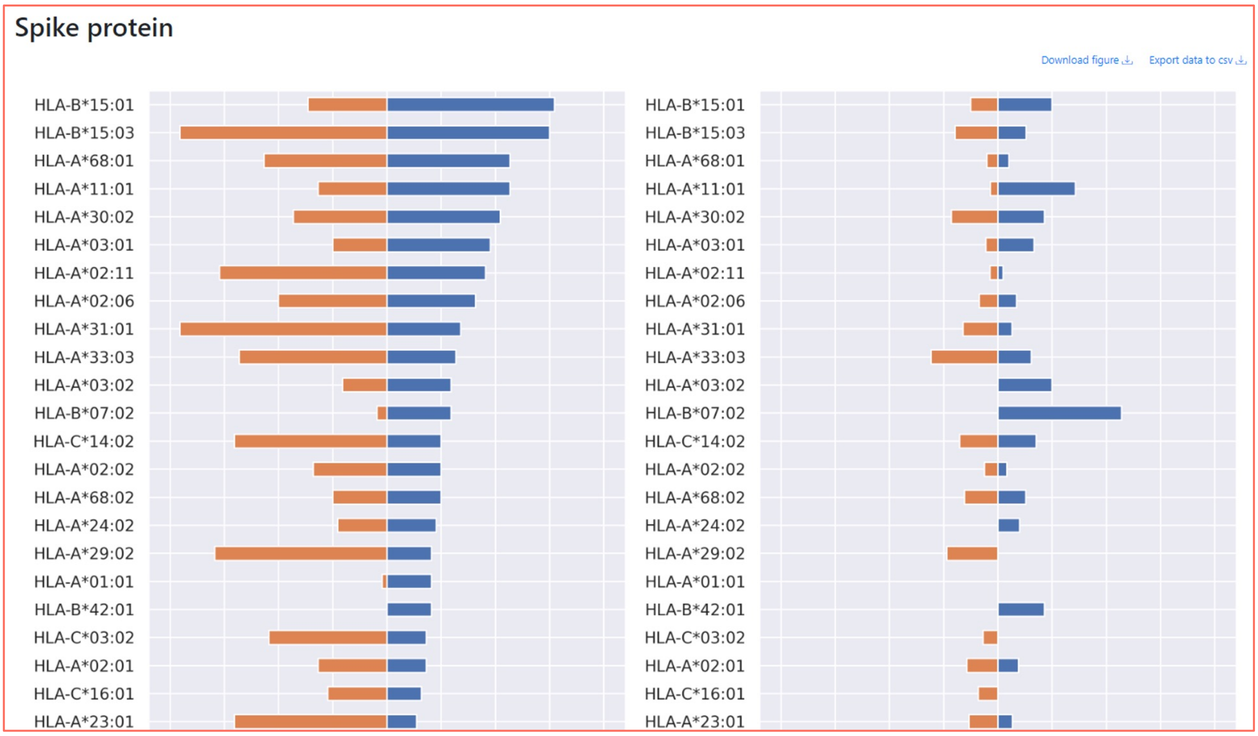

①

477-478 ST->NK

```
Reference   LYRLFRKSNLKPFERDISTEIYQAGSTPCNGVEGFNCYFPLQSYGFQPTNGV
            ||||||||||||||||||||||||||  ||||||||||||||||||||||||
Mutated     RYRLFRKSNLKPFERDISTEIYQAGNKPCNGVAGVNCYFPLQSYGFRPTYGV
```

Export table to csv

Allele	Reference peptide	Mutated peptide	Reference affinity (IC_{50}, nM)	Mutated affinity (IC_{50}, nM)
HLA-A*11:01	STEIYQAGST	STEIYQAGNK	30358	67
HLA-A*68:01	STEIYQAGST	STEIYQAGNK	22142	157
HLA-A*02:02	YQAGSTPCNGVEGF	YQAGNKPCNGVAGV	9447	68
HLA-A*03:01	STEIYQAGST	STEIYQAGNK	32241	308
HLA-A*02:11	YQAGSTPCNGVEGF	YQAGNKPCNGVAGV	17568	184
HLA-B*35:01	TPCNGVEGF	KPCNGVAGV	319	21824
HLA-A*02:06	YQAGSTPCNGVEGF	YQAGNKPCNGVAGV	5118	87
HLA-B*15:03	YQAGSTPCNGVEGF	YQAGNKPCNGVAGV	217	12581
HLA-B*15:01	YQAGSTPCNGVEGF	YQAGNKPCNGVAGV	408	12442
HLA-A*02:11	YQAGSTPCNGV	YQAGNKPCNGV	173	678
HLA-B*50:01	TEIYQAGSTP	TEIYQAGNKP	464	1807
HLA-A*02:06	YQAGSTPCNGV	YQAGNKPCNGV	39	146
HLA-A*02:01	YQAGSTPCNGV	YQAGNKPCNGV	420	1490
HLA-C*14:02	IYQAGSTPC	IYQAGNKPC	134	459
HLA-A*02:02	YQAGSTPCNGV	YQAGNKPCNGV	107	233

②

CD4 epitopes(HLA-II)
① HLA-peptide figure provided
② For reference and mutation, amino acid sequence and affinity comparison

CovInter

Main page

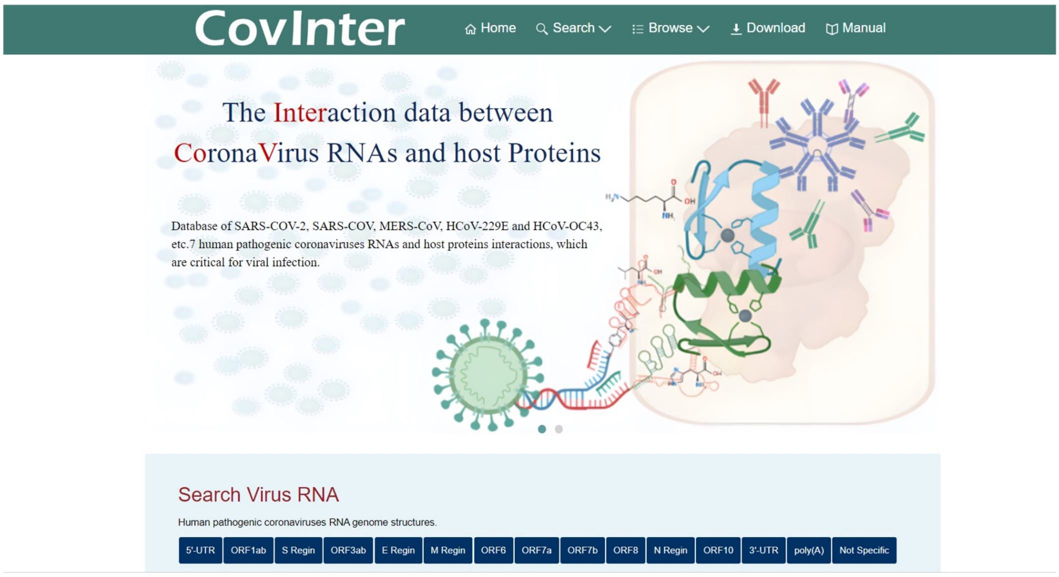

http://covrpii.idrblab.net/

A database of interactions between SARS-CoV-2 RNAs and host proteins

Main functions: Genome browser (sequence)

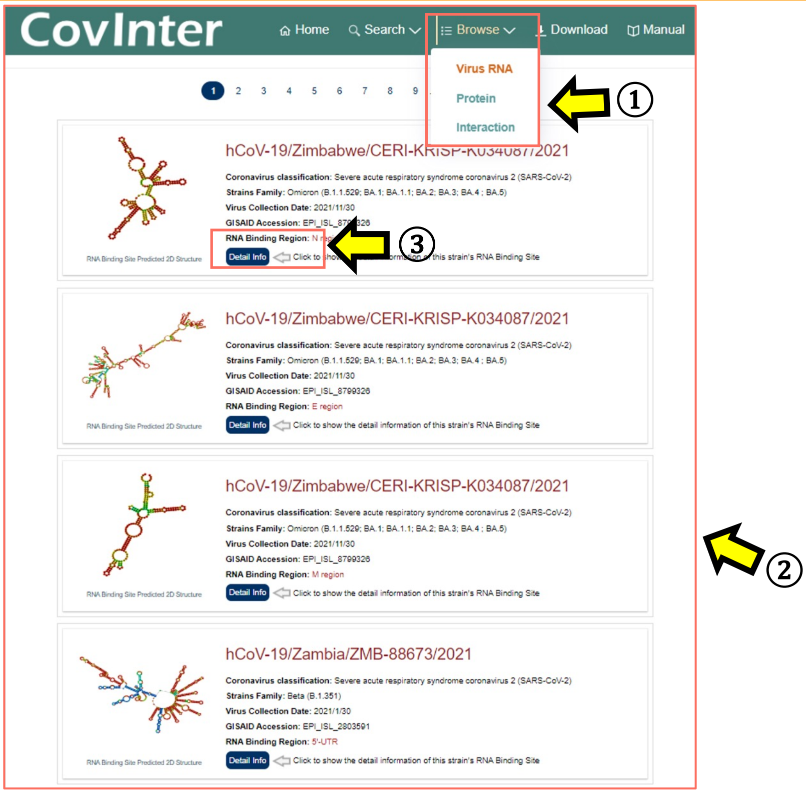

① Through Browse, searching for virus RNA, protein , interaction possible
② virus RNA
③ Click on Detail Info

Main functions: Visualization, Data analysis tool, treatment

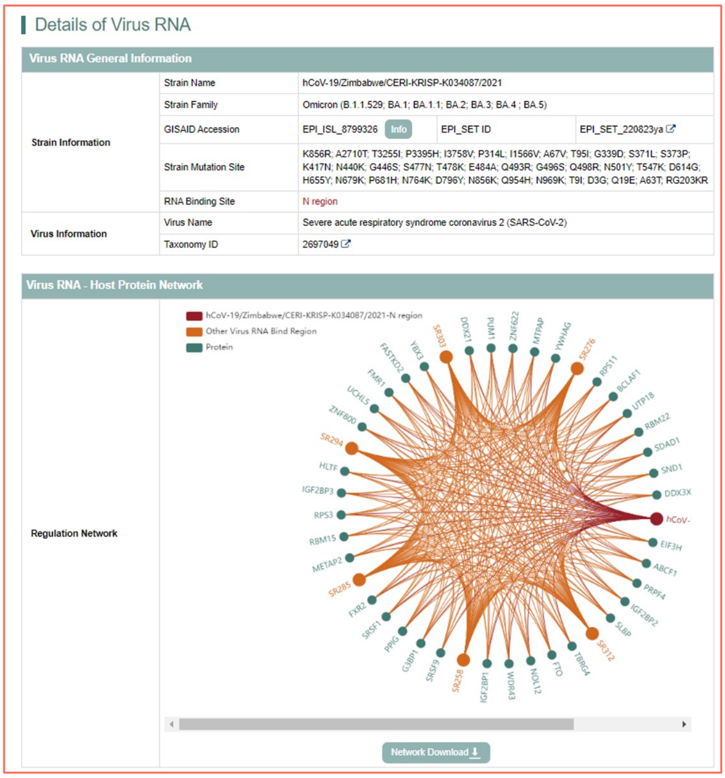

①

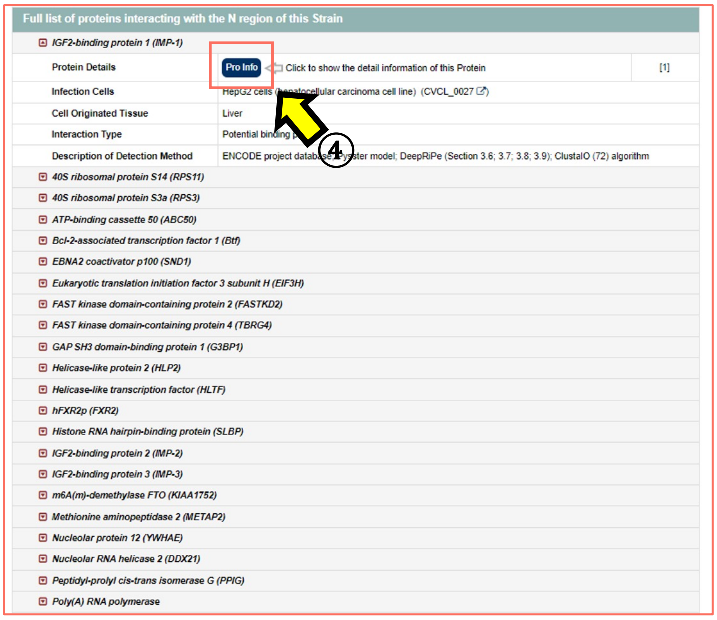

②

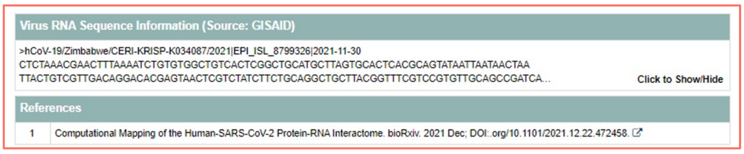

③

① Virus RNA General Information, Virus RNA - Host Protein Network
② Full list of proteins interacting with the N region of this Strain
③ Virus RNA Sequence Information (Source: GISAID), References
④ Click on Protein info.

CovInter

Main functions: Visualization, Data analysis tool, treatment

Details of Host Protein

Host Protein General Information (ID: PT0569)			
Protein Name	IGF2-binding protein 1 (IMP-1)	Gene Name	IGF2BP1
Host Species	Homo sapiens	Uniprot Entry Name	IF2B1_HUMAN
Protein Families	RRM IMP/VICKZ family		
Subcellular Location	Nucleus		
External Link	NCBI Gene ID	10642	
	Uniprot ID	Q9NZI8	
	Ensembl ID	ENSG00000159217	
	HGNC ID	HGNC:28866	
Function in Host	RNA-binding factor that recruits target transcripts tocytoplasmic protein-RNA complexes (mRNPs). This transcript 'caging'into mRNPs allows mRNA transport and transient storage. It alsomodulates the rate and location at which target transcripts encounterthe translational apparatus and shields them from endonuclease attacksor microRNA-mediated degradation. Prefe Click to Show/Hide		
Related KEGG Pathway	MicroRNAs in cancer	hsa05206	Pathway Map
3D Structure	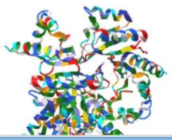	PDB ID: 3KRM<br>FASTA Download<br>2D PNG Download<br>PDB File Download	

Color Legend
Color by Residue
A (Ala) G (Gly) L (Leu) Q (Gln) V (Val) D (Asp) H (His) M (Met) R (Arg) Y (Tyr) E (Glu) I (Ile) N (Asn) S (Ser) F (Phe) K (Lys) P (Pro) T (Thr)

Feedback

④-1

Function of This Protein During Virus Infection				
Virus Name	SARS-COV-2	Protein Function	Pro-viral	[16]
Infected Tissue	Colon	Infection Time	48 h	
Infected Cell	Caco-2 cells (Human colorectal adenocarcinoma cell)	Cellosaurus ID	CVCL_0025	
Method Description	To detect the role of host protein IGF2BP1 in viral infection, IGF2BP1 protein knockout Caco-2 cells were infected with SARS-COV-2 for 48 h , and the effects on infection was detected through qPCR.			
Results	It is reported that Knockdown of IGF2BP1 leads to the reduced the vRNA levels compared with control group.			

Host Protein - Virus RNA Network

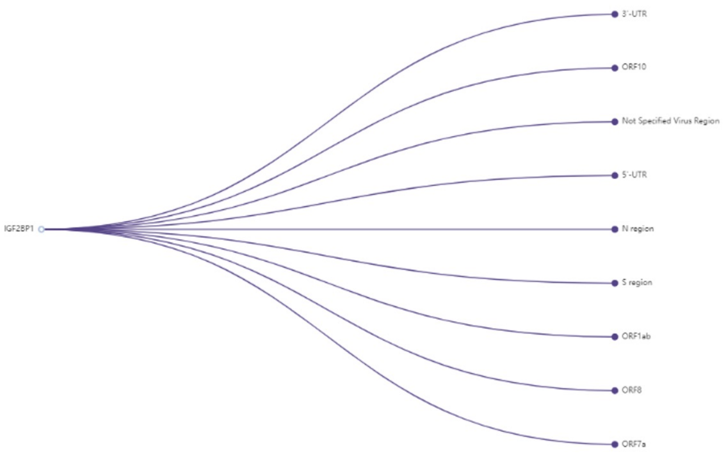

Network Download

④-2

④ Protein info.
④-1 Host Protein General Information
④-2 Function of This Protein During Virus Infection, Host Protein - Virus RNA Network

Main functions: Visualization, Data analysis tool, treatment

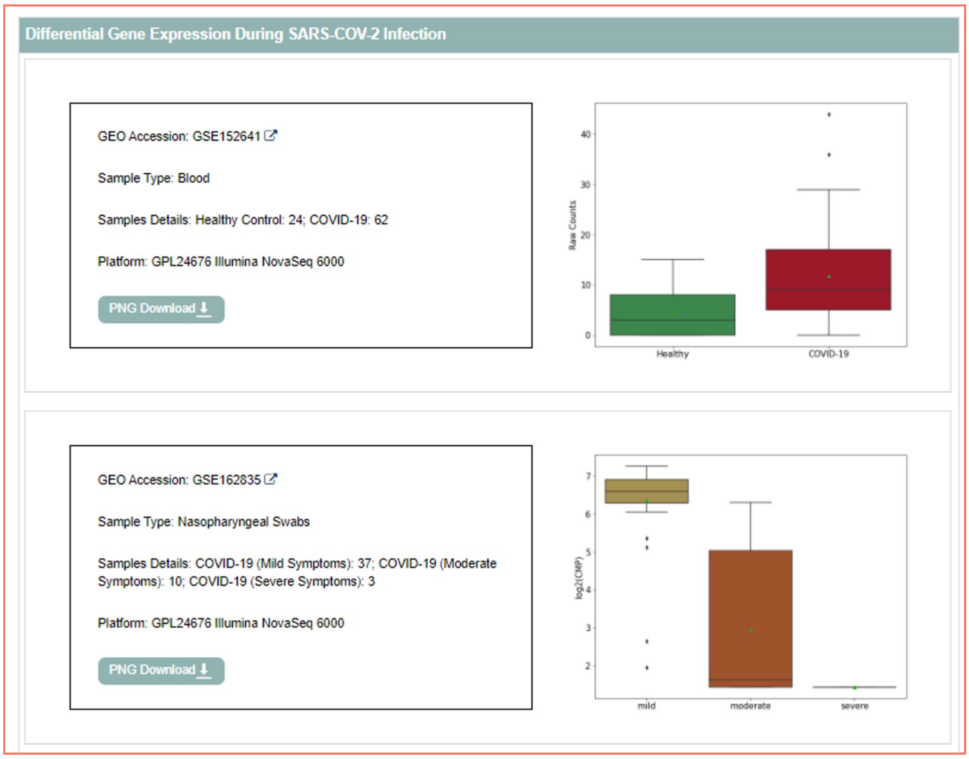

④-3

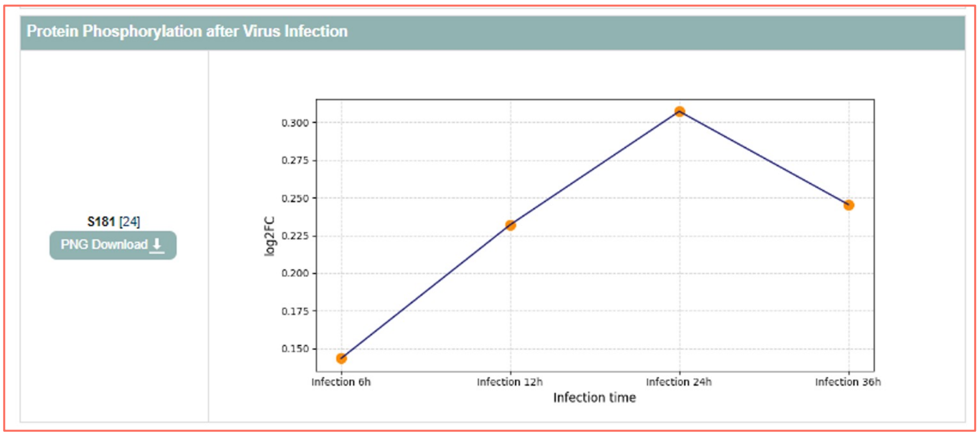

④-4

④ Protein info.
④-3 Differential Gene Expression During SARS-COV-2 Infection
④-4 Protein Phosphorylation after Virus Infection

CovInter

Main functions: Genome browser (sequence)

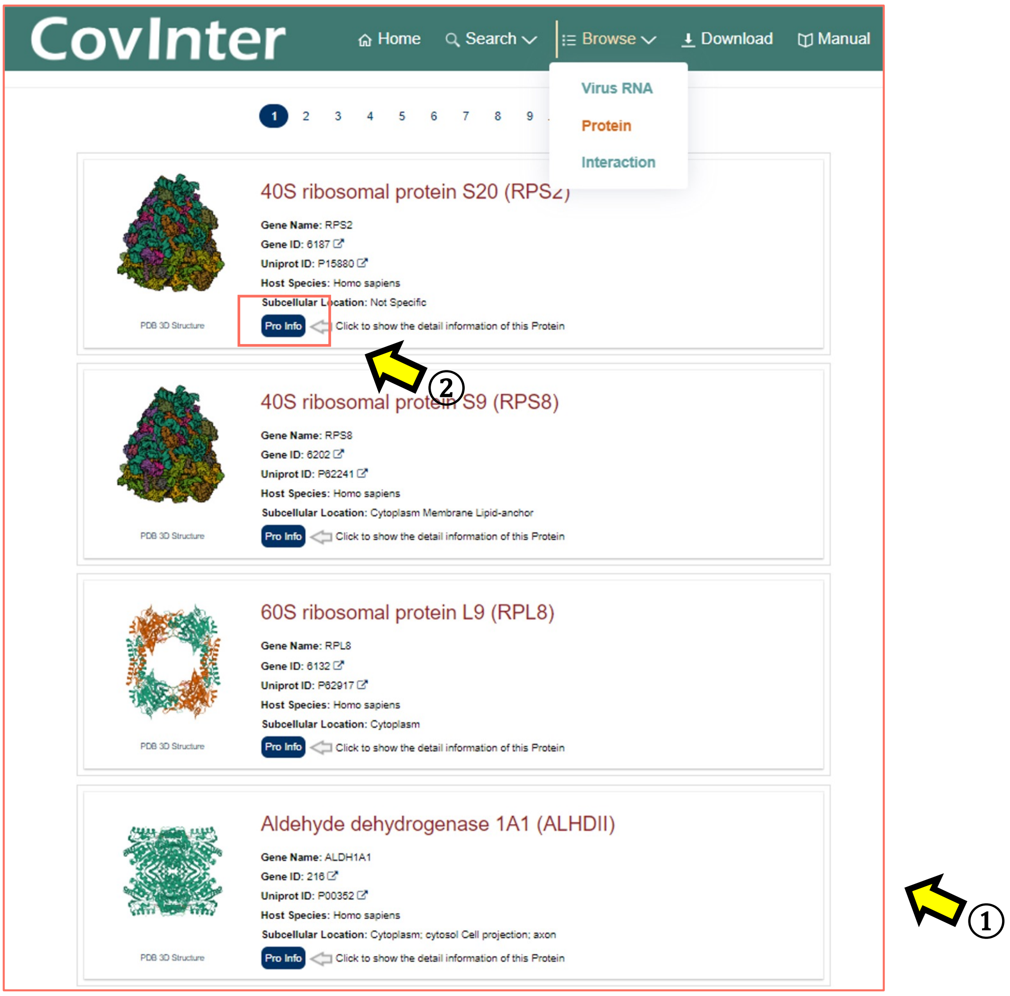

① **Protein**
② **Click on Detail Info**

Main functions: Visualization, Data analysis tool, treatment

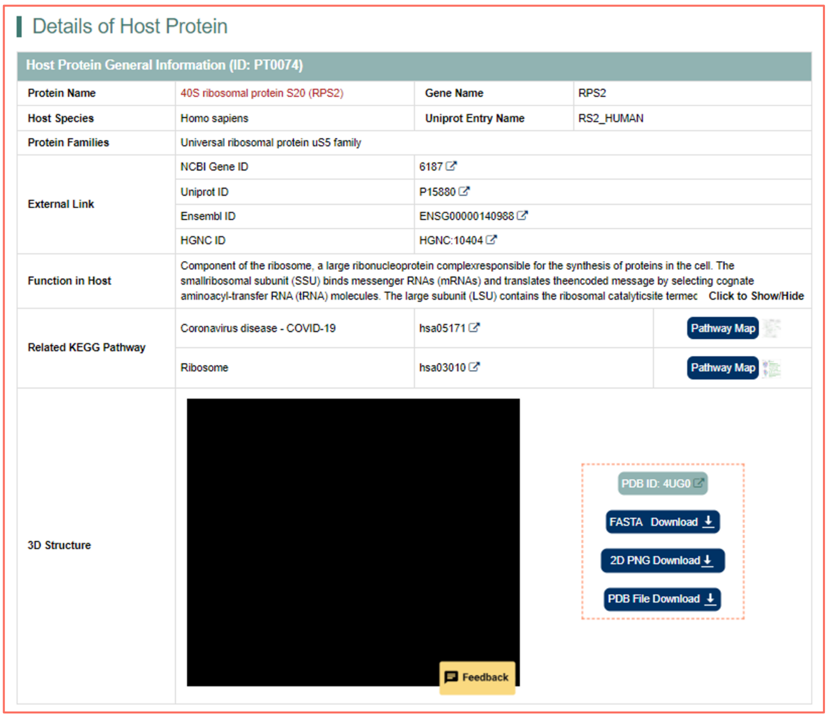

Details of Host Protein

Host Protein General Information (ID: PT0074)			
Protein Name	40S ribosomal protein S20 (RPS2)	Gene Name	RPS2
Host Species	Homo sapiens	Uniprot Entry Name	RS2_HUMAN
Protein Families	Universal ribosomal protein uS5 family		
External Link	NCBI Gene ID	6187	
	Uniprot ID	P15880	
	Ensembl ID	ENSG00000140988	
	HGNC ID	HGNC:10404	
Function in Host	Component of the ribosome, a large ribonucleoprotein complexresponsible for the synthesis of proteins in the cell. The smallribosomal subunit (SSU) binds messenger RNAs (mRNAs) and translates theencoded message by selecting cognate aminoacyl-transfer RNA (tRNA) molecules. The large subunit (LSU) contains the ribosomal catalyticsite termec Click to Show/Hide		
Related KEGG Pathway	Coronavirus disease - COVID-19	hsa05171	Pathway Map
	Ribosome	hsa03010	Pathway Map
3D Structure	PDB ID: 4UG0<br>FASTA Download<br>2D PNG Download<br>PDB File Download<br>Feedback		

①

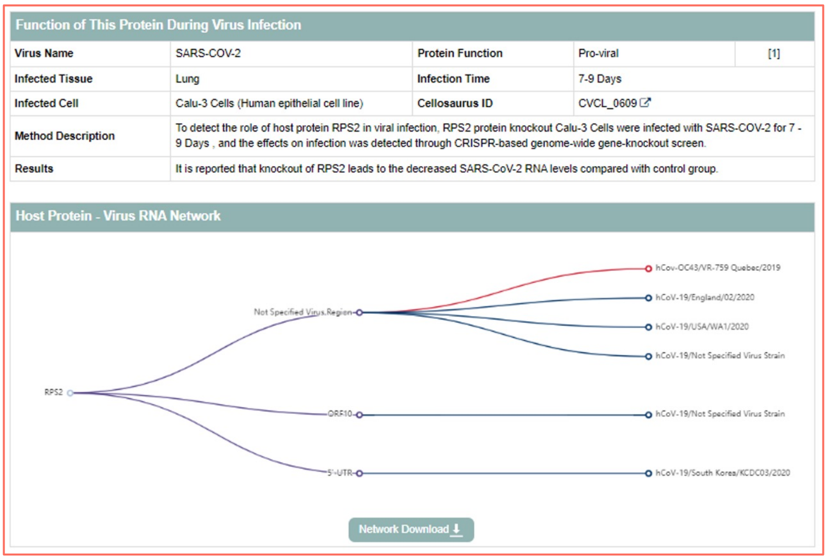

Function of This Protein During Virus Infection				
Virus Name	SARS-COV-2	Protein Function	Pro-viral	[1]
Infected Tissue	Lung	Infection Time	7-9 Days	
Infected Cell	Calu-3 Cells (Human epithelial cell line)	Cellosaurus ID	CVCL_0609	
Method Description	To detect the role of host protein RPS2 in viral infection, RPS2 protein knockout Calu-3 Cells were infected with SARS-COV-2 for 7 - 9 Days , and the effects on infection was detected through CRISPR-based genome-wide gene-knockout screen.			
Results	It is reported that knockout of RPS2 leads to the decreased SARS-CoV-2 RNA levels compared with control group.			

Host Protein - Virus RNA Network

②

① **Host Protein General Information**
② **Function of This Protein During Virus Infection**

CovInter

Main functions: Visualization, Data analysis tool, treatment

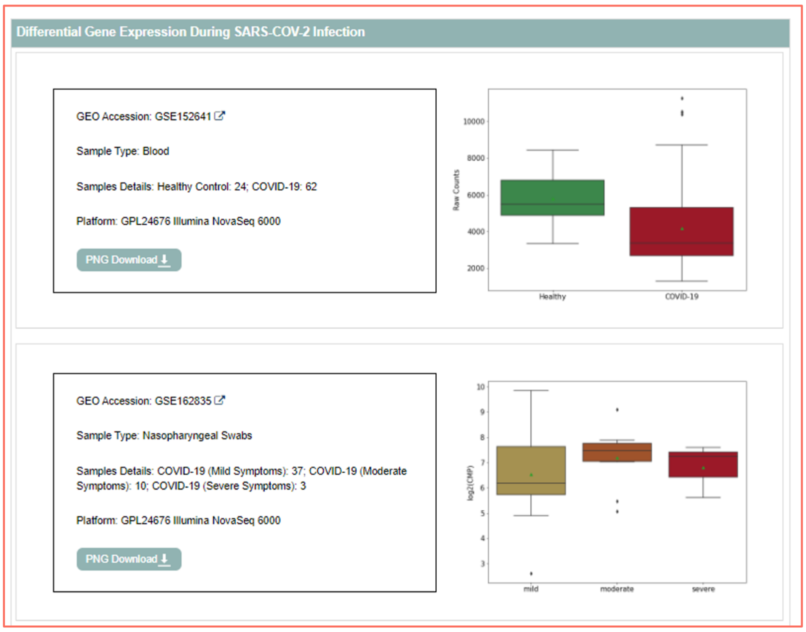

①

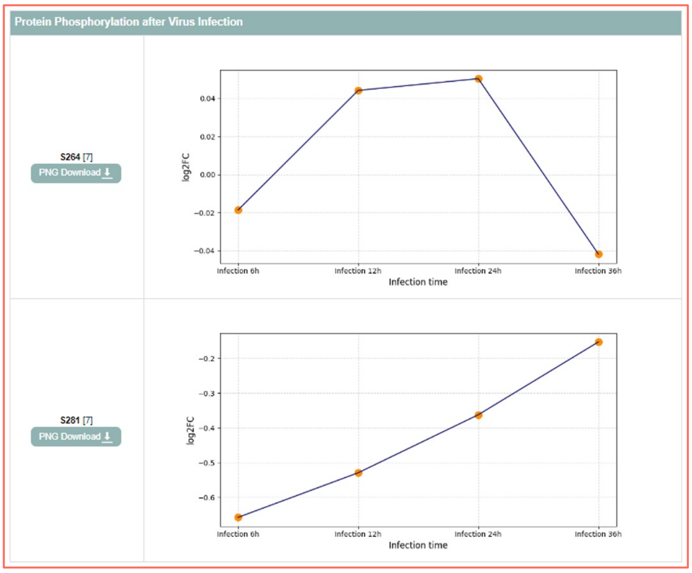

②

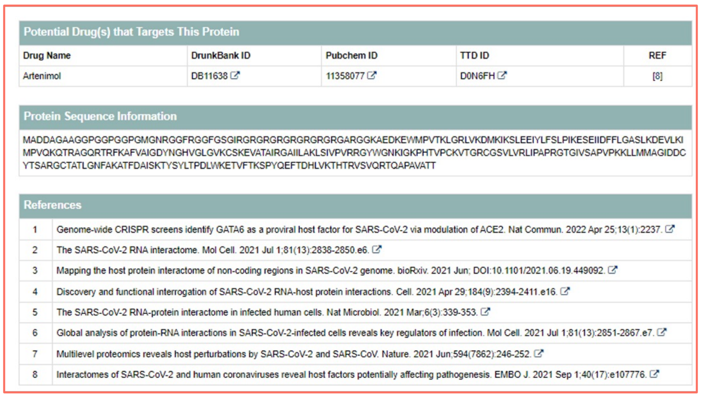

③

① Differential Gene Expression During SARS-COV-2 Infection
② Protein Phosphorylation after Virus Infection
③ Potential Drug(s) that Targets This Protein, Protein Sequence Information, References

CovInter

Main functions: Genome browser (sequence)

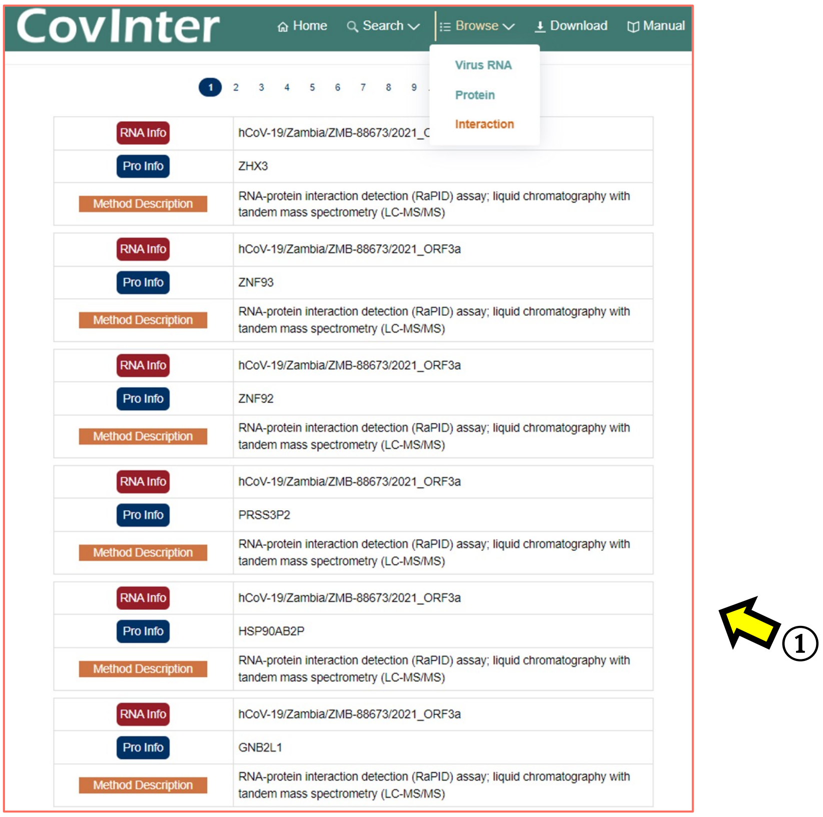

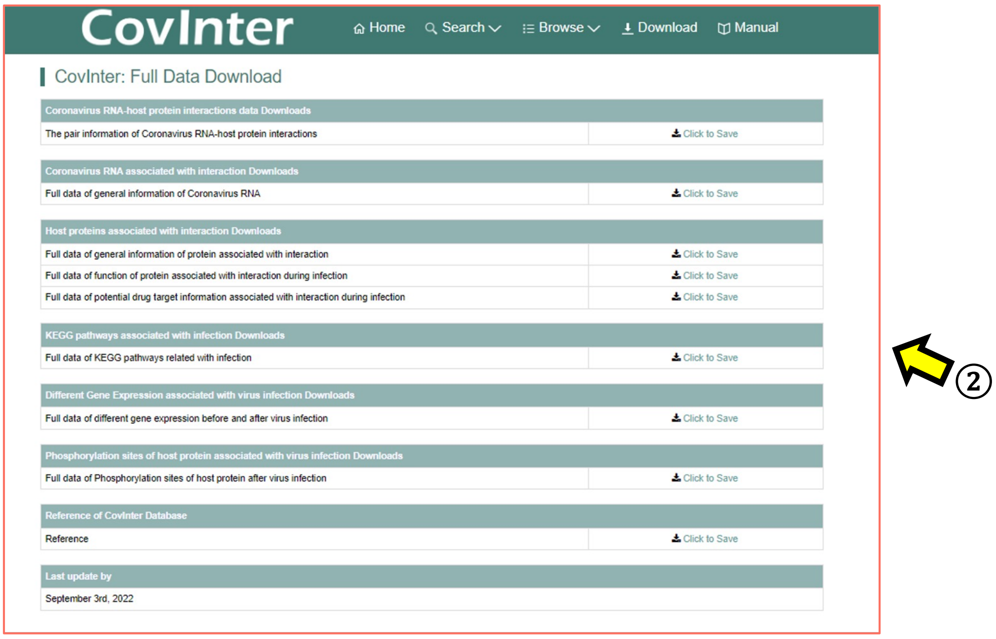

① Interaction
② Download
